# Hydrophilic magnetic COFs: The Answer to photocatalytic degradation and removal of imidacloprid insecticide^☆^

**DOI:** 10.1016/j.heliyon.2024.e39042

**Published:** 2024-10-11

**Authors:** Shaikha S. AlNeyadi, Mohammed T. Alhassani, Muneb R. Mukhtar, Hamad K. Alblooshi, Sultan A. Jama, Ibrahim Al Mujaini, Ali S. Aleissaee

**Affiliations:** Department of Chemistry College of Science, UAE University Al-Ain, 15551, United Arab Emirates

**Keywords:** Adsorption capacity, Magnetic COF, Photocatalytic degradation, Imidacloprid

## Abstract

The widespread use of imidacloprid (IMI) in pest control presents significant environmental challenges due to its persistence and low removal efficiency. This study introduces magnetic Covalent Organic Frameworks (COFs) functionalized with Fe₃O₄ nanoparticles (Fe₃O₄@HMN-COF, Fe₃O₄@MAN-COF, and Fe₃O₄@SIN-COF) as efficient adsorbents for IMI removal from water. These COFs, engineered with nitrogen-rich structures and extensive π-electron systems, achieve superior adsorption through π-π interactions, hydrophobic interactions, and hydrogen bonding. Characterization via FT-IR, XRD, and nitrogen sorption isotherms confirmed their high hydrophilicity, stability, and large surface areas. The magnetic properties of the COFs facilitated easy separation from water, enhancing practicality. Kinetic studies for all COFs indicated a pseudo-second-order model, suggesting chemisorption, with adsorption capacities of 600 mg/g for Fe₃O₄@HMN-COF, 480 mg/g for Fe₃O₄@MAN-COF, and 375 mg/g for Fe₃O₄@SIN-COF. Thermodynamic analyses revealed spontaneous and endothermic adsorption processes. Reusability tests showed minimal capacity loss over multiple cycles, underscoring their practical applicability. Practical tests in honey and fruit samples confirmed high efficacy, demonstrating the COFs' versatility. The study also optimized the photocatalytic degradation of imidacloprid using these COFs, with Fe₃O₄@HMN-COF achieving 98.5 % efficiency under optimal conditions (10 mg L^−1^ IMI, 0.01 g catalyst dose, pH 11, 30 °C, UV light). These findings highlight the potential of magnetic COFs for sustainable environmental remediation of pesticide-contaminated water.

## 1 Introduction

**Fig. 1:**
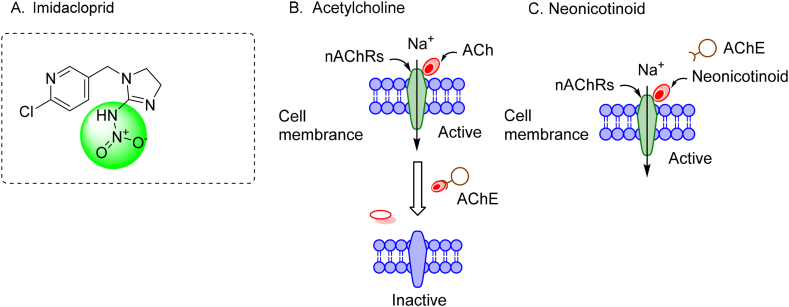
A) Imidacloprid structure; B-C) Illustrates the functioning of neonicotinoid acetylcholine receptors in the presence of both acetylcholine and a neonicotinoid substance, as referenced in Chang et al.'s work from 2013 [12]. Key physicochemical properties of Imidacloprid (IMI) include water solubility of 0.61 g/L at 20 °C, stability in acidic/neutral pH with slow hydrolysis in alkaline conditions, a log *P* of 0.57 (octanol/water), a half-life ranging from 48 to 190 days in soil and 28–1250 days in water, slow hydrolysis in water that accelerates under alkaline conditions, a molecular weight (MW) of 255.7 g/mol, four hydrogen acceptors (HA), one hydrogen donor (HD), and a three-dimensional size of 11.73 × 8.65 × 7.18 Å [13].

Agriculture and agro-industries heavily rely on the pesticide industry to meet the increasing demands for food production, spurred by a growing population and agricultural commercialization [1]. The pressure on fertile land for food and the need for bio-based products like biofuels intensify with the population surge. Pest attacks exacerbate crop losses, necessitating the inevitable reliance on pesticides for minimizing losses and achieving higher productivity. Pesticides find use not only in agriculture but also in animal husbandry, domestic settings, gardens, medicine, disease management, forestry, and public areas [2]. The utilization of conventional insecticides in the past, such as organophosphates, organochlorines, and carbamates, led to significant environmental issues and harm to various organisms [3]. Consequently, due to their toxicity and insect resistance, many of these conventional insecticides have been phased out [4]. This prompted the introduction of a new generation of insecticides, including neonicotinoids, oxadiazines, fiproles, pyrroles, and benzenedicarboxamides, known for their broader specificity and lower application rates [5]. Neonicotinoids, in particular, have become the most frequently used insecticides worldwide, accounting for over 25 % of the global insecticide market and generating a market value exceeding $2.6 billion due to their high efficiency and broad-spectrum pest control capabilities [6]. Imidacloprid (IMI) (Fig. 1A), one of the most commonly used neonicotinoids, represents 41 % of neonicotinoid usage. It has been registered in over 120 countries and is used on about 140 crops (e.g., maize, rice, cotton, and potatoes) to control pests like planthoppers, Aphis gossypii, and Nilaparvata lugens [7]. IMI functions by targeting the nicotinic acetylcholine receptor, damaging the central nervous system of insects, leading to abnormal behavior and death (Fig. 1B–C) [8]. Imidacloprid (IMI), a widely used chlorinated organic insecticide, poses significant environmental challenges due to its persistence and accumulation in water environments. IMI's half-life ranges from 28 to 1250 days [9], allowing it to persist in agricultural regions' surface and groundwater in America and Canada [7]. Its extensive application and persistence mean IMI is frequently detected in these water bodies. IMI is highly toxic to honeybees and aquatic invertebrates and poses potential adverse effects on human health [10]. Due to its high toxicity, persistence, and solubility, IMI has garnered increasing attention and has been added to the European Surface Water Watch List [11]. IMI is a systemic insecticide that permeates the entire plant—root, stem, leaves, flowers, pollen, and nectar—providing extensive and long-lasting defense against pests [4]. However, these insecticides exhibit remarkable persistence, lingering in irrigation systems, soil, and even the bodies of deceased bees, presenting prolonged risks and potential harm to pollinator insects and ecosystems. Given the escalating presence of imidacloprid residues in honey and fruits, urgent research is needed for efficient strategies to eliminate these pollutants [5].

**Fig. 2:**
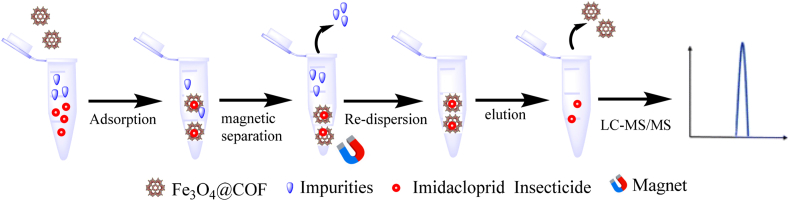
Schematic representation of the adsorption, magnetic separation, re-dispersion, elution, and LC-MS/MS (Liquid Chromatography with Tandem Mass Spectrometry) analysis process for Imidacloprid using Fe₃O₄-functionalized COFs. This sequence demonstrates the steps involved in capturing and analyzing Imidacloprid from samples, highlighting the efficiency and functionality of the COF-based magnetic separation technique.

In recent years, various techniques such as Fenton oxidation [14], biodegradation, advanced oxidation processes (AOPs) [15], electrochemical methods, and adsorption have been explored for eliminating imidacloprid (IMI) from soil and water [16]. Among these, adsorption stands out for its convenience, safety, and effectiveness. However, conventional adsorbent materials have limitations; for example, carbon materials exhibit limited adsorption capacity [17], zeolites face pore size issues [18], and metal–organic frameworks (MOFs) suffer from stability issues [19]. Covalent Organic Framework (COF) materials, composed of light elements like C, H, O, N, and B, offer promising advantages due to their exceptional stability, designable porosity, and high surface area [20]. Despite their potential, research on the adsorption and elimination of imidacloprid (IMI) by Covalent Organic Frameworks (COFs) remains limited. IMI, characterized by a low octanol/water partition coefficient, necessitates the use of hydrophilic adsorbents for effective extraction [21]. However, many COF materials are inherently hydrophobic, which restricts their capability to adsorb polar compounds. To overcome this limitation, incorporating functional groups such as nitro, amino, carboxyl, and hydroxyl groups can significantly enhance the hydrophilicity of COFs. These modifications facilitate strong interactions with analytes, thereby improving adsorption efficiency. Magnetic Covalent Organic Frameworks (magnetic COFs) provide a versatile solution by integrating COFs with magnetic particles, enhancing both separation efficiency and reusability. The addition of magnetic nanoparticles not only enables easy recovery of the adsorbent but also boosts adsorption capacity by offering additional active sites [21]. This study explores these advancements, aiming to establish hydrophilic magnetic COFs as effective agents for the removal of IMI from aqueous solutions. However, while adsorption provides an effective method for the initial removal of pollutants, it does not degrade the contaminants, potentially leading to secondary pollution. To address this, combining adsorption with photocatalytic degradation offers a comprehensive solution. Photocatalytic degradation has emerged as a highly effective method for removing organic pollutants, including pesticides like imidacloprid, from environmental matrices. Traditional photocatalysts such as TiO₂ and ZnO are widely studied due to their high efficiency, chemical stability, and strong oxidative power. However, their efficiency can be limited by the recombination of photogenerated electron-hole pairs and the requirement for UV light, which constitutes only a small fraction of the solar spectrum [22]. To overcome these limitations, researchers have developed various modifications, such as doping TiO₂ with metals like silver or non-metals like nitrogen, to extend its photoresponse to the visible light region, thereby enhancing photocatalytic efficiency [23,24]. Recent advancements have introduced magnetic covalent organic frameworks (COFs) as promising photocatalysts due to their high surface area, structural tunability, and stability [[25], [26], [27]]. The integration of magnetic nanoparticles into COFs creates a hybrid material that enhances photocatalytic activity and facilitates easy recovery and reuse through magnetic separation. Studies have shown that magnetic COFs exhibit excellent photocatalytic properties due to their high surface area, which provides numerous active sites for adsorption and degradation of pollutants, and the combination with magnetic particles improves electron-hole separation, enhancing ROS generation [28]. In the context of imidacloprid degradation, the hydrophilic nature of functionalized COFs allows for better interaction with polar imidacloprid molecules, facilitating efficient adsorption and degradation, while the magnetic properties enable easy separation of the catalyst from the reaction mixture, making the process more efficient and sustainable [[29], [30], [31]]. Our study introduces an innovative approach for adsorption and degrading imidacloprid using hydrophilic magnetic COFs. As illustrated in Fig. 2, the process encompasses several critical steps: adsorbing imidacloprid onto the magnetic COFs, employing magnetic separation to isolate the magnetic COFs, and eluting the pesticide for analysis via Liquid Chromatography-Mass Spectrometry (LC-MS). Furthermore, we will evaluate their performance in real samples, such as honey and fruit, to determine their practical applicability. Additionally, we will assess their effectiveness in the photocatalytic degradation of imidacloprid, capitalizing on the high surface area, structural tunability, and magnetic properties of COFs to enhance the overall degradation process.

## 2 Results and discussion

### 2.1 Synthesis and Characterization of COFs

**Fig. 3:**
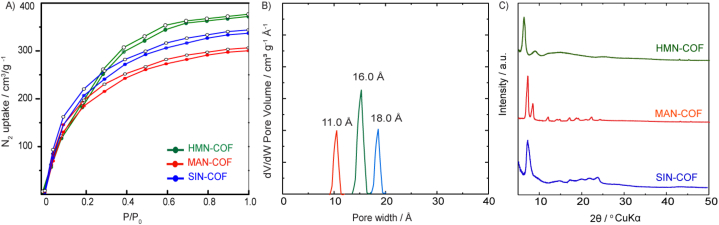
(A) Nitrogen adsorption-desorption isotherms for HMN-COF, MAN-COF, and SIN-COF, indicating their surface area and porosity. (B) Pore size distribution of the COFs, showing distinct pore widths for each framework. (C) XRD patterns of HMN-COF, MAN-COF, and SIN-COF, confirming their crystalline structures.

Designing COFs with highly effective Imidacloprid insecticide adsorption capacity critically depends on selecting the right linkers. The chosen linkers for preparing COFs were selected for their unique chemical properties, making them ideal for adsorbing Imidacloprid insecticides. The triazine-based linker *4,4′,4''-(1,3,5-triazine-2,4,6-triyl)tribenzaldehyde* offers high nitrogen content, enhancing affinity for Imidacloprid through hydrogen bonding and π-π interactions, while its aromatic structure provides a high surface area and porosity. The *2,5-bis(2-methoxyethoxy)terephthalohydrazide* linker contains hydrophilic methoxyethoxy groups that improve the COF's hydrophilicity, making it more effective at adsorbing polar Imidacloprid and offers multiple binding sites through the terephthalohydrazide moiety. The biphenyl-based linker *4,4′-diamino-[1,1′-biphenyl]-2,2′-dicarboxylic acid* provides rigidity and stability, with amino and carboxylic acid groups enhancing interactions with Imidacloprid via hydrogen bonding and electrostatic interactions. The *2,4,6-trihydroxybenzene-1,3,5-tricarbaldehyde* linker increases the number of active sites for binding Imidacloprid, improving adsorption capacity, and forms a robust framework. Lastly, the *3,3′-dihydroxy-[1,1′-biphenyl]-4,4′-dicarbaldehyde* linker introduces hydroxyl groups, enhancing hydrophilicity and aiding in the adsorption of polar compounds, while the biphenyl structure ensures stability and rigidity. We designed and created three COFs with outstanding stability and porosity by Schiff base reactions [32,33]. The detailed synthesis methods for organic linkers, including reagents, reaction conditions, and purification techniques, are outlined in the supporting data (SI, Section S2, Scheme S1-S5, and Section 3, Figs. S1–S5). A solvothermal technique was employed to create the three targeted COFs, using acetic acid as a catalyst. The building blocks were suspended in a mixture of solvents, either mesitylene/1,4-dioxane or *o*-dichlorobenzene/n-butanol, and exposed to solvothermal conditions at 120 °C for five days (SI, Section S4). Comprehensive characterization techniques, such as spectroscopic analysis, confirm the successful synthesis and stability of the COFs. The successful formation of imine bonds in MAN-COF, HMN-COF, and SIN-COF was confirmed through FT-IR spectroscopy, which displayed strong stretching vibrations of the C=N unit in the range of 1628–1635 cm⁻^1^. The spectra further indicated the effectiveness of the Schiff-base condensation process by showing the disappearance of the amino stretching vibrations from the amine linker and the C=O vibration from the aldehyde linker (SI, Section S5, Fig. S6). Specifically, the FT-IR spectrum of HMN-COF demonstrated the absence of characteristic stretching bands of the amine (Vas–NH₂, 3399 cm⁻^1^; Vs–NH₂, 3323 cm⁻^1^) and aldehyde (HC═O, 1652 cm⁻^1^) after the polycondensation reaction, confirming no residual starting materials. The new stretching vibration band at 1635 cm⁻^1^ was attributed to the imine linkages. Stability is crucial for COFs in practical applications. We synthesized MAN-COF, HMN-COF, and SIN-COF, demonstrating their exceptional chemical and thermal stability. TGA showed they maintained structural integrity up to 400 °C in nitrogen. Chemical stability was confirmed over 24 h in various solvents, including boiling water, ethanol, *N,N*-dimethylformamide, dimethyl sulfoxide, 3 M HCl, and 3 M NaOH. PXRD patterns remained unchanged, confirming their robustness (SI, Section S5, Fig. S7). These results highlight the suitability of these COFs for various applications. The attractiveness of these COFs as chemically and thermally stable materials is further enhanced by their crystalline structure and porous features. Using nitrogen sorption isotherms at 77 K, we examined the porous architectures of MAN-COF, HMN-COF, and SIN-COF (Fig. 3A). Before the nitrogen sorption measurements, the COF samples were pre-treated overnight at 100 °C under vacuum. The nitrogen adsorption of MAN-COF, HMN-COF, and SIN-COF increased rapidly at lower pressures (P/P₀ = 0 to 0.1), indicating their microporous characteristics. The Brunauer-Emmett-Teller (BET) surface areas of MAN-COF, HMN-COF, and SIN-COF were determined to be 840 m^2^/g, 910 m^2^/g, and 670 m^2^/g, respectively. At P/P₀ = 0.99, used to compare the total pore volumes, HMN-COF showcased an impressive pore volume of 1.10 cm³/g, surpassing SIN-COF and MAN-COF, which had pore volumes of 0.63 cm³/g and 0.58 cm³/g, respectively. The pore sizes of MAN-COF, HMN-COF, and SIN-COF, computed using nonlocal density functional theory (NLDFT), were found to be 11 Å, 18 Å, and 15 Å, respectively (Fig. 3B). The crystalline characteristics of SIN-COF, HMN-COF, and MAN-COF were investigated through powder X-ray diffraction (PXRD) analysis, as illustrated in Fig. 3C. For SIN-COF, distinct diffraction peaks at 4.642°, 15.136°, 20.743°, 22.235°, 12.439°, and 25.834° confirmed its high crystallinity. Similarly, HMN-COF displayed prominent peaks at 4.813°, 7.035°, 15.122°, and 22.532°, indicating a high degree of crystallinity. MAN-COF also exhibited significant diffraction peaks at 7.314°, 8.065°, 11.321°, 15.543°, 17.352°, 20.743°, and 23.034°, demonstrating its highly crystalline nature.

### 2.2 Synthesis and Characterization of Magnetic COFs

**Fig. 4:**
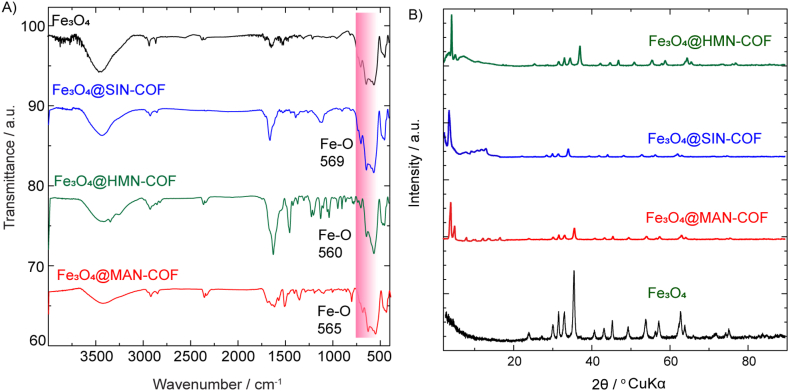
(A) FTIR spectra of Fe₃O₄ and Fe₃O₄@COF composites showing characteristic Fe-O stretching vibrations at 569 cm⁻^1^, 560 cm⁻^1^, and 565 cm⁻^1^ for Fe₃O₄@SIN-COF, Fe₃O₄@HMN-COF, and Fe₃O₄@MAN-COF, respectively. (B) XRD patterns of Fe₃O₄ and Fe₃O₄@COF composites indicating the successful encapsulation of Fe₃O₄ nanoparticles within the COF structures with distinct crystallinity peaks at various 2θ angles.

The successful formation of magnetic covalent organic frameworks (COFs) can be confirmed through a combination of spectroscopic, diffraction, and surface area analyses (SI, Scheme S9). The incorporation of Fe_3_O_4_ nanoparticles within the COF structures not only imparts magnetic properties but also affects the physical characteristics such as surface area and pore volume. In this discussion, we will explore the evidence supporting the formation of Fe_3_O_4_@10.13039/100013859COF composites. The IR spectra of the three magnetic covalent organic frameworks (COFs)— Fe_3_O_4_@HMN-COF, Fe_3_O_4_@MAN-COF, and Fe_3_O_4_@SIN-COF —show an additional characteristic band in the region of 560–569 cm^−1^. This band is attributed to the Fe–O vibration, a clear indicator of the presence of iron oxide within the COF structures. The appearance of this Fe–O vibration band confirms that the COFs have been successfully magnetized (Fig. 4A). The crystalline structure and phase purity of the samples were characterized via X-ray diffraction (XRD) pattern analysis, as shown in Fig. 4B. The Fe_3_O_4_@HMN-COF, Fe_3_O_4_@MAN-COF, and Fe_3_O_4_@SIN-COF samples exhibit the same characteristic diffraction peaks as Fe_3_O_4_ within the range of 10°–70°, confirming the retention of the magnetic component's structure. Additionally, the presence of other diffraction peaks at low angles, characteristic of pure COFs, indicates the successful incorporation of the COF structure and confirms the magnetization of Fe_3_O_4_@HMN-COF, Fe_3_O_4_@MAN-COF, and Fe_3_O_4_@SIN-COF.

**Fig. 5:**
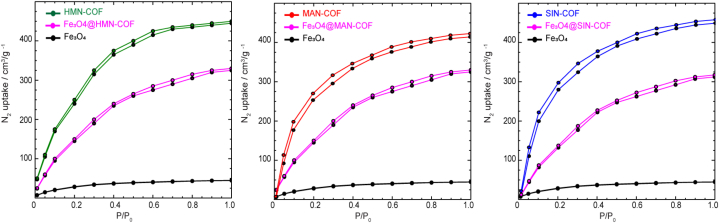
Nitrogen adsorption-desorption isotherms for HMN-COF, MAN-COF, SIN-COF, and their respective Fe₃O₄-functionalized counterparts (Fe₃O₄@HMN-COF, Fe₃O₄@MAN-COF, Fe₃O₄@SIN-COF), compared to Fe₃O₄. Each COF shows enhanced N₂ uptake compared to Fe₃O₄ alone, demonstrating significant improvements in surface area and porosity upon functionalization.

The incorporation of Fe_3_O_4_ nanoparticles into the COFs significantly impacts the BET surface area due to the occupation of pore space by the nanoparticles. Fe_3_O_4_ nanoparticles typically exhibit a much lower surface area, around 200 m^2^/g, due to their non-porous or less porous nature compared to COFs. This inherent difference in surface area affects the overall BET surface area of the composite materials. The BET surface areas of pure COFs—HMN-COF, MAN-COF, and SIN-COF—were found to be 910 m^2^/g, 840 m^2^/g, and 670 m^2^/g, respectively. Upon incorporating Fe_3_O_4_ nanoparticles, the BET surface area of Fe_3_O_4_@HMN-COF decreased significantly, typically to around 680 m^2^/g. Similarly, the surface areas of Fe_3_O_4_@MAN-COF and Fe_3_O_4_@SIN-COF also showed reductions, with Fe_3_O_4_@MAN-COF typically ranging around 580 m^2^/g and Fe_3_O_4_@SIN-COF around 490 m^2^/g. These results confirm the successful integration of Fe_3_O_4_ within the COF structures, leading to a decrease in surface area due to the lower surface area of Fe_3_O_4_ nanoparticles. The observed reductions in BET surface area reflect the combination of high surface area COFs and the comparatively low surface area of the incorporated Fe_3_O_4_, demonstrating the structural and compositional changes within the composite materials (Fig. 5).

**Fig. 6:**
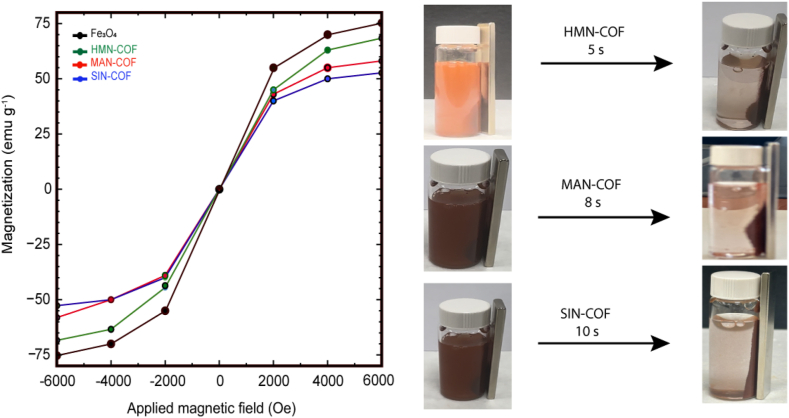
Magnetization curves of Fe₃O₄, Fe₃O₄@HMN-COF, Fe₃O₄@MAN-COF, and Fe₃O₄@SIN-COF as a function of the applied magnetic field (Oe). Images on the right show the magnetic separation times for Fe₃O₄@HMN-COF, Fe₃O₄@MAN-COF, and Fe₃O₄@SIN-COF, indicating rapid magnetic response with separation times of 8 s, 10 s, and 12 s, respectively.

The Vibrating Sample Magnetometer (VSM) measures the magnetic properties of materials by subjecting them to a varying magnetic field and monitoring the induced magnetic response. Essentially, the VSM assesses the magnetization of a sample as a function of the applied magnetic field. Analysis using VSM reveals that Fe_3_O_4_ nanoparticles have a high saturation magnetization, with a maximum value of 75.3 emu/g. However, adding COF coatings reduces this magnetization, with Fe_3_O_4_@HMN-COF, Fe_3_O_4_@MAN-COF, and Fe_3_O_4_@SIN-COF showing saturation magnetization values of 68.2 emu/g, 52.7 emu/g, and 58.1 emu/g, respectively. This reduction is due to the non-magnetic nature of the COF coatings, which affect the composite microspheres' magnetic behavior. Despite this decrease, the COF-coated microspheres (Fe_3_O_4_@HMN-COF, Fe_3_O_4_@MAN-COF, and Fe_3_O4@SIN-COF) retain sufficient magnetic properties, enabling rapid collection from aqueous solutions within 5, 8, and 10 s, respectively, using an external magnet, as illustrated in Fig. 6. This capability highlights their potential for magnetic separation applications, such as environmental cleanup, biomedical separations, and catalyst recovery.

In addition, the deconvolution of the XPS spectra for Fe₃O₄@MAN-COF, Fe₃O₄@HMN-COF, and Fe₃O₄@SIN-COF shows peaks at 724.8 eV and 711.3 eV, which can be assigned to Fe in Fe 2p_1/2_ and Fe 2p_3/2_, respectively. Additionally, peaks at 732.0 eV and 716.8 eV are identified as the satellite peaks of Fe. These results indicate the successful construction of all three COFs (SI, Fig. S8).

Stability of Fe₃O₄ Nanoparticles under Acidic Conditions. To address concerns regarding the stability of Fe₃O₄ nanoparticles within the magnetic COFs composite, particularly under acidic conditions, we conducted an in-depth analysis using Inductively Coupled Plasma Atomic Absorption Spectroscopy (ICP-AAS)**.** The study aimed to detect the potential leaching of Fe³⁺ ions into the solution following the adsorption of imidacloprid, with a focus on the stability of the composite across a range of pH levels. The experimental results (SI, Table S1) demonstrate that the magnetic COF composite maintained significant chemical stability under neutral to strongly acidic conditions. Even at a pH of 1, the leaching of Fe³⁺ ions into the solution remained minimal, with a maximum recorded concentration of 0.50 ppm. At neutral and slightly acidic conditions (pH 7 and 5), the leaching was almost negligible, indicating a high degree of chemical robustness in less extreme environments. The results suggest that the Fe₃O₄ nanoparticles, integrated into the composite structure, are not significantly affected by changes in the pH of the solution. This stability is particularly important for practical applications in water treatment, where the magnetic COFs composite can be exposed to various chemical environments. The low levels of Fe³⁺ ion release reinforce the suitability of the material for repeated use without concerns of significant degradation or contamination of treated water. While the stability under acidic conditions is promising, the slight increase in Fe³⁺ leaching as the pH becomes more acidic (pH 3 and 1) suggests that further enhancements to the material's stability could be beneficial. One potential improvement involves coating the Fe₃O₄ nanoparticles with a protective layer, such as silica or a polymer, to further limit the potential for leaching under harsher conditions. This approach could extend the reusability of the COFs composite and improve their performance over long-term usage in highly acidic environments. In summary, the ICP-AAS data confirms that the magnetic COF composite exhibits strong chemical stability, making it a suitable candidate for environmental applications such as pesticide removal from water. The minimal Fe³⁺ leaching, even under acidic conditions, highlights the robustness of the material, with future modifications aimed at further enhancing this stability.

Following the structural characterization, water contact angle measurements were conducted to evaluate the hydrophilicity of the magnetic COFs, a critical factor influencing their performance in pollutant removal. The water contact angle measurements provide key insights into the hydrophilicity of the magnetic COFs and their impact on pollutant removal efficiency. As shown in Fig. S9, the contact angles for Fe₃O₄@HMN-COF, Fe₃O₄@MAN-COF, and Fe₃O₄@SIN-COF were 25°, 32°, and 28°, respectively. These low contact angle values confirm the highly hydrophilic nature of the magnetic COFs, with Fe₃O₄@HMN-COF exhibiting the most hydrophilic surface among the three samples. A lower contact angle indicates stronger interactions between the COF surface and the aqueous environment, directly contributing to the enhanced adsorption of polar pollutants such as imidacloprid. The hydrophilic surfaces of the COFs enable better wettability, allowing more effective interaction between the COFs and waterborne pollutants. This is particularly beneficial for the adsorption process, where efficient contact between the adsorbent and the target molecule is critical for higher removal efficiency. These results are consistent with previous reports on the role of surface hydrophilicity in facilitating pollutant adsorption by magnetic nanoparticles and covalent organic frameworks [34,35]. The enhanced hydrophilicity of these COFs likely stems from the presence of functional groups such as hydroxyl, carboxyl, and amino groups, which promote hydrogen bonding with water molecules and polar pollutants. In summary, the water contact angle measurements provide strong evidence that the hydrophilic nature of the magnetic COFs plays a significant role in the removal of imidacloprid, further validating the material's suitability for environmental remediation applications.

**Fig. 7:**
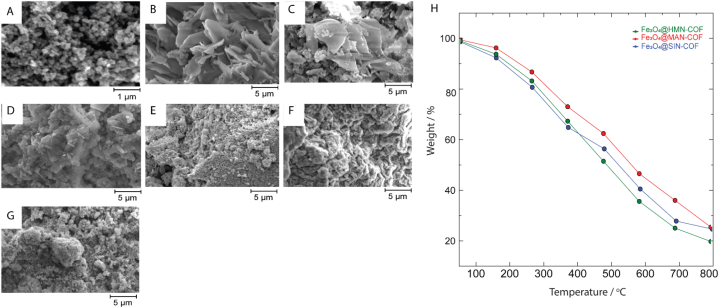
SEM images and TGA of Fe₃O₄-functionalized COFs. (A) Fe₃O₄ nanoparticles; (B) HMN-COF; (C) Fe₃O₄@HMN-COF; (D) MAN-COF; (E) Fe₃O₄@MAN-COF; (F) SIN-COF; (G) Fe₃O₄@SIN-COF. (H) TGA curves showing thermal stability and Fe₃O₄ loading (25–35 wt%) in Fe₃O₄@COFs.

The surface morphology of the three synthesized COFs (HMN-COF, MAN-COF, and SIN-COF) before and after the addition of Fe₃O₄ nanoparticles was investigated using Scanning Electron Microscopy (SEM). The SEM images for the pristine COFs reveal a rough, porous structure, typical of covalent organic frameworks (COFs), which is conducive to their application in adsorption and catalytic processes. These structures exhibit high surface area and interconnectivity, facilitating efficient pollutant capture and catalytic performance. After the incorporation of Fe₃O₄ nanoparticles, significant changes in the surface texture of the COFs were observed. The Fe₃O₄ nanoparticles were uniformly distributed across the surface of the COFs, as evidenced by the more granular appearance in the SEM images (Fig. 7A–G). While some degree of particle aggregation was noted, it was minimal, and the nanoparticles appeared well-integrated into the COF framework, maintaining the structural integrity of the original COF. Further characterization using Thermogravimetric Analysis (TGA) confirmed the successful incorporation of Fe₃O₄. The TGA profiles of the magnetic COFs showed a weight loss corresponding to the decomposition of the organic framework, with residual weights consistent with the presence of Fe₃O₄ nanoparticles. The Fe₃O₄ content in the magnetic COFs was determined to be approximately 25–35 wt%, depending on the COF (Fig. 7H). This variation in Fe₃O₄ loading reflects slight differences in the synthesis conditions but ensures that each magnetic COF retains sufficient magnetic functionality while maintaining the COF's high surface area and porosity. The residual mass observed at higher temperatures (>600 °C) corresponds to the stable Fe₃O₄ nanoparticles, which remain after the decomposition of the organic components. The combination of SEM and TGA analyses demonstrates that the magnetic COFs possess both the high surface area required for efficient pollutant adsorption and the magnetic functionality needed for easy separation. This balance between structure and function highlights the potential of these materials for environmental applications.

### 2.3 Adsorption experiments

After synthesizing the magnetic COFs (Fe₃O₄@MAN-COF, Fe₃O₄@HMN-COF, and Fe₃O₄@SIN-COF), we examined their adsorption properties for imidacloprid. The study focused on evaluating the efficiency and effectiveness of these COFs in adsorbing imidacloprid, aiming to highlight their potential application in environmental remediation.

#### 2.3.1 *Effect of pH*

**Fig. 8:**
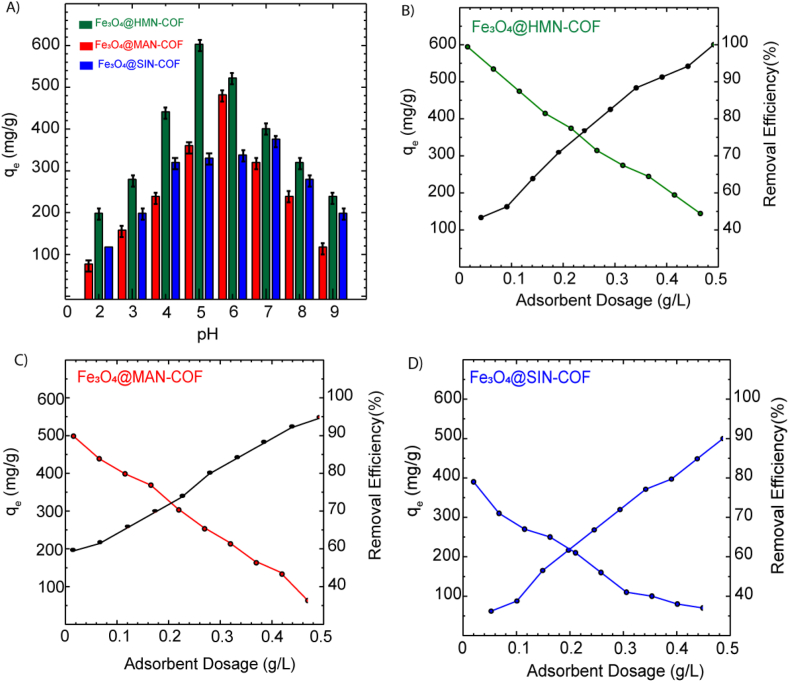
A) Effect of pH on the adsorption capacity (q_e_) of Fe₃O₄@HMN-COF, Fe₃O₄@MAN-COF, and Fe₃O₄@SIN-CO; B-D) Graphs showing the relationship between adsorbent dosage and both adsorption capacity (q_e_) and removal efficiency (%) for Fe₃O₄@HMN-COF, Fe₃O₄@MAN-COF, and Fe₃O₄@SIN-COF. Panel A) displays the data for Fe₃O₄@HMN-COF, Panel B) shows Fe₃O₄@MAN-COF, and Panel C) illustrates Fe₃O₄@SIN-COF. The x-axis represents the adsorbent dosage (g/L), while the left y-axis indicates the adsorption capacity (mg/g) and the right y-axis shows the removal efficiency (%). The trends depict a decrease in adsorption capacity and an increase in removal efficiency with increasing adsorbent dosage for each COF.

This investigation explores the influence of pH on the adsorption efficiency of imidacloprid, focusing on the interaction mechanisms between the COFs and the amphoteric nature of imidacloprid across a pH range of 2–9 employing LC-MS/MS to quantify residual concentrations post-adsorption. The electrostatic attraction or repulsion mainly depended on the surface charge of adsorbent and adsorbate species at different solution pH. The zero potential charge point (pH_ZPC_) of Fe₃O₄@MAN-COF, Fe₃O₄@HMN-COF, and Fe₃O₄@SIN-COF were 6.35, 5.07 and 7.09, respectively (SI, Fig. S9). When the solution pH below the pH_ZPC_ of adsorbent, its surface was protonated and positively charged, otherwise it was negatively charged. Imidacloprid, an amphoteric molecule, is hydrophilic (logKow = 0.57), with its dominant species being IMI^+^ at pH < 1.56, IMI^±^ at pH 1.56–11.12, and IMI^−^ at pH > 11.12. At pH levels below 1.56, imidacloprid predominantly exists as IMI^+^, the protonated form carrying a positive charge; between pH 1.56 and 11.12, it exists as IMI^±^, a mixture of protonated and neutral forms with a net charge close to neutral; and at pH levels above 11.12, imidacloprid predominantly exists as IMI^−^, the deprotonated form carrying a negative charge. Each COF features specific functional groups that influence its adsorption properties: Fe₃O₄@MAN-COF incorporates carboxyl groups and a triazine moiety; Fe₃O₄@HMN-COF contains hydroxyl groups and ether linkages; and Fe₃O₄@SIN-COF is characterized by biphenyl, hydroxyl, and carboxylic acid functionalities. For Fe₃O₄@MAN-COF, optimal adsorption of imidacloprid occurs at pH 6.35 due to near-neutral zeta potential, facilitating favorable π-π interactions and hydrogen bonding. At low pH (2–3), the less negative surface charge reduces electrostatic repulsion, enhancing adsorption through strong electrostatic interactions and hydrogen bonding with the positively charged IMI^+^. At higher pH (8–9), the shift towards the deprotonated IMI^−^ species increases electrostatic repulsion with the negatively charged COF surface, limiting adsorption efficiency. Fe₃O₄@HMN-COF exhibits optimal adsorption at pH 5.07 due to its balanced surface charge, promoting efficient adsorption through hydrogen bonding and π-π stacking with imidacloprid. At acidic pH levels (2–3), its less negative surface charge enhances interactions with IMI^+^ via hydrogen bonding and π-π stacking, increasing adsorption capacity. At higher pH levels (8–9), the increased negative surface charge enhances electrostatic repulsion with the negatively charged IMI^−^, reducing adsorption efficiency. Fe₃O₄@SIN-COF achieves peak adsorption efficiency at pH 7 due to near-neutral zeta potential, which minimizes electrostatic repulsion and maximizes adsorption through strong π-π interactions and hydrogen bonding. At low pH (2–3), its biphenyl and carboxylic acid groups facilitate favorable interactions, enhancing adsorption through π-π stacking and hydrogen bonding. At higher pH levels (8–9), the increasing negative surface charge leads to enhanced electrostatic repulsion with the deprotonated IMI^−^ species, diminishing adsorption efficiency despite the potential increase in adsorption sites. Fe₃O₄@HMN-COF is the most versatile and effective material for adsorbing imidacloprid, showing peak adsorption efficiency around pH 5 and performing well across a broad pH range (2–7). Its surface chemistry, with hydroxyl groups and ether linkages, facilitates hydrogen bonding and π-π stacking interactions with imidacloprid, balancing electrostatic forces effectively. This ensures robust adsorption across various pH levels, making it more adaptable to environmental fluctuations compared to Fe₃O₄@MAN-COF and Fe₃O₄@SIN-COF, which have narrower optimal pH ranges. Fe₃O₄@HMN-COF's ability to maintain efficient adsorption in diverse conditions highlights its potential for environmental remediation. Due to the slightly higher adsorption capability of Fe₃O₄@MAN-COF at pH 6, subsequent adsorption experiments were conducted at this pH. Similarly, Fe₃O₄@HMN-COF was tested at pH 5, and Fe₃O₄@SIN-COF at pH 7, where each demonstrated optimal adsorption (Fig. 8A).

#### 2.3.2 *Adsorbent Dosage*

The study explored the effects of different concentrations of each COF (ranging from 0.05 to 0.5 g/L) on their adsorption capacity and removal efficiency for imidacloprid. At low dosages of Fe₃O₄@MAN-COF, Fe₃O₄@HMN-COF, and Fe₃O₄@SIN-COF, the concentration of imidacloprid is high relative to the available adsorption sites, resulting in a high adsorption capacity as most imidacloprid molecules find adsorption sites. However, the total number of imidacloprid molecules removed from the solution is lower, leading to lower removal efficiency. Conversely, at higher dosages, there are more adsorption sites available than necessary for capturing the available imidacloprid, which means many sites remain unoccupied, thereby reducing the adsorption capacity (mg of imidacloprid per g of COF). Despite this, the increased number of adsorption sites enhances the probability of capturing nearly all the imidacloprid molecules present, thus significantly improving the removal efficiency. Fe₃O₄@HMN-COF showed a notable higher removal efficiency compared to Fe₃O₄@MAN-COF due to its surface chemistry facilitating more effective interactions with imidacloprid. Fe₃O₄@SIN-COF, although demonstrating the same inverse relationship between concentration and adsorption capacity, exhibited the lowest removal efficiency among the three COFs. This discrepancy is attributed to the different functional groups and surface charges of each COF. Considering both adsorption capacity and removal efficiency, the optimal solid-liquid ratios were determined to be 0.22 g/L for Fe₃O₄@MAN-COF, 0.25 g/L for Fe₃O₄@HMN-COF, and 0.20 g/L for Fe₃O₄@SIN-COF. These ratios ensure a balance between effective adsorption and practical application across varying environmental conditions. This behavior highlights the balance between adsorption capacity and removal efficiency across different dosages for Fe₃O₄@MAN-COF, Fe₃O₄@HMN-COF, and Fe₃O₄@SIN-COF (Fig. 8B–D).

#### 2.3.3 *Effect of Ionic Strength*

To verify the anti-interference ability of Fe₃O₄@HMN-COF, Fe₃O₄@MAN-COF, and Fe₃O₄@SIN-COF, salt solutions with varying concentrations (10–40 mg L^−1^) were added to the solution. As shown in Fig. S10, the adsorption capacity of imidacloprid decreased slightly but remained largely unchanged overall. This indicates that electrostatic effects do not play a significant role in the adsorption process [36], which is consistent with experimental results observed under different pH conditions.

#### 2.3.4 Adsorption Kinetics

**Fig. 9:**
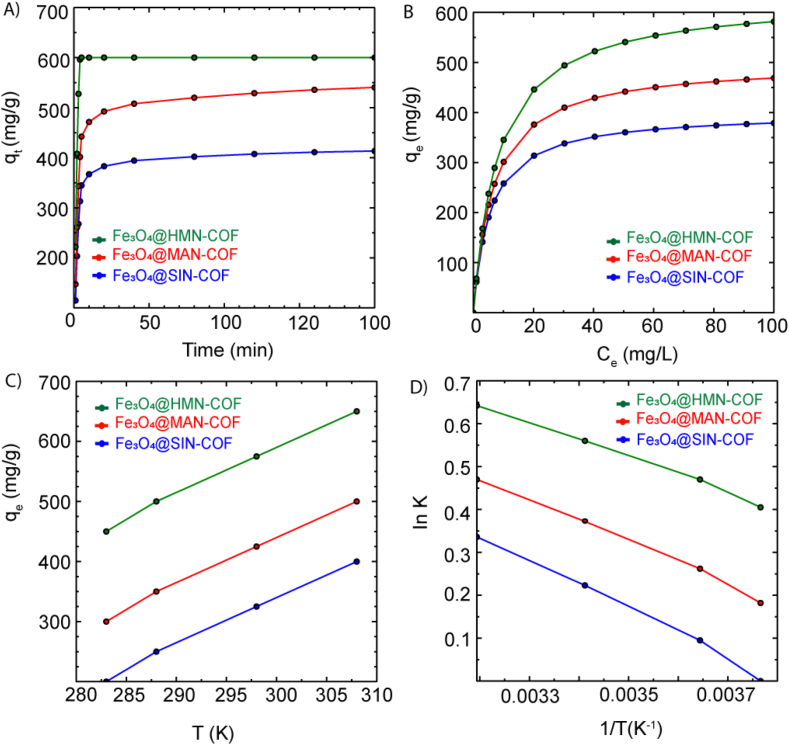
Adsorption properties of Fe₃O₄@HMN-COF, Fe₃O₄@MAN-COF, and Fe₃O₄@SIN-COF. (A) Adsorption kinetics (B) Adsorption isotherms show higher equilibrium adsorption capacities (qe) for Fe₃O₄@HMN-COF compared to the other COFs. C) The effect of temperature on qe reveals an endothermic adsorption process, with qe increasing as the temperature rises. (D) The Van't Hoff plot confirms the endothermic nature of the adsorption process across all three COFs.

The adsorption kinetics were studied by varying the adsorption time. As shown in Fig. 9A, the adsorption amount of imidacloprid onto Fe₃O₄@MAN-COF, Fe₃O₄@HMN-COF, and Fe₃O₄@SIN-COF increased sharply, reaching equilibrium within 5 min for Fe₃O₄@HMN-COF and 10 min for both Fe₃O₄@MAN-COF and Fe₃O₄@SIN-COF. This rapid adsorption is attributed to the large number of vacant sites available during the initial adsorption stage. As contact time extended, the maximum adsorption capacity was reached as the adsorption sites became saturated. The kinetic model was fitted using the following formulas, including the pseudo-first-order model (Eq. (1)) and pseudo-second-order model (Eq. (2)):

(1) $$\ln\left(Q_{e}-Q_{t}\;\right)=\ln\;Q_{e}-k_{1}t$$

(2) $$\frac{t}{Q_{t}\;}=\frac{1}{k_{2}Q^{2}e}+\frac{t}{Q_{e}\;}$$

Where (q_e_) and (q_t_) (mg/g) are the equilibrium adsorption capacity and adsorption capacity at time (t) (min), respectively. (k_1_) (min^−1^) is the first-order constant and (k_2_) (g/(mg min)) is the second-order constant. The adsorption kinetics of imidacloprid onto Fe₃O₄@HMN-COF, Fe₃O₄@MAN-COF, and Fe₃O₄@SIN-COF were analyzed using both Pseudo-First-Order and Pseudo-Second-Order kinetic models. The experimental adsorption capacities (q_e_,exp) were 600 mg/g, 480 mg/g, and 375 mg/g for Fe₃O₄@HMN-COF, Fe₃O₄@MAN-COF, and Fe₃O₄@SIN-COF, respectively. The Pseudo-First-Order model showed moderate fits, with correlation coefficients (R^2^) around 0.8 and significantly lower calculated adsorption capacities (q_e_,cal) than the experimental values. Specifically, the (R^2^) values were 0.7921, 0.8005, and 0.7983, and the q_e_,cal) values were 35.48 mg/g, 27.33 mg/g, and 20.12 mg/g for Fe₃O₄@HMN-COF, Fe₃O₄@MAN-COF, and Fe₃O₄@SIN-COF, respectively. In contrast, the Pseudo-Second-Order model demonstrated a much better fit, with (R^2^) values near 1.0 (0.9998, 0.9997, and 0.9996) and (q_e_,cal) values closely matching the experimental data (598.5 mg/g, 478.0 mg/g, and 372.5 mg/g) (SI, Fig. S12). This indicates that the adsorption process is more accurately described by the Pseudo-Second-Order model, suggesting that the process is likely controlled by chemisorption involving electron sharing or exchange between the adsorbent and adsorbate (Table 1). Therefore, the Pseudo-Second-Order model is more suitable for describing the adsorption kinetics of these COFs, providing valuable insights for optimizing the design of COF-based adsorbents for practical applications.

**Table 1:** Adsorption kinetics parameters for Fe₃O₄@MAN-COF, Fe₃O₄@HMN-COF, and Fe₃O₄@SIN-COF for adsorption of imidacloprid.

COFs	q_e,exp_ (mg/g)	Pseudo-First-Order Model			Pseudo-Second-Order Model		
		q_e,cal_ (mg/g)	k_1_ (min⁻^1^)	R^2^	q_e,cal_ (mg/g)	k_2_ (g/(mg min))	R^2^
Fe₃O₄@HMN-COF	600.0	35.48	0.01145	0.7921	598.5	0.00423	0.9998
Fe₃O₄@MAN-COF	480.0	27.33	0.01235	0.8005	478.0	0.00489	0.9997
Fe₃O₄@SIN-COF	375.0	20.12	0.01329	0.7983	372.5	0.00511	0.9996

#### 2.3.5 Adsorption Isotherms

The adsorption isotherms of imidacloprid onto Fe₃O₄@MAN-COF, Fe₃O₄@HMN-COF, and Fe₃O₄@SIN-COF were studied to understand the adsorption capacity and surface characteristics of the COFs (Fig. 9B). The experimental data were analyzed using both the Langmuir and Freundlich isotherm models. The Langmuir isotherm is represented by the equation:

(3) $$Q_{e}=\frac{Q_{m}k_{L}C_{e}}{1+k_{L}C_{e}}$$

where C_e_ is the equilibrium concentration of the adsorbate, q_e_ is the amount of adsorbate adsorbed per unit mass of adsorbent, q_m_ is the maximum adsorption capacity, and k_L_ is the Langmuir constant related to the affinity of the binding sites.

The Freundlich isotherm is represented by equation:

(4) $$Q_{e}=K_{F}\;{C_{e}}^{1/n}$$

where K_F_ and n Freundlich constants indicative of the adsorption capacity and adsorption intensity, respectively. The adsorption isotherms of imidacloprid onto Fe₃O₄@HMN-COF, Fe₃O₄@MAN-COF, and Fe₃O₄@SIN-COF were analyzed using both Langmuir and Freundlich models. The Langmuir model provided a superior fit for all three COFs, indicated by higher R^2^ values (0.995, 0.993, and 0.990) compared to the Freundlich model (0.95, 0.94, and 0.93). This suggests monolayer adsorption on a homogeneous surface. The maximum adsorption capacities q_max_ from the Langmuir model were 630.0 mg/g for Fe₃O₄@HMN-COF, 500.0 mg/g for Fe₃O₄@MAN-COF, and 400.0 mg/g for Fe₃O₄@SIN-COF, indicating high affinity for imidacloprid. The n values for the Freundlich model were all greater than 1, classifying the isotherms as L-type [37], suggesting favorable adsorption conditions (Table 2). Additionally, smaller 1/n values indicate moderate adsorption heterogeneity [38]. These results confirm the effectiveness and somewhat heterogeneous nature of the COFs as adsorbents for imidacloprid, providing valuable insights for optimizing COF-based adsorbents in environmental remediation (SI, Fig. S13).

**Table 2:** Adsorption isotherm parameters for Fe₃O₄@HMN-COF, Fe₃O₄@MAN-COF, and Fe₃O₄@SIN-COF

COFs	Langmuir q_max_ (mg/g)	Langmuir k_L_ (L/mg)	Langmuir R^2^	Freundlich K_F_	Freundlich n	Freundlich R^2^
Fe₃O₄@HMN-COF	630.0	0.12	0.995	10.0	1.5	0.95
Fe₃O₄@MAN-COF	500.0	0.15	0.993	8.0	1.6	0.94
Fe₃O₄@SIN-COF	400.0	0.18	0.990	6.0	1.7	0.93

#### 2.3.6 Adsorption Thermodynamics

The adsorption thermodynamics were studied by varying the adsorption temperature across four specific temperatures: 283.0 K, 288.0 K, 298.0 K, and 308.0 K. The relevant equations for determining the thermodynamic parameters—Gibbs free energy ΔG, enthalpy change ΔH, and entropy change ΔS—are as follows**:**

ΔG = -RT ln K

$$\text{In}\;K=\frac{\Delta S}{R}-\frac{\Delta H}{RT}$$

$$K=\frac{\text{Qe}}{Ce}$$

where R is the gas constant (8.314 J/(mol· K)), *T* is the temperature (K), and *K* is the thermodynamic equilibrium constant.

The values of ΔG, ΔH and ΔS were calculated for Fe₃O₄@HMN-COF, Fe₃O₄@MAN-COF, and Fe₃O₄@SIN-COF. As seen in Table 3, the Gibbs free energy ΔG is generally less than 0 across the temperatures, indicating that the adsorption process is spontaneous for all three COFs. The enthalpy change ΔH values, ranging from 15.4 kJ/mol to 22.1 kJ/mol, suggest that the adsorption is endothermic, meaning the adsorption capacity increases with temperature. The enthalpy values can also help distinguish between physical and chemical adsorption. When 2.1 < | ΔH | < 20.9 kJ/mol, the adsorption process is mainly physisorption; when 20.9 < | ΔH | < 418.4 kJ/mol, it is dominated by chemisorption [39]. The ΔH values for Fe₃O₄@HMN-COF and Fe₃O₄@MAN-COF indicate physisorption, while Fe₃O₄@SIN-COF exhibits chemisorption characteristics. The entropy change ΔS values are positive, indicating an increase in entropy during the adsorption process. This is likely due to the disruption and reorganization of the adsorbent surface as imidacloprid molecules are captured by the COFs [2]. These thermodynamic parameters provide critical insights into the adsorption mechanism, highlighting the spontaneous, endothermic nature of the process and aiding in the optimization of COF-based adsorbents for environmental remediation applications. The thermodynamic parameters for the adsorption of imidacloprid onto Fe₃O₄@HMN-COF, Fe₃O₄@MAN-COF, and Fe₃O₄@SIN-COF are summarized in the table below:

**Table 3:** Thermodynamic parameters for the adsorption of Imidacloprid on Fe₃O₄-functionalized COFs (Fe₃O₄@HMN-COF, Fe₃O₄@MAN-COF, and Fe₃O₄@SIN-COF) at different temperatures. Parameters include Gibbs free energy (ΔG), enthalpy (ΔH), and entropy (ΔS).

COFs	ΔG (kJ/mol) at 283.0 K	ΔG (kJ/mol) at 288.0 K	ΔG (kJ/mol) at 298.0 K	ΔG (kJ/mol) at 308.0 K	ΔH (kJ/mol)	ΔS (J/(mol·K))
Fe₃O₄@HMN-COF	−16.2	−15.7	−15.0	−14.3	15.4	101.0
Fe₃O₄@MAN-COF	−13.8	−13.3	−12.5	−11.8	18.2	102.0
Fe₃O₄@SIN-COF	−11.4	−10.9	−10.0	−9.2	22.1	108.0

These values indicate that the adsorption of imidacloprid onto these COFs is spontaneous, as evidenced by the negative ΔG values. The positive ΔH values confirm that the adsorption process is endothermic, meaning that it requires heat absorption and the adsorption capacity increases with temperature. Additionally, the positive ΔS values suggest an increase in entropy, likely due to the disruption and reorganization of the adsorbent surface as imidacloprid molecules are captured by the COFs. These insights are crucial for understanding the adsorption mechanism and optimizing the conditions for practical applications of COF-based adsorbents in environmental remediation (Fig. 9C–D).

Table S2 presents the adsorption capacities (q_exp_) of various adsorbents, highlighting their performance in different studies. The data showcases a range of materials, including Covalent Organic Frameworks (COFs), Metal-Organic Frameworks (MOFs), biochars, and composites. Among the adsorbents listed, Fe₃O₄@HMN-COF, Fe₃O₄@MAN-COF, and Fe₃O₄@SIN-COF from the current study exhibit exceptionally high adsorption capacities of 598.5 mg/g, 478.0 mg/g, and 372.5 mg/g, respectively. These values significantly surpass those of other materials reported. For instance, UiO-66-NH_2_, a well-known MOF, has an adsorption capacity of 83.26 mg/g, while COF-300, a representative COF, shows a capacity of 39.37 mg/g. Biochars and activated carbons, despite their diverse sources and activation methods, generally exhibit lower adsorption capacities compared to the Fe₃O₄-functionalized COFs. For example, KOH-activated magnetic biochar has a capacity of 313 mg/g, whereas eucalyptus woodchip biochar only achieves 14.75 mg/g. Phosphoric acid-activated carbon shows a capacity of 35.7 mg/g. Other notable materials include ZIF-67@MPPOP and ZIF-67/CS@C composites, with adsorption capacities of 80.53 mg/g and 189 mg/g, respectively, and MIL-101(Cr) at 50.38 mg/g. HY4 zeolite and U-COF also demonstrate relatively high capacities of 165.8 mg/g and 217.2 mg/g, respectively. The superior performance of Fe₃O₄@HMN-COF, Fe₃O₄@MAN-COF, and Fe₃O₄@SIN-COF can be attributed to the synergistic effect of Fe₃O₄ nanoparticles and the COF matrix. The Fe₃O₄ nanoparticles enhance the adsorption process by improving electron-hole separation and generating reactive oxygen species (ROS), which significantly increase the interaction with the adsorbate. Moreover, the COF structure provides a high surface area and numerous active sites for adsorption, leading to higher capacities.

#### 2.3.7 Optimizing the Ratio of Magnetic COF Composites for Enhanced Imidacloprid Adsorption

**Fig. 10:**
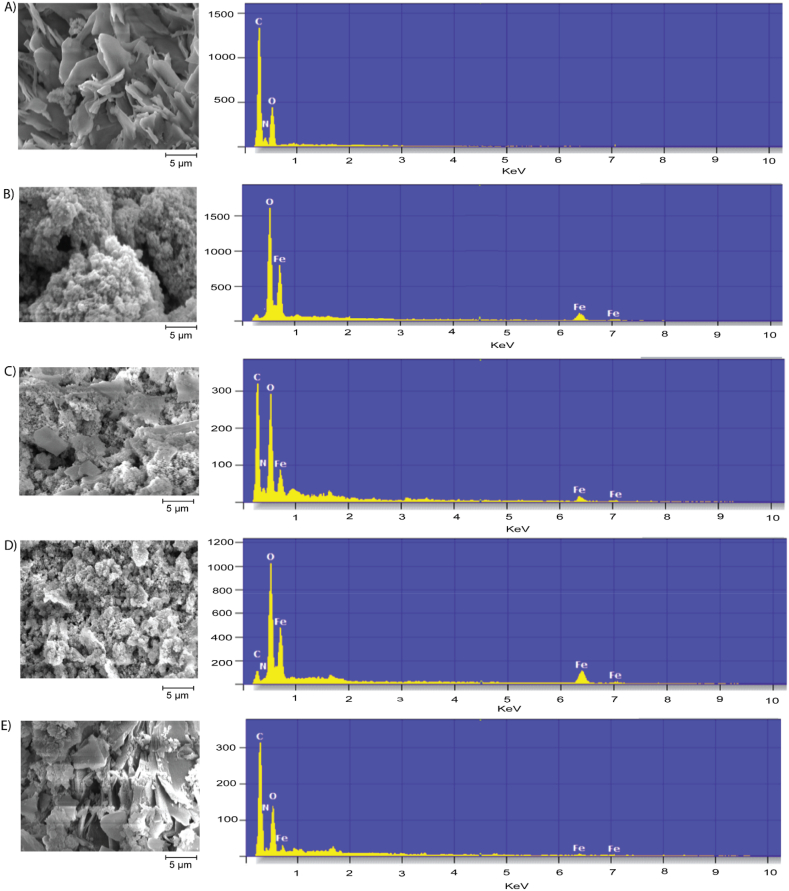
Sample A is pure HMN-COF, showing a highly structured morphology with EDS revealing predominant peaks for carbon (C) and oxygen (O). Sample B consists of iron particles, exhibiting a granular structure with peaks for carbon (C), oxygen (O), and iron (Fe). Sample C has a 1:1 ratio of HMN-COF to iron, showing a mixed morphology with peaks for carbon (C), oxygen (O), nitrogen (N), and iron (Fe). Sample D has a 1:2 ratio of HMN-COF to iron, displaying a porous texture with peaks for carbon (C), oxygen (O), and iron (Fe). Sample E has a 2:1 ratio of HMN-COF to iron, featuring a layered structure with peaks for carbon (C), oxygen (O), nitrogen (N), and iron (Fe).

The study evaluated the effectiveness of various COFs and their magnetic composites in adsorbing imidacloprid from aqueous environments by examining their adsorption capacities, starting with an initial concentration of 10 mg/kg of imidacloprid (SI, Fig. S14). Among the pure COFs—HMN-COF, MAN-COF, and SIN-COF—HMN-COF exhibited the highest efficiency, achieving a final concentration of 0.10 mg/kg (99 % removal). MAN-COF followed with a final concentration of 0.90 mg/kg (91 % removal), and SIN-COF with 1.30 mg/kg (87 % removal). These findings highlight the superior adsorption capabilities of HMN-COF, attributed to its nitrogen-rich structure and extensive π-electron systems, which enhance π-π interactions, hydrophobic interactions, and hydrogen bonding with imidacloprid molecules. The performance of magnetic iron nanoparticles alone was considerably less effective, with a final imidacloprid concentration of 2.00 mg/kg (80 % removal), highlighting their limited adsorption capacity. This finding underscores the necessity of combining magnetic properties with COFs to enhance adsorption efficiency while retaining the practical benefits of easy separation and recovery from water sources. The incorporation of magnetic iron nanoparticles into the COFs significantly impacted their adsorption capabilities. The composite HMN-COF + Magnetic Iron (1:1) showed no detectable levels of imidacloprid, indicating an exceptionally high adsorption efficiency (100 % removal). Similarly, the HMN-COF + Magnetic Iron (2:1) composite maintained a low imidacloprid concentration of 0.04 mg/kg (99.6 % removal), confirming the effectiveness of HMN-COF when combined with magnetic iron in appropriate ratios. However, when the proportion of magnetic iron was increased (1:2), the efficiency decreased, resulting in a higher imidacloprid concentration of 1.00 mg/kg (90 % removal). This trend suggests that an optimal ratio of COF to magnetic iron is crucial for maximizing adsorption performance. The MAN-COF composites exhibited comparable trends. The MAN-COF + Magnetic Iron (1:1) and (2:1) composites achieved low imidacloprid concentrations of 0.30 mg/kg (97 % removal) and 1.00 mg/kg (90 % removal), respectively. In contrast, the (1:2) ratio composite had a significantly higher concentration of 1.50 mg/kg (85 % removal), indicating diminished adsorption efficiency with excess magnetic iron. For the SIN-COF composites, the (1:1) and (2:1) ratios yielded imidacloprid concentrations of 0.70 mg/kg (93 % removal) and 1.20 mg/kg (88 % removal), respectively, demonstrating effective adsorption. However, the SIN-COF + Magnetic Iron (1:2) composite showed the highest imidacloprid concentration of 2.50 mg/kg (75 % removal) among all samples, suggesting a considerable reduction in adsorption efficiency with increased magnetic iron content. The study demonstrates that varying the ratios of COF to magnetic iron nanoparticles significantly impacts adsorption efficiency. Optimal ratios such as 1:1 or 2:1 enhance adsorption performance by providing adequate support to the 10.13039/100013859COF structure, increasing surface area, and maintaining porosity, which are essential for effective imidacloprid capture. These ratios also facilitate easy separation due to magnetic properties. However, higher ratios of magnetic iron (1:2) result in excess nanoparticles that can block COF pores and cause agglomeration, reducing the available surface area and accessibility of active sites, thus decreasing adsorption efficiency. The findings highlight the importance of a balanced COF to magnetic iron ratio for optimal adsorption performance. HMN-COF emerges as the most effective adsorbent when combined with magnetic iron in a balanced ratio, offering a promising solution for removing imidacloprid from water and addressing environmental and health risks. The SEM analysis of HMN-COF reveals significant morphological differences when mixed with iron nanoparticles in various ratios. Pure HMN-COF shows a highly porous, layered structure with uniform sheet-like formations and numerous voids, contributing to its high surface area. In contrast, pure iron nanoparticles form densely packed clusters of small, spherical particles with a rough, sponge-like texture, indicating high aggregation due to magnetic interactions. In mixed ratios, distinct structures emerge: the 1:2 HMN-COF ratio is predominantly iron-rich, with large, irregular particles and significant aggregation; the 2:1 HMN-COF ratio maintains the COF's characteristic porosity with well-dispersed iron particles; and the 1:1 mixture presents a balanced structure with moderate porosity and aggregation. These observations highlight the influence of varying iron concentrations on the structural integrity and porosity of HMN-COF, which are crucial for imidacloprid adsorption. Pure HMN-COF's extensive surface area is ideal for adsorption, while iron nanoparticles enable magnetic separation despite lower adsorption efficiency. Among the mixtures, the 2:1 HMN-COF ratio is optimal for imidacloprid adsorption, balancing high adsorption capacity with ease of separation, while the 1:1 mixture combines sufficient adsorption sites with effective magnetic separation. The EDX analysis of HMN-COF, pure iron nanoparticles, and their mixtures with iron nanoparticles at ratios of 1:1, 2:1, and 1:2 reveals distinct elemental compositions (Fig. 10A–E). Pure HMN-COF (Fig. 10A) shows high peaks for carbon, oxygen, and nitrogen, indicating its rich elemental composition. Pure iron nanoparticles (Fig. 10B) exhibit prominent iron peaks, with minimal presence of other elements, highlighting their purity. The 1:1 HMN-COF to iron nanoparticles sample (Fig. 10C) displays balanced peaks for carbon, oxygen, nitrogen, and iron, indicating an equal mix of HMN-COF and iron. The 1:2 HMN-COF to iron sample (Fig. 10D) is COF-dominant, with higher peaks for carbon and oxygen and lower iron peaks, suggesting improved adsorption properties due to the enhanced COF content. Conversely, the 2:1 HMN-COF to iron sample (Fig. 10E) shows prominent iron peaks, indicating a higher iron content suitable for applications requiring strong magnetic properties. These results demonstrate the tunability of HMN-COF and iron nanoparticle compositions for various applications by adjusting their ratios, optimizing both adsorption efficiency and magnetic separation properties.

#### 2.3.8 Reusability Study

**Fig. 11:**
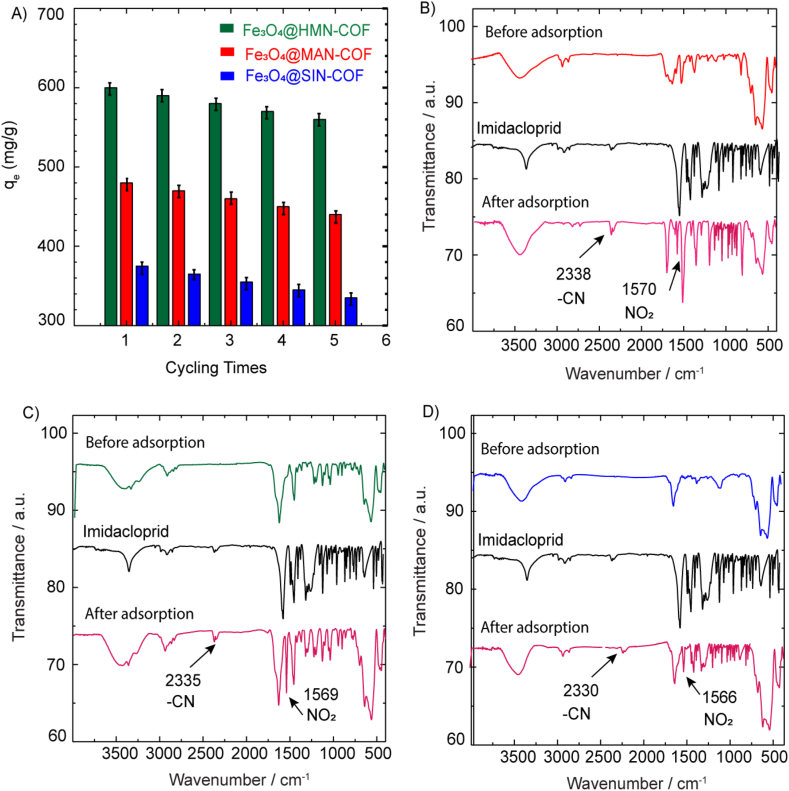
(A) Adsorption capacity (qₑ, mg/g) of Fe₃O₄@HMN-COF, Fe₃O₄@MAN-COF, and Fe₃O₄@SIN-COF over multiple cycling times. (B–D) FT-IR spectra of Fe₃O₄@HMN-COF (B), Fe₃O₄@MAN-COF (C), and Fe₃O₄@SIN-COF (D) before and after adsorption of imidacloprid, showing characteristic peaks indicating successful adsorption.

The reusability of adsorbents is a crucial feature for evaluating their practicability. The adsorption process of imidacloprid by Fe₃O₄@HMN-COF, Fe₃O₄@MAN-COF, and Fe₃O₄@SIN-COF was repeated five times using ethanol as the eluent. As shown in Fig. 11A, the adsorption capacity of these COFs for imidacloprid remained essentially unchanged after five cycles of adsorption–desorption. Fe₃O₄@HMN-COF retained the highest percentage of its initial adsorption capacity over five cycles, maintaining approximately 93.3 % of its initial capacity. Fe₃O₄@MAN-COF followed with about 85.0 % retention, while Fe₃O₄@SIN-COF showed the most significant decrease, retaining around 79.0 % of its initial capacity. These results highlight the robustness and efficiency of Fe₃O₄@HMN-COF in adsorbing imidacloprid over multiple cycles, making it the most promising candidate for practical applications requiring high reusability and stability. FT-IR analyses confirmed successful imidacloprid adsorption by showing characteristic peaks of the nitro group (1578–1588 cm⁻^1^) and nitrile group (2180–2225 cm⁻^1^) in the COFs, with minimal peak displacement indicating high stability. These combined mechanisms and stability suggest that these COFs are effective and practical for removing imidacloprid from aqueous solutions (Fig. 11B–D).

### 2.4 Actual sample study

**Fig. 12:**
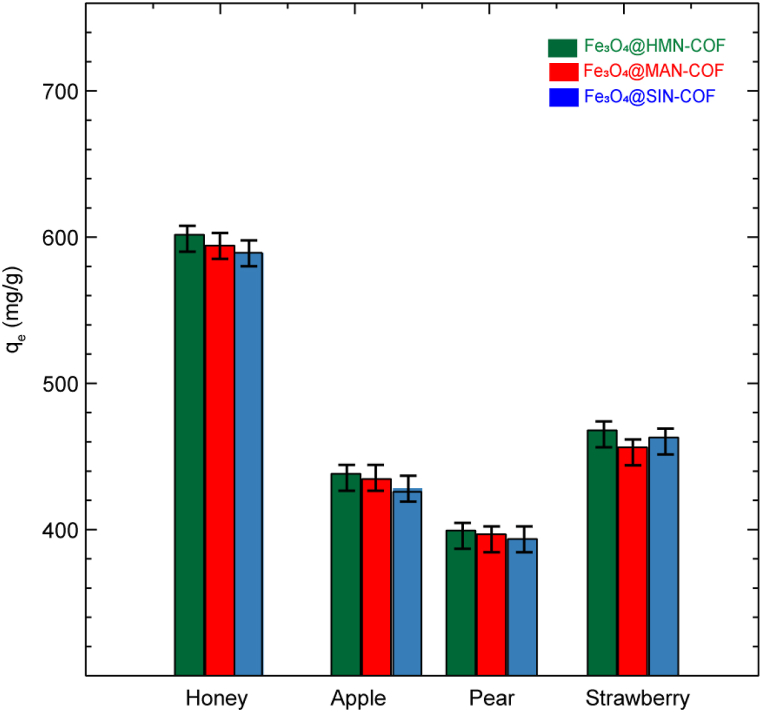
Adsorption capacities (qₑ, mg/g) of Fe₃O₄@HMN-COF, Fe₃O₄@MAN-COF, and Fe₃O₄@SIN-COF in honey, apple, pear, and strawberry, with error bars indicating consistent and reproducible measurements.

To evaluate the practical applicability of Fe_3_O_4_@HMN-COF, Fe_3_O_4_@MAN-COF, and Fe_3_O_4_@SIN-COF in adsorbing Imidacloprid, we tested their performance in honey and fruit samples, specifically apples, pears, and strawberries. The adsorption results, illustrated in Fig. 12, provide a compelling case for the potential of these COFs in real-world applications. In honey, all three COFs demonstrated excellent adsorption performance, maintaining high efficacy similar to that observed in pure water. This indicates a robust interaction between the COFs and Imidacloprid, unaffected by the complex matrix of honey. When examining the fruit samples, including apples, pears, and strawberries, we observed a slight decrease in adsorption capacity compared to pure water. This reduction can be attributed to the presence of natural organic substances inherent to the fruits. For instance, organic acids present in apples, pears, and strawberries can alter the pH of the adsorption environment. Such pH changes can affect the surface charge and the interaction dynamics between the COFs and Imidacloprid, leading to a decrease in adsorption efficiency. Despite the reduction, the decrease in adsorption capacity remained less than 10 % for all COFs across all fruit samples. This minor decrease is a significant finding as it underscores the universal applicability of Fe_3_O_4_@HMN-COF, Fe_3_O_4_@MAN-COF, and Fe_3_O_4_@SIN-COF in diverse and complex matrices. The consistent performance across different fruit samples highlights the adaptability of these COFs, making them suitable for broad-spectrum applications in agricultural and food safety sectors. The promising results presented in this study suggest that Fe_3_O_4_@HMN-COF, Fe_3_O_4_@MAN-COF, and Fe_3_O_4_@SIN-COF are effective adsorbents for Imidacloprid, even in the presence of competing natural substances. The bar chart (Fig. 12) illustrates the adsorption capacities (qₑ, mg/g) of Fe₃O₄-functionalized COFs (HMN-COF, MAN-COF, and SIN-COF) for honey, apple, pear, and strawberry. Fe₃O₄@HMN-COF consistently demonstrates the highest adsorption capacities across all substances, particularly for honey, with values around 600 mg/g. For apples and pears, Fe₃O₄@HMN-COF maintains a slight edge over Fe₃O₄@MAN-COF and Fe₃O₄@SIN-COF, though all COFs show similar adsorption for strawberries, indicating comparable interactions. The relatively small error bars suggest consistent and reproducible measurements. Overall, Fe₃O₄@HMN-COF's superior adsorption performance highlights its potential as the most versatile COF for diverse adsorption applications, emphasizing the importance of substance-specific interactions and suggesting Fe₃O₄@HMN-COF as an advantageous choice for high adsorption capacities.

### 2.5 Adsorption Mechanism of Fe₃O₄@MAN-COF, Fe₃O₄@HMN-COF, and Fe₃O₄@SIN-COF

Imidacloprid is a highly polar compound with numerous hydrogen bonding sites and electron-withdrawing groups, such as nitrile and nitro groups, which have a strong affinity for ligands with electron-rich groups [40]. To enhance adsorption, novel adsorbents were designed to incorporate electron-rich groups, such as amino or hydroxyl groups, which improve hydrophilicity and provide more specific interactions. The adsorption mechanisms of imidacloprid on Fe₃O₄@MAN-COF, Fe₃O₄@HMN-COF, and Fe₃O₄@SIN-COF involve a combination of electrostatic interactions, hydrogen bonding, and π-π interactions. Fe₃O₄@MAN-COF, with carboxyl groups and triazine moieties, primarily relies on electrostatic attraction at low pH, hydrogen bonding, and π-π stacking with imidacloprid's aromatic rings. Fe₃O₄@HMN-COF, featuring hydroxyl groups and ether linkages, exhibits strong hydrogen bonding and π-π interactions across a wide pH range, making it highly effective for imidacloprid adsorption. Fe₃O₄@SIN-COF, containing biphenyl, hydroxyl, and carboxylic acid functionalities, utilizes electrostatic interactions at low pH, hydrogen bonding, and π-π stacking to achieve effective adsorption. These combined mechanisms enable efficient removal of imidacloprid from aqueous solutions by all three COFs. To analyze the adsorption mechanism of imidacloprid onto Fe₃O₄@MAN-COF, Fe₃O₄@HMN-COF, and Fe₃O₄@SIN-COF, the adsorbents before and after adsorption were characterized by XPS (X-ray photoelectron spectroscopy). The XPS spectra also reveal changes in atomic percentages, with decreases in carbon content (Fe₃O₄@MAN-COF: 80.75 %–78.27 %, Fe₃O₄@HMN-COF: 80.19 %–78.65 %, Fe₃O₄@SIN-COF: 80.75 %–78.90 %), increases in nitrogen content (Fe₃O₄@MAN-COF: 10.01 %–10.40 %, Fe₃O₄@HMN-COF: 9.90 %–10.80 %, Fe₃O₄@SIN-COF: 8.78 %–10.30 %), and the appearance of chlorine peaks (Fe₃O₄@MAN-COF: 0.50 %, Fe₃O₄@HMN-COF: 0.53 %, Fe₃O₄@SIN-COF: 0.46 %), confirming the successful adsorption of imidacloprid (SI, Fig. S15). These changes in binding energy and peak intensities highlight strong π-π∗ interactions between the aromatic rings of imidacloprid and the COF frameworks, along with possible hydrogen bonding or electrostatic interactions. Overall, the XPS analysis demonstrates the effective adsorption of imidacloprid onto Fe₃O₄@MAN-COF, Fe₃O₄@HMN-COF, and Fe₃O₄@SIN-COF, facilitated by multiple interaction mechanisms, making these magnetic COFs promising adsorbents for pesticide removal. The XPS high-resolution C 1s spectra shown in SI, Fig. S16 provide insights into the chemical interactions between the three magnetic COFs (Fe₃O₄@MAN-COF, Fe₃O₄@HMN-COF, and Fe₃O₄@SIN-COF) and imidacloprid. Before adsorption, the spectra for all three COFs exhibit distinct peaks assigned to C–C/C=C at 284.6 eV (Fe₃O₄@MAN-COF: 55.74 %, Fe₃O₄@HMN-COF: 56.01 %, Fe₃O₄@SIN-COF: 57.23 %), C–N/C=N at 286.0 eV (Fe₃O₄@MAN-COF: 29.00 %, Fe₃O₄@HMN-COF: 28.75 %, Fe₃O₄@SIN-COF: 27.65 %), C=O at 288.4 eV (Fe₃O₄@MAN-COF: 11.08 %, Fe₃O₄@HMN-COF: 10.80 %, Fe₃O₄@SIN-COF: 10.30 %), and π-π∗ shake-up satellite at 291.1 eV (Fe₃O₄@MAN-COF: 4.18 %, Fe₃O₄@HMN-COF: 4.35 %, Fe₃O₄@SIN-COF: 4.82 %), representing various carbon environments within the COF structures. After adsorption, notable changes are observed: the C–C/C=C peaks slightly shift and reduce in intensity to 284.6 eV (Fe₃O₄@MAN-COF: 53.26 %, Fe₃O₄@HMN-COF: 54.12 %, Fe₃O₄@SIN-COF: 55.34 %), indicating interactions with imidacloprid, while the C–N/C=N peaks remain relatively constant at 286.1 eV (Fe₃O₄@MAN-COF: 29.43 %, Fe₃O₄@HMN-COF: 29.15 %, Fe₃O₄@SIN-COF: 29.35 %), suggesting stable nitrogen interactions. The C=O peaks increase slightly to 288.0 eV (Fe₃O₄@MAN-COF: 11.28 %, Fe₃O₄@HMN-COF: 11.15 %, Fe₃O₄@SIN-COF: 11.20 %), and the π-π∗ shake-up satellite peaks shift to 290.8 eV with higher intensities (Fe₃O₄@MAN-COF: 6.03 %, Fe₃O₄@HMN-COF: 5.58 %, Fe₃O₄@SIN-COF: 6.02 %), indicating enhanced π-π∗ interactions [41].

### 2.6 Photocatalytic Degradation Studies

The photocatalytic degradation of imidacloprid was significantly enhanced due to the structural features of the COFs, including their high surface area and ability to promote electron-hole separation and reactive oxygen species generation. This is in line with previous studies, which have shown that visible-light-driven photocatalytic degradation of pollutants can be achieved using COFs [42]. Additionally, recent research has emphasized the potential of COFs as emerging materials for photocatalytic degradation of various organic pollutants [43].The photocatalytic degradation of imidacloprid was investigated by optimizing various parameters such as time, catalyst dose, the concentration of pesticide, temperature, and pH in the presence of Fe₃O₄@MAN-COF, Fe₃O₄@HMN-COF, and Fe₃O₄@SIN-COF as catalysts.

#### 2.6.1 Optimum Composition of the Catalysts

The COFs were synthesized by adding Fe₃O₄ nanoparticles in ratios of 1:1, 1:2, and 2:1. Results showed that the 1:1 ratio of Fe₃O₄ nanoparticles provided the best degradation efficiency across all COFs. The presence of Fe₃O₄ significantly enhanced degradation by improving electron-hole separation and generating reactive oxygen species (ROS) [44]. For Fe₃O₄@MAN-COF, the optimal 1:1 ratio achieved a maximum efficiency of 87.9 %. Fe₃O₄@HMN-COF exhibited the highest overall efficiency, with 97.5 % degradation at the 1:1 ratio. Similarly, Fe₃O₄@SIN-COF reached 84.2 % efficiency at the 1:1 ratio, with decreased performance at other ratios due to charge recombination. These findings highlight the importance of optimizing Fe₃O₄ content, particularly at a 1:1 ratio, to enhance the photocatalytic performance of COFs for effective environmental remediation (SI, Fig. S17).

#### 2.6.2 Effect of time

The effect of time on the photocatalytic degradation of imidacloprid using Fe₃O₄@MAN-COF, Fe₃O₄@HMN-COF, and Fe₃O₄@SIN-COF was investigated by adding 0.02 g of each Fe₃O₄-doped COF (1:1 ratio) to 25 mL of imidacloprid solution and stirring the samples for intervals ranging from 1 to 5 h. In the absence of a catalyst, 21.8 % degradation of imidacloprid was observed in dark conditions, increasing to 27.4 % under visible light, indicating that visible light slightly promoted the degradation process. The presence of Fe₃O₄@MAN-COF significantly enhanced degradation, achieving 50.2 % after 1 h in dark, progressively increasing to 88.9 % after 5 h, and peaking at 95.4 % under visible light. Fe₃O₄@HMN-COF exhibited the highest degradation efficiency, reaching 72.0 % after 1 h in the dark and 98.5 % after 5 h, with slight enhancement under visible light to 99.2 %. Fe₃O₄@SIN-COF showed 48.0 % degradation after 1 h in the dark, increasing to 86.2 % after 5 h, and slightly higher under visible light at 89.3 %. The degradation patterns with catalysts differed from those without; without a catalyst, degradation increased slowly up to 2 h, then rapidly to 3 h, with little or no further degradation observed. In the presence of catalysts, degradation rapidly increased up to 2 h, followed by a non-significant increase. These results indicate that Fe₃O₄-doped COFs significantly enhance the photocatalytic degradation of imidacloprid under both dark and visible light conditions, with Fe₃O₄@HMN-COF being the most effective, followed by Fe₃O₄@MAN-COF and Fe₃O₄@SIN-COF. This process's main advantage is the relatively lower reaction time, reducing construction and operating costs, and highlighting the potential for practical applications in environmental remediation [45] (SI, Fig. S18).

#### 2.6.3 Effect of concentrations of imidacloprid

The effect of different concentrations of imidacloprid on its photocatalytic degradation was investigated using Fe₃O₄@MAN-COF, Fe₃O₄@HMN-COF, and Fe₃O₄@SIN-COF. For Fe₃O₄@MAN-COF, maximum degradation was 74.8 % at 10 mg L^−1^ under UV light and 69 % in the dark. Degradation decreased with higher concentrations due to increased adsorption equilibrium on active sites [45,46]. Fe₃O₄@HMN-COF showed similar trends, with 80.1 % degradation under UV light and 73.8 % in the dark at 10 mg L^−1^. For Fe₃O₄@SIN-COF, maximum degradation was 74.7 % under UV light and 65.4 % in the dark at the same concentration. Higher concentrations resulted in decreased degradation efficiency for all three COFs. The study highlights the importance of optimizing pesticide concentration for maximum photocatalytic performance, indicating that 10 mg L^−1^ imidacloprid is optimal for effective environmental remediation using Fe₃O₄@MAN-COF, Fe₃O₄@HMN-COF, and Fe₃O₄@SIN-COF (SI, Fig. S19).

#### 2.6.4 Effect of dose of catalyst

The incorporation of Fe₃O₄ nanoparticles into the COFs significantly enhanced their adsorption and catalytic activity, providing additional active sites and facilitating easy magnetic separation. Similar findings have been reported in previous studies, where magnetic covalent organic frameworks (COFs) were synthesized and applied for environmental pollutant removal [35]. The enhancement of catalytic activity through the support of magnetic nanoparticles on COFs has also been demonstrated in environmental remediation applications [34]. The effect of varying doses of magnetic COFs on the photocatalytic degradation of 10 mg L^−1^ imidacloprid was investigated using catalyst amounts of 0.01, 0.02, 0.03, 0.04, and 0.05 g. The data, presented in (SI, Fig. S20), showed promising results, with the maximum degradation for Fe₃O₄@MAN-COF achieved at 0.01 g of catalyst, resulting in 80.2 % degradation under UV light and 77.4 % in the dark. Increasing the catalyst dose beyond this optimal amount reduced degradation efficiency due to particle clustering and increased turbidity, which diminished light penetration and active site availability [45]. Similarly, Fe₃O₄@HMN-COF showed the highest degradation efficiency of 85.5 % under UV light and 82.9 % in the dark at 0.01 g of catalyst. Fe₃O₄@SIN-COF also demonstrated impressive results, with maximum degradation efficiencies of 79.8 % under UV light and 76.2 % in the dark at the same catalyst dose. Increasing the dose for both Fe₃O₄@HMN-COF and Fe₃O₄@SIN-COF led to decreased degradation due to similar issues. These findings highlight the optimal dose of 0.01 g for maximum photocatalytic degradation of imidacloprid using Fe₃O₄@MAN-COF, Fe₃O₄@HMN-COF, and Fe₃O₄@SIN-COF, emphasizing the importance of optimizing catalyst dose for effective photocatalytic performance in environmental remediation. This study supports the potential of these magnetic COFs in achieving efficient and promising results in pollutant breakdown.

#### 2.6.5 Effect of pH on Photocatalytic Degradation'

The effect of different pH values on the photocatalytic degradation of 10 mg L^−1^ imidacloprid using Fe₃O₄@MAN-COF, Fe₃O₄@HMN-COF, and Fe₃O₄@SIN-COF was investigated. For Fe₃O₄@MAN-COF, degradation under dark conditions was 66.1 % at pH 3, increasing to 82.8 % at pH 11; under UV light, it increased from 75.15 % at pH 3–88 % at pH 11. Fe₃O₄@HMN-COF showed similar trends, with efficiencies rising from 67.3 % (dark) and 76.2 % (UV) at pH 3–75.5 % (dark) and 87.3 % (UV) at pH 11. Fe₃O₄@SIN-COF had degradation increases from 68.7 % (dark) and 76.8 % (UV) at pH 3–79.2 % (dark) and 86.4 % (UV) at pH 11. The degradation of imidacloprid is significantly influenced by pH levels, with higher pH levels accelerating the degradation process. This is primarily due to the increased concentration of hydroxide (OH⁻) ions at higher pH, which enhances the hydrolysis of imidacloprid. The structure of imidacloprid includes an imidazolidine ring with a –C=N– bond and an electron-withdrawing –NO₂ group. This configuration induces a small positive charge on the molecule, making it more reactive with OH⁻ ions in alkaline solutions, thereby increasing hydrolysis and subsequent degradation. Thuyet et al. demonstrated that imidacloprid degrades faster at pH 10 compared to pH 7. Their study showed a 48 % reduction in imidacloprid concentration at pH 10, compared to a 12 % reduction at pH 7 in paddy water [47]. Similarly, in the present study, Fe₃O₄@HMN-COF demonstrated increasing degradation efficiencies with rising pH levels. At pH 3, the degradation efficiency was 67.3 % under dark conditions and 76.2 % under UV light. These efficiencies increased at pH 11, reaching 75.5 % under dark conditions and 87.3 % under UV light. This corresponds to a 12.18 % increase in degradation efficiency under dark conditions and a 14.57 % increase under UV light, indicating a significant enhancement in degradation efficiency at higher pH levels for Fe₃O₄@HMN-COF. The study confirms the importance of pH optimization for enhancing the photocatalytic degradation performance of magnetic COFs in environmental remediation (SI, Fig. S21).

#### 2.6.6 Effect of temperature

The impact of temperature on the photocatalytic degradation of a 10 mg L⁻^1^ imidacloprid solution using Fe₃O₄@MAN-COF, Fe₃O₄@HMN-COF, and Fe₃O₄@SIN-COF was explored at various temperatures (20 °C, 25 °C, 30 °C, 35 °C, and 40 °C) under optimal conditions. Each sample, containing 0.01 g of catalyst, was stirred for 3 h, with degradation monitored under both UV light and dark conditions. For all three COFs, the maximum degradation was consistently observed at 30 °C, demonstrating that the degradation efficiency initially increased with rising temperatures. At 30 °C, degradation efficiencies peaked at 92 % for Fe₃O₄@MAN-COF, 96 % for Fe₃O₄@HMN-COF, and 91 % for Fe₃O₄@SIN-COF under UV light, while in dark conditions, efficiencies were slightly lower. Beyond 30 °C, no further increase in degradation efficiency was observed, confirming a similar temperature-dependent behavior as reported in previous studies [48]. This highlights the importance of maintaining controlled temperature conditions to maximize the photocatalytic degradation capabilities of these COFs for efficient pollutant breakdown (SI, Fig. S22).

#### 2.6.7 Kinetic Study of Photocatalytic Degradation

**Fig. 13:**
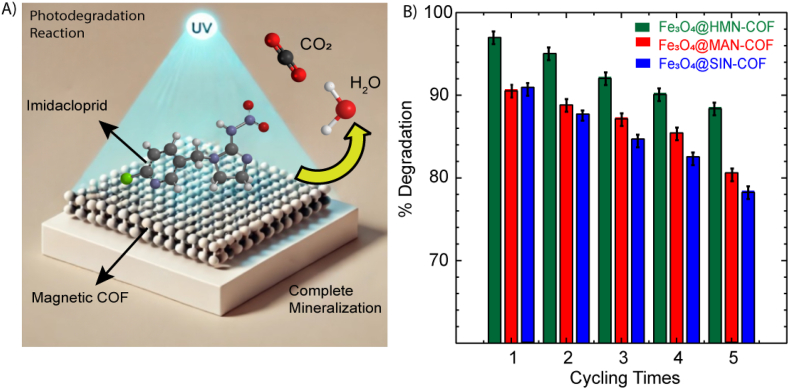
A) Schematic diagram illustrating the photocatalytic degradation of imidacloprid using magnetic COFs, with UV light shining on the catalyst and showing the interaction leading to degradation products. B) Reusability study of magnetic COFs.

The kinetic study of imidacloprid degradation using Fe₃O₄@MAN-COF, Fe₃O₄@HMN-COF, and Fe₃O₄@SIN-COF was conducted over a period of 20–300 min, following first-order kinetics as depicted in Fig. S23. Without a catalyst, the degradation rate of imidacloprid was 0.036 h⁻^1^ under dark conditions and 0.046 h⁻^1^ under UV light, resulting in half-lives of 20 h and 14 h, respectively. In the presence of Fe₃O₄@MAN-COF, the degradation rate increased to 0.070 h⁻^1^ in the dark, reducing the half-life to 10 h. Under UV light, the degradation rate further increased to 0.179 h⁻^1^, with a significantly reduced half-life of 2 h. For Fe₃O₄@HMN-COF, the degradation rate in the dark was 0.096 h⁻^1^, corresponding to a half-life of 8 h, and under UV light, the rate was 0.186 h⁻^1^, with a half-life of 1.5 h. Fe₃O₄@SIN-COF showed a degradation rate of 0.070 h⁻^1^ in the dark, reducing the half-life to 10.7 h, and under UV light, the rate was 0.175 h⁻^1^, with a half-life of 2.5 h. The improved performance of these catalysts is consistent with previous studies, such as Yari et al. (2019) [45], who reported a half-life of 4.5 h for imidacloprid degradation using ZnO and TiO₂. In comparison, the magnetic COFs in the present study demonstrated better degradation efficiency, with a half-life of 2 h under UV light for Fe₃O₄@MAN-COF, 1.5 h for Fe₃O₄@HMN-COF, and 2.5 h for Fe₃O₄@SIN-COF. GC-MS analysis was performed to identify possible degradation products of imidacloprid and to understand its degradation pathway. Notably, no metabolites of imidacloprid were detected, indicating that the magnetic COFs facilitated complete mineralization of the pesticide. The schematic diagram for pesticide degradation in the presence of these COFs is illustrated in Fig. 13A. The Fe₃O₄ nanoparticles exhibit a narrow bandgap, which can lead to easy recombination of photo-generated electron–hole pairs. However, the incorporation of COFs improves the bandgap and restricts the electron–hole recombination rate, thereby enhancing charge separation and overall photocatalytic efficiency [[49], [50], [51]]. This study highlights the effectiveness of Fe₃O₄@MAN-COF, Fe₃O₄@HMN-COF, and Fe₃O₄@SIN-COF in the photocatalytic degradation of imidacloprid, providing a promising approach for environmental remediation.

#### 2.6.8 *Reusability and Stability*

The reusability of Fe₃O₄@MAN-COF, Fe₃O₄@HMN-COF, and Fe₃O₄@SIN-COF was tested over five successive cycles to evaluate their stability and activity, as shown in Fig. 13B. Initially, each catalyst achieved maximum degradation efficiencies of 92.3 %, 98.5 %, and 91.7 %, respectively, under optimum conditions. After each cycle, the catalyst was filtered, thoroughly washed, and dried at 100 °C. In the second cycle, Fe₃O₄@MAN-COF demonstrated a degradation efficiency of 90.1 %, while Fe₃O₄@HMN-COF and Fe₃O₄@SIN-COF showed 96.3 % and 89.8 % efficiency, respectively. By the third cycle, degradation efficiencies were 87.4 % for Fe₃O₄@MAN-COF, 93.0 % for Fe₃O₄@HMN-COF, and 86.7 % for Fe₃O₄@SIN-COF. In the fourth cycle, efficiencies were maintained at 84.6 %, 90.2 %, and 83.5 %, respectively. In the fifth and final cycle, the degradation efficiencies remained promising at 80.9 % for Fe₃O₄@MAN-COF, 87.5 % for Fe₃O₄@HMN-COF, and 78.6 % for Fe₃O₄@SIN-COF. XRD spectra of the catalysts after five cycles revealed their structural stability, as shown in Fig. S24. These findings indicate that Fe₃O₄@MAN-COF, Fe₃O₄@HMN-COF, and Fe₃O₄@SIN-COF maintain high photocatalytic activity and structural stability over multiple uses, demonstrating their potential for long-term application in environmental remediation.

## 3 Conclusion

This study demonstrates that magnetic Covalent Organic Frameworks (COFs) functionalized with Fe₃O₄ nanoparticles (Fe₃O₄@HMN-COF, Fe₃O₄@MAN-COF, and Fe₃O₄@SIN-COF) are highly efficient adsorbents for removing imidacloprid (IMI) from water. These COFs, engineered with nitrogen-rich structures and extensive π-electron systems, exhibit superior adsorption through π-π interactions, hydrophobic interactions, and hydrogen bonding. Among the COFs tested, Fe₃O₄@HMN-COF displayed the highest adsorption capacity. Kinetic studies suggested that the adsorption process involved chemisorption, with adsorption capacities of 600 mg/g for Fe₃O₄@HMN-COF, 480 mg/g for Fe₃O₄@MAN-COF, and 375 mg/g for Fe₃O₄@SIN-COF. Thermodynamic analyses indicated that the adsorption process was spontaneous and endothermic. Reusability tests demonstrated minimal capacity loss over multiple cycles, and practical applications in honey and fruit samples confirmed the high efficacy of these materials. Additionally, optimized photocatalytic degradation of imidacloprid using these COFs proved highly effective, with Fe₃O₄@HMN-COF achieving 98.5 % efficiency under optimal conditions. These findings highlight the potential of Fe₃O₄-functionalized COFs, particularly Fe₃O₄@HMN-COF, as promising solutions for mitigating pesticide contamination in water and ensuring environmental sustainability.

## CRediT authorship contribution statement

**Shaikha S. AlNeyadi:** Writing – review & editing, Writing – original draft, Visualization, Validation, Supervision, Software, Resources, Project administration, Methodology, Investigation, Funding acquisition, Formal analysis, Data curation, Conceptualization. **Mohammed T. Alhassani:** Data curation. **Muneb R. Mukhtar:** Methodology. **Hamad K. Alblooshi:** Data curation. **Sultan A. Jama:** Methodology. **Ibrahim Al Mujaini:** Methodology. **Ali S. Aleissaee:** Data curation.

## Data and Code availability

Data included in the article/supplementary material is referenced in the article.

## Declaration of competing interest

The authors declare the following financial interests/personal relationships which may be considered as potential competing interests:Shaikha alneyadi reports financial support was provided by 10.13039/501100006013United Arab Emirates University. Shaikha alneyadi reports a relationship with United Arab Emirates University that includes: employment. If there are other authors, they declare that they have no known competing financial interests or personal relationships that could have appeared to influence the work reported in this paper.
