# Supplementary material for: Hydrophilic magnetic COFs: The Answer to photocatalytic degradation and removal of imidacloprid insecticide
Source: Heliyon. 2024 Oct 11;10(20):e39042. doi: 10.1016/j.heliyon.2024.e39042 (PMC11532256; doi:10.1016/j.heliyon.2024.e39042)
Supplement: Multimedia component 1 [file mmc1.docx]

Supporting Information

**Hydrophilic Magnetic COFs: The Answer to Photocatalytic Degradation and Removal of Imidacloprid Insecticide**

Shaikha S. AlNeyadi ^*a^, Mohammed T. Alhassani ^a^, Muneb R. Mukhtar,^a^ Hamad K. Alblooshi,^a^ Sultan A. Jama,^a^ Ibrahim Al Mujaini ^a^, Ali S. Aleissaee ^a^

[a] Dr. S.S. Al Neyadi, M. T. Alhassani, H. K. Alblooshi, M. R. Mukhtar, S. A. Jama, I. Al Mujaini, A. S. Aleissaee

Department of Chemistry

College of Science, UAE University

Al-Ain 15551, United Arab Emirates

E-mail: shaikha.alneyadi@uaeu.ac.ae (S.S.A.N.)

*To whom correspondence should be addressed: shaikha.alneyadi@uaeu.ac.ae (S.S.A.N.)

**Table of Contents**

| **Section S1** | Materials and General Methods | S3 |
| --- | --- | --- |
| **Section S2** | Synthesis of raw materials | S3−S7 |
| **Section S3** | Characterizations of raw materials | S8−S12 |
| **Section S4** | Synthesis of COFs and Magnetic COFs | S13−S14 |
| **Section S5** | Characterization of COFs | S15−S17 |
| **Section S6** | Adsorption experiments | S17−S24 |
| **Section S7** | Photocatalytic Degradation Studies | S25−S30 |
| **Section S8** | References | S30 |

# Section S1: Materials and General Methods

**Chemicals and Reagents:** All reagents and chemicals were obtained from Sigma Aldrich (St. Louis, MO, USA) and were used as received without additional purification. Ultrahigh-purity nitrogen (99.999% purity) from Air Products was employed for the sorption experiments. The chemicals and solvents used in the described procedures include 4-bromobenzonitrile (C_7_H_4_BrN), trifluoromethanesulfonic acid (CF_3_SO_3_H), deionized water (H_2_O), n-butyllithium (n-BuLi, C_4_H_9_Li), dimethylformamide (DMF, C_3_H_7_NO), 3M hydrochloric acid (HCl), chloroform (CHCl_3_), brine (NaCl solution), sodium sulfate (Na_2_SO_4_), petroleum ether, dichloromethane (CH_2_Cl_2_), ethanol (C_2_H_5_OH), concentrated sulfuric acid (H_2_SO_4_), cesium carbonate (Cs_2_CO_3_), 2-bromoethyl methyl ether (C_3_H_7_BrO), acetone (C_3_H_6_O), hydrazine hydrate (N_2_H_4_·H_2_O), 4-nitrobenzoic acid (C_7_H_5_NO_4_), 4-bromo-2-nitrobenzoic acid (C_7_H_4_BrNO_4_), potassium carbonate (K_2_CO_3_), copper powder (Cu), ethyl acetate (C4H8O2), anhydrous sodium sulfate (Na_2_SO_4_), Fe₃O₄ nanoparticles (20 nm, CAS 1317-61-9), sodium hydroxide (NaOH), hexamethylenetetramine (C_6_H_12_N_4_), trifluoroacetic acid (TFA, C_2_HF_3_O_2_), methanol (CH_3_OH), thionyl chloride (SOCl_2_), benzyl bromide (C_7_H_7_Br), *p*-dioxane (C_4_H_8_O_2_), palladium(II) chloride (Pd(dppf)Cl_2_), 10% palladium on carbon (Pd/C), tetrahydrofuran (THF, C_4_H_8_O), and methanol (CH_3_OH).

**Analytical Techniques:** Thin-layer chromatography was performed on silica gel glass plates (Silica gel, 60 F254, Fluka, Merck, Darmstadt, Germany), and column chromatography was conducted using Kieselgel S (silica gel S, 0.063–0.1 mm, Merck, Darmstadt, Germany). Melting points were determined using a Gallenkamp apparatus (Toledo, OH, USA) and were corrected. Elemental analysis was carried out with a Leco Model CHN-600 elemental analyzer (Ontario, Canada). Fourier-transform infrared (FTIR) spectra were obtained using KBr pellets on a Thermo Nicolet model 470 FT-IR spectrophotometer (Thermo Scientific, Waltham, MA, USA). Nuclear magnetic resonance (NMR) spectra were recorded on a Varian-400 MHz spectrometer (^1^H-NMR at 400 MHz and ^13^C-NMR at 100 MHz; Agilent Technologies, Santa Clara, CA, USA) with dimethyl sulfoxide-*d_6_* (DMSO-*d_6_*) as the solvent. Tetramethylsilane (TMS) served as an internal reference, with chemical shifts reported in parts per million (δ values, ppm). Scanning electron microscopy (SEM) images were acquired using an FEI SEM Quanta Inspect S50 scanning electron microscope (ThermoFisher PN1113094) operated at an accelerating voltage of 15–30 kV. Powder X-ray diffraction (PXRD) analysis was conducted on a Shimadzu-6100 PXRD diffractometer (Shimadzu-series, Kyoto, Japan) with Cu-Kα radiation (λ = 1.542 Å). Diffraction data were collected within the 2θ range of 20–50° at a rate of 1 °C/min, under room temperature and atmospheric pressure. Nitrogen sorption measurements and pore size analyses were performed using a PMI BET Sorptometer (BET-201-AEL, PMI, USA) with measurements at 77 K using a liquid nitrogen bath. Thermogravimetric analysis (TGA) was conducted on a 0.2 g sample, heated to 600 °C at a rate of 5 °C/min, to monitor weight changes as a function of temperature. Liquid chromatography with tandem mass spectrometry (LC-MS/MS) was performed using an Agilent 6460 Triple Quad LC/MS system (Agilent Technologies, Santa Clara, CA, USA) for detailed compound analysis. Gas Chromatography-Mass Spectrometry (GC-MS) was performed using an Agilent 7890B GC System coupled with a 5977B Mass Selective Detector (Agilent Technologies, Santa Clara, CA, USA). Inductively Coupled Plasma-Atomic Absorption Spectroscopy (ICP-AAS) was performed using a PerkinElmer AAnalyst 800 system (PerkinElmer, Waltham, MA, USA).

**Section S2: Synthesis of raw materials**

**4,4',4''-(1,3,5-triazine-2,4,6-triyl)tribenzaldehyde (L1).** To a solution of 4 g (22 mmol) of 4-bromobenzonitrile at 0 °C, 3.54 mL (6 g, 40 mmol) of trifluoromethanesulfonic acid was slowly added and stirred for 30 minutes. The mixture was then stirred at room temperature overnight. The resulting mixture was washed with 100 mL of deionized water and filtered under vacuum, yielding 3.95 g of a white solid with a 99% yield. A portion of the product (1.46 g, 3 mmol) was dissolved in 250 mL of dry THF under a nitrogen atmosphere. To this solution, 12 mL (30 mmol) of n-BuLi was added dropwise at −78 °C while stirring. After stirring for 3 hours, 5 mL of DMF was added to the red solution at −78 °C, and the reaction mixture was stirred for an additional 12 hours at room temperature. The mixture was then acidified with 50 mL of 3 M HCl aqueous solution. The organic volatiles were partially removed by evaporation under reduced pressure, and the product was extracted with CHCl_3_. The organic layer was washed with brine, dried over Na_2_SO_4_, and concentrated in vacuo. Recrystallization from a mixture of petroleum ether and CH_2_Cl_2_ afforded **L1** as a white solid with a 64% yield; IR (KBr, cm^-1^): 1705 (C=O), 1607 (C=N), 1583 (C=C); ^1^H-NMR (DMSO-*d_6_*, 400 MHz) (*δ, ppm*): 8.17 (d,2H, aromtic), 8.96 (d, 2H, aromatic), 10.17 (s, 3H, CHO); ^13^C-NMR (DMSO-*d_6_*, 100 MHz) (*δ, ppm*): 115.5, 117.0, 131.4, 132.4, 147.6 and 170.0.

**Scheme S1.** Synthesis of **L1**

**2,5-bis(2-methoxyethoxy)terephthalohydrazide (L2). ).** A solution of compound **1** (3 g, 15.2 mmol) in ethanol (60 mL) was carefully added to concentrated H_2_SO_4_ (12 mL) and refluxed at 84 °C for 18 hours. The mixture was then filtered and washed with iced water. The resulting product was dried, yielding a 96% recovery. Compound **2** (1.20 g, 4.76 mmol), Cs_2_CO_3_ (10.41 g, 31.90 mmol), and 2-bromoethyl methyl ether (1.39 g, 10.00 mmol) were suspended in acetone (42 mL). The mixture was refluxed at 60 °C until the starting material disappeared, as monitored by TLC. After filtering the mixture and evaporating the solvent, the residue was dissolved in CH_2_Cl_2_, washed with water, and dried over Na_2_SO_4_. The solvent was removed under reduced pressure, yielding the crude product. This crude product was purified by column chromatography using a petroleum ether/ethyl acetate mixture (v/v = 6:1), resulting in the target product **3** as a white solid with a 56% yield. Compound **3** (0.85 mg, 2.3 mmol), hydrazine hydrate (21.6 mL, 61.9 mmol), and ethanol (36 mL, 48.4 mmol) were added to a flask and refluxed at 84 °C for 20 hours. The mixture was then cooled to -15 °C, and the product was filtered and dried under reduced pressure, yielding **L2** as a white solid with a 96% yield; IR (KBr, cm^-1^): 3517, 3452 (NH_2_), 3409 (NH), 1648 (C=O), 1572 (C=C); ^1^H-NMR (DMSO-*d_6_*, 400 MHz) (*δ, ppm*): 3.31 (s, 6H, CH_3_), 3.66 (d, 4H, CH_2_), 4.18 (d, 4H, CH_2_), 4.57 (s, 4H, NH_2_), 7.47 (s, 2H, aromtic), 9.46 (s, 2H, OH); ^13^C-NMR (DMSO-*d_6_*, 100 MHz) (*δ, ppm*): 58.7, 69.2, 70.4, 116.2, 125.4, 150.5, 163.7.

**Scheme S2.** Synthesis of **L2**

**4,4'-diamino-[1,1'-biphenyl]-2,2'-dicarboxylic acid (L3).** Equimolar amounts of 4-nitrobenzoic acid (5 g, 30.2 mmol) and 4-bromo-2-nitrobenzoic acid (7.5 g, 30.2 mmol) were added to a round-bottom flask along with potassium carbonate (8.3 g, 60.4 mmol) and copper powder (0.6 g, 3.02 mmol) in dimethylformamide (100 mL) under a nitrogen atmosphere. The mixture was heated to 170°C and stirred for 6 hours. After cooling to room temperature, the mixture was filtered, and the filtrate was extracted with ethyl acetate (3 × 100 mL). The combined organic layers were dried over anhydrous sodium sulfate and the solvent was removed by rotary evaporation to yield 4,4'-dinitro-[1,1'-biphenyl]-2,2'-dicarboxylic acid. For the reduction, 4,4'-dinitro-[1,1'-biphenyl]-2,2'-dicarboxylic acid (10 g, 28.2 mmol) was dissolved in a mixture of ethanol (100 mL) and water (50 mL), and iron powder (15 g, 268.2 mmol) and hydrochloric acid (50 mL) were added. The mixture was refluxed for 4 hours, then cooled to room temperature. The solid residue was filtered off and the pH of the filtrate was adjusted to neutral with sodium hydroxide. The filtrate was extracted with ethyl acetate (3 × 100 mL), and the combined organic layers were dried over anhydrous sodium sulfate. After removing the solvent by rotary evaporation, the crude product was recrystallized from hot ethanol to yield 4,4'-diamino-[1,1'-biphenyl]-2,2'-dicarboxylic acid as a pure white solid (78%); IR (KBr, cm^-1^): 3517 (OH), 3450, 3384 (NH_2_), 1699 (C=O), 1572 (C=C); ^1^H-NMR (DMSO-*d_6_*, 400 MHz) (*δ, ppm*): 5.21(brs, 4H, NH_2_), 6.59 (d,2H, aromtic), 6.70 (d, 2H, aromatic), 6.96 (s, 2H, aromatic), 11.86 (brs, 2H, OH); ^13^C-NMR (DMSO-*d_6_*, 100 MHz) (*δ, ppm*): 114.9, 116.5, 130.9, 131.9, 147.1, 169.5.

**Scheme S3.** Synthesis of **L3**

**2,4,6-trihydroxybenzene-1,3,5-tricarbaldehyde (L4).** 1,3,5-Trihydroxybenzene (3 g, 23.8 mmol) and hexamethylenetetramine (8.34 g, 59.5 mmol) were added to a round-bottom flask under a nitrogen atmosphere. The mixture was then heated to 100°C and stirred for 2.5 hours in trifluoroacetic acid (47 mL). Subsequently, hydrochloric acid (50 mL) was added, and the mixture was maintained at 100°C for an additional hour. After cooling to room temperature, the filtrate was extracted with dichloromethane (3 × 100 mL), and the organic layer was separated and dried over anhydrous sodium sulfate. The crude product was obtained by rotary evaporation of the solvent. The final product was collected as a light pink powder after recrystallization from hot ethanol, yielding a pure white solid (620 mg, 18%); IR (KBr, cm^-1^): 3355 (OH), 1652 (C=O), 1603 (C=C); ^1^H-NMR (DMSO-*d_6_*, 400 MHz) (*δ, ppm*): 6.47 (brs, 3H, OH), 9.96 (s, 3H, CHO); ^13^C-NMR (DMSO-*d_6_*, 100 MHz) (*δ, ppm*): 103.7, 174.1, 191.6.

**Scheme S4.** Synthesis of **L4**

**3,3'-dihydroxy-[1,1'-biphenyl]-4,4'-dicarbaldehyde(L4).** The synthesis of 3,3'-dihydroxy-[1,1'-biphenyl]-4,4'-dicarbaldehyde **(L5)** began by slowly adding 5 mL of thionyl chloride (SOCl_2_) dropwise to a solution containing 4-bromo-2-hydroxybenzoic acid **1** (5 g, 23 mmol) in methanol (39.6 g, 1.24 mol) within an ice bath. Afterward, the reaction mixture was heated to 75°C and stirred overnight using an oil bath. Upon completion, the mixture was cooled to room temperature, extracted with ethyl acetate (EtOAc) and water, and the organic layers were dried, filtered, and evaporated. The obtained crude product was subjected to column chromatography, yielding compound **2** as a white solid with a 90% isolated yield. Following this, Na_2_CO_3_ (4.15 g, 39 mmol) was introduced to a solution of methyl 4-bromo-2-hydroxybenzoate **2** (3 g, 13 mmol) in MeCN (150 mL) at RT, followed by benzyl bromide addition, and overnight stirring at 80°C. After cooling to RT, the mixture was filtered to remove insoluble salts, and the organic phase was dried, filtered, and evaporated. Column chromatography purification resulted in compound **3** as a white solid with a 78% isolated yield. Later, a mixture of **3** and compound **4** (1 g, 3.11 mmol) was dissolved in a p-dioxane/H_2_O mixed solvent (4:1 v/v), deoxygenated, and stirred at 85°C for 24 hours upon addition of K_2_CO_3_ and Pd(dppf)Cl_2_. The reaction mixture was then cooled to RT, filtered, and the organic phases were dried, filtered, and evaporated. Column chromatography purification afforded compound **5** as a white solid with an 83% isolated yield. Finally, compound **5** (2 g, 4.15 mmol) was dissolved in THF and MeOH, added with 10% by weight Pd/C, and subjected to hydrogenation. After filtration, acidification, and collection of the precipitate, compound **6** was obtained as a white powder with a 70% isolated yield. IR (KBr, cm^-1^): 3451 (OH), 1637 (C=O), 1572 (C=C); ^1^H-NMR (DMSO-*d_6_*, 400 MHz) (*δ, ppm*): 7.21 (m, 4H, aromtic), 7.72 (d, 2H, aromatic), 10.27 (s, 2H, CHO), 10.92 (s, 2H, OH) ; ^13^C-NMR (DMSO-*d_6_*, 100 MHz) (*δ, ppm*): 115.8, 118.7, 122.6, 130.6, 146.7, 161.4, 191.3.

**Scheme S5.** Synthesis of **L5**

# Section S3: Characterization of raw materials

#
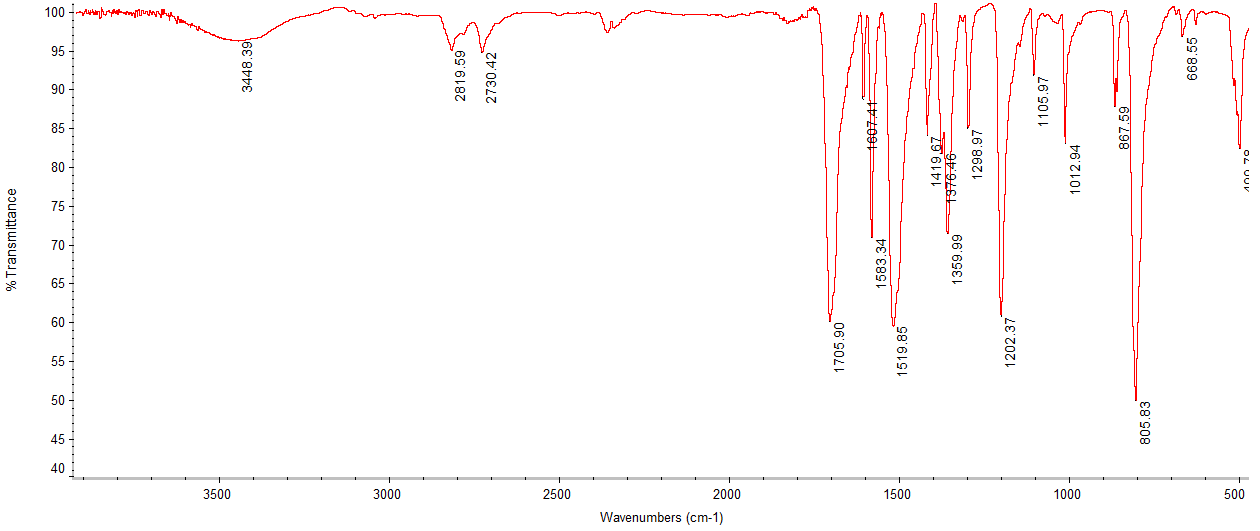


a

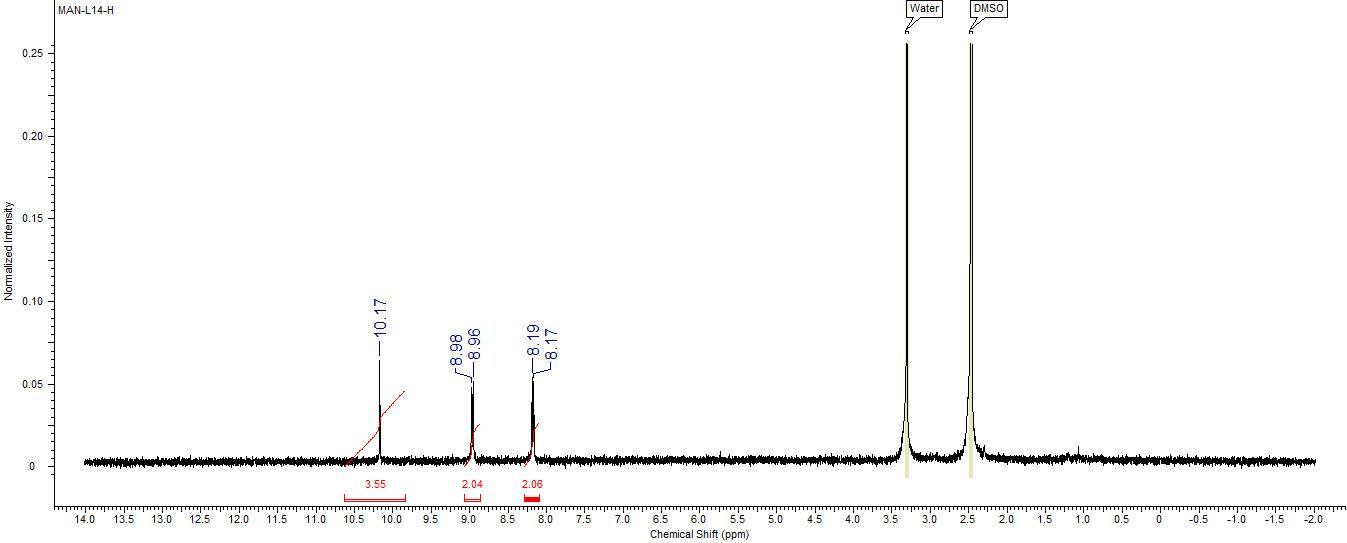


b


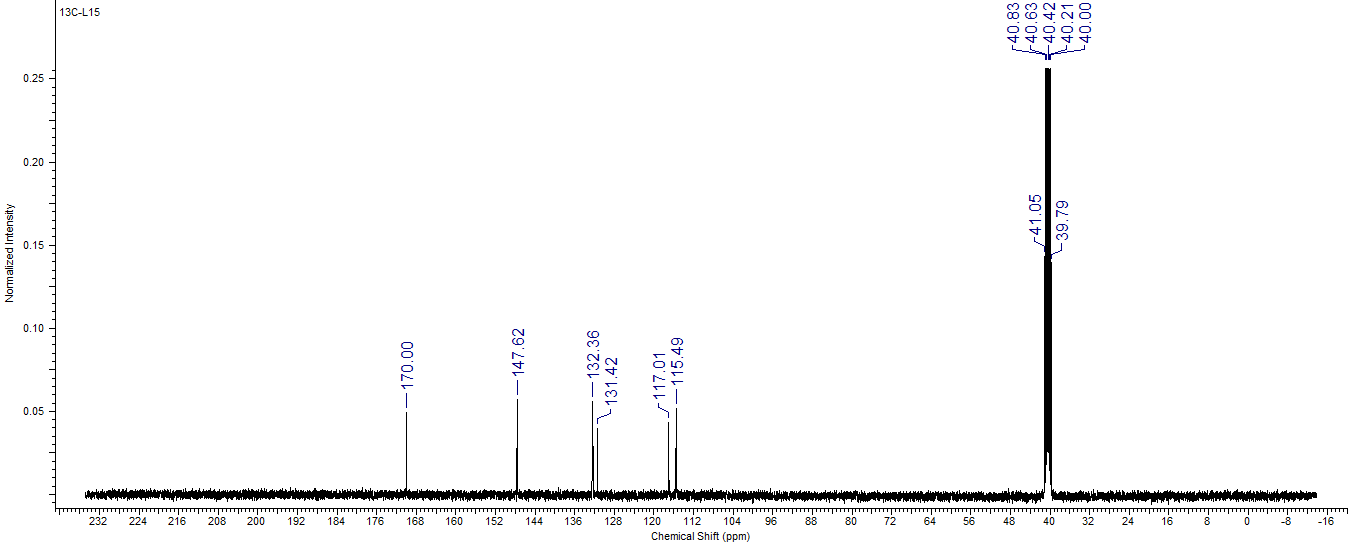


c

**Fig S1**. a) IR spectrum; (b) ^1^H-NMR spectrum; (c) ^13^C-NMR spectrum for **L1**


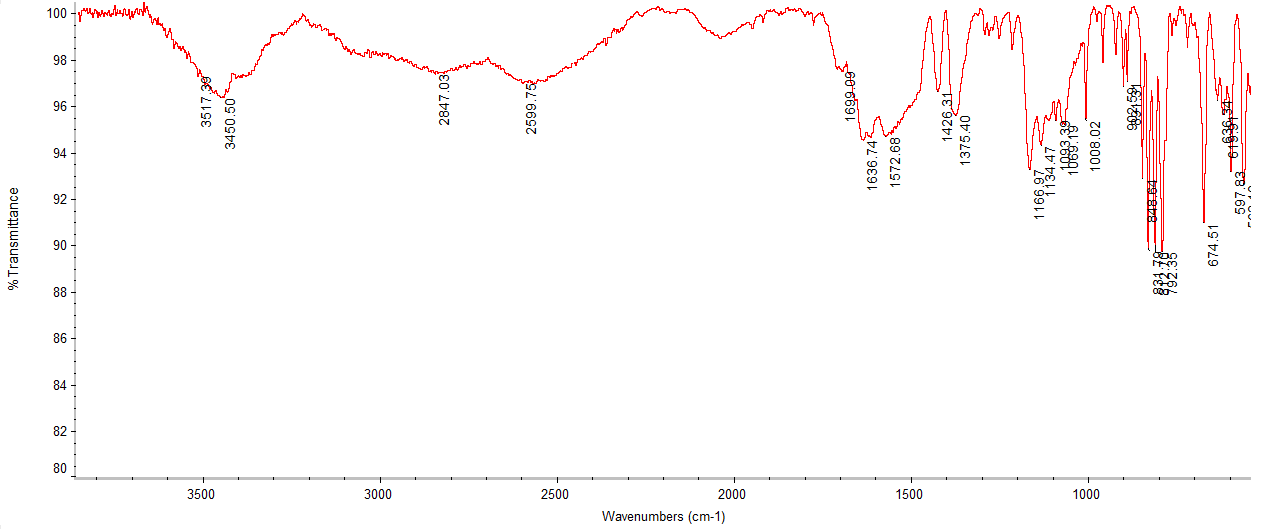


a

**2,5-bis(2-methoxyethoxy)terephthalohydrazide**

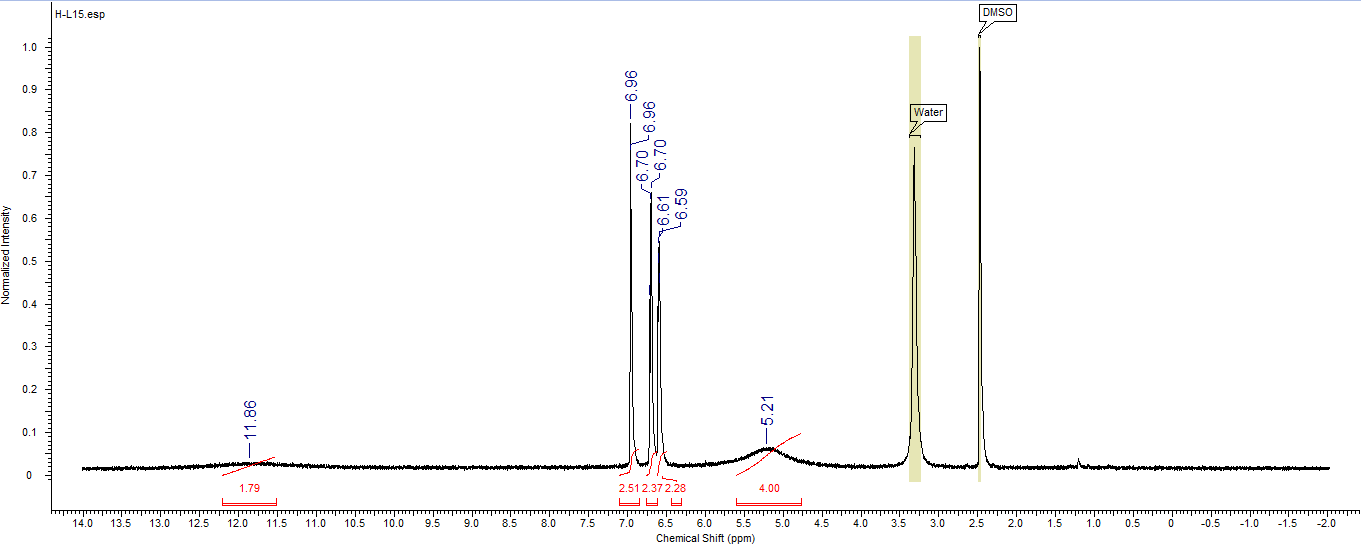


b


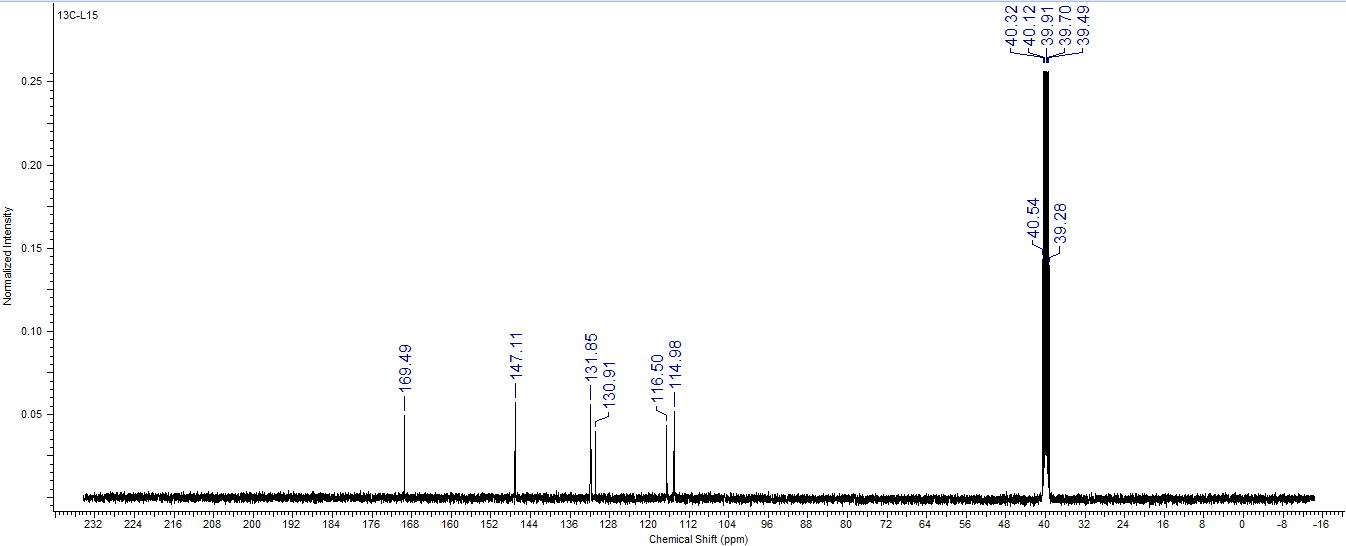


c

**Fig S2.** a) IR spectrum; (b) ^1^H-NMR spectrum; (c) ^13^C-NMR spectrum for **L2**


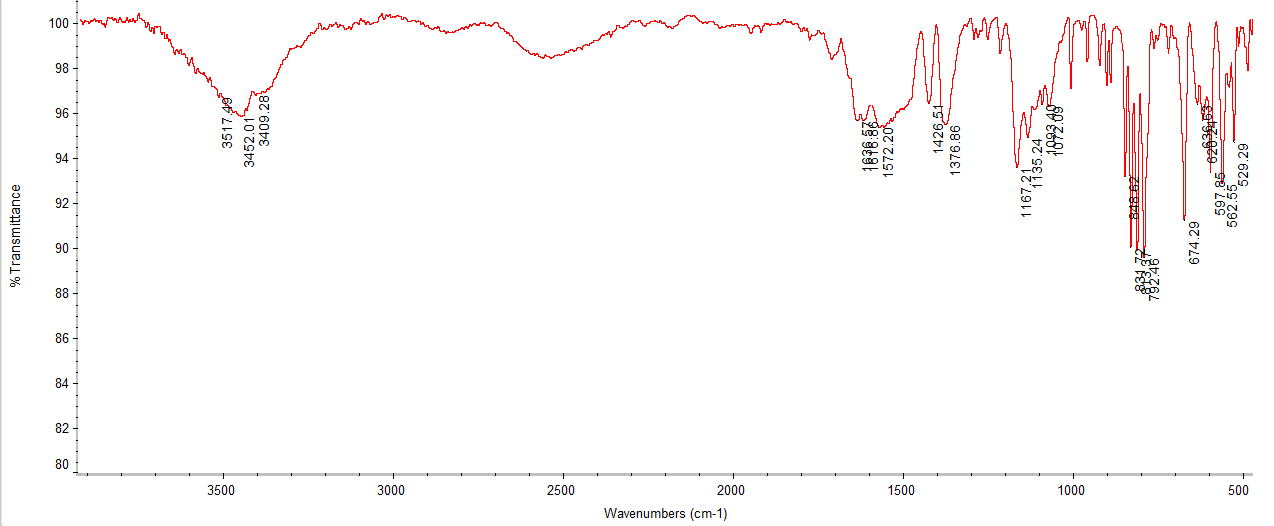


a

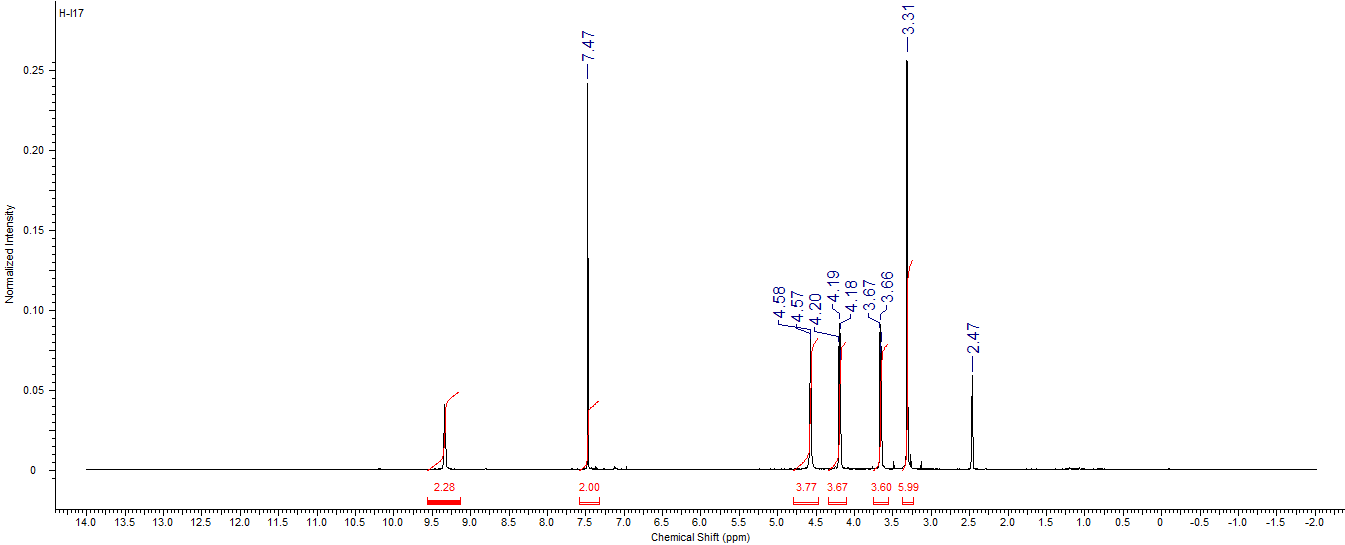


b


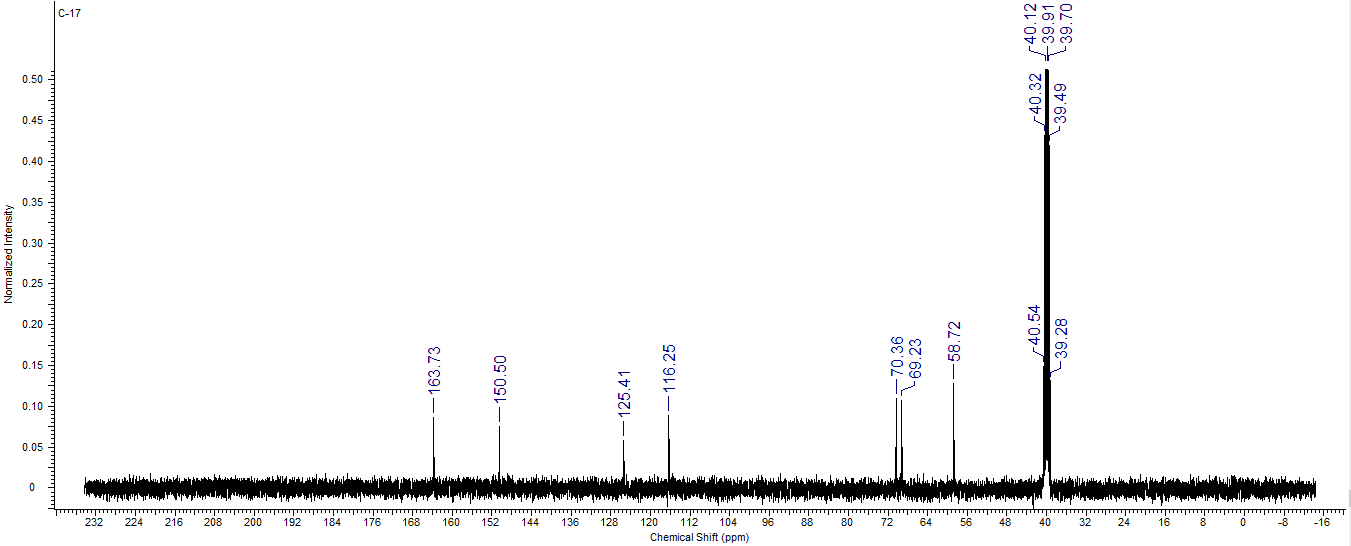


c

**Fig S3.** a) IR spectrum; (b) ^1^H-NMR spectrum; (c) ^13^C-NMR spectrum for **L3**


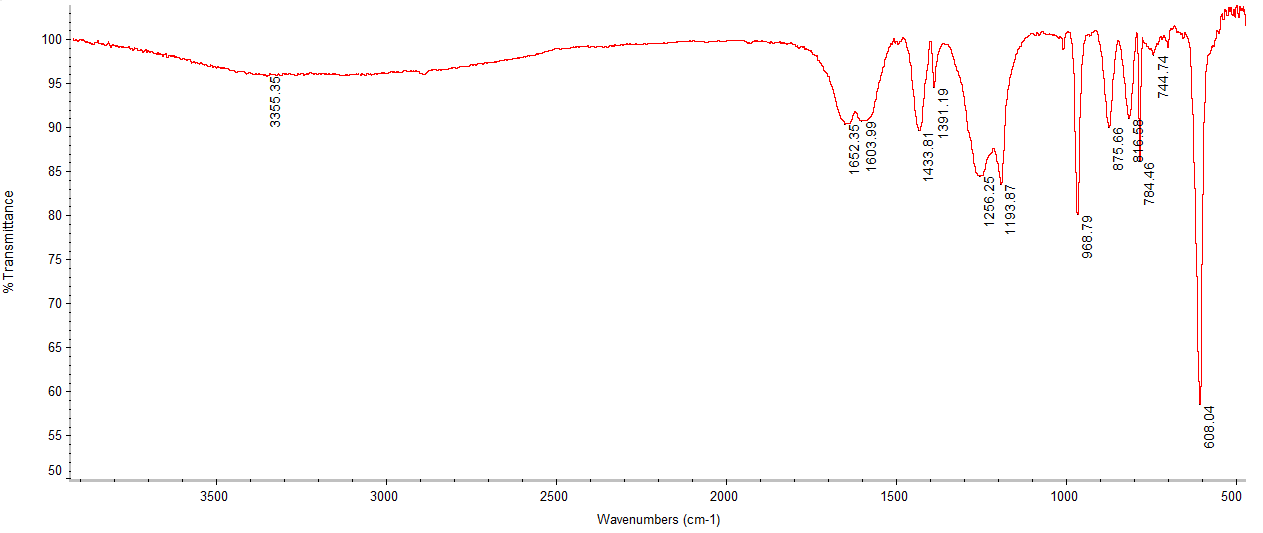


a


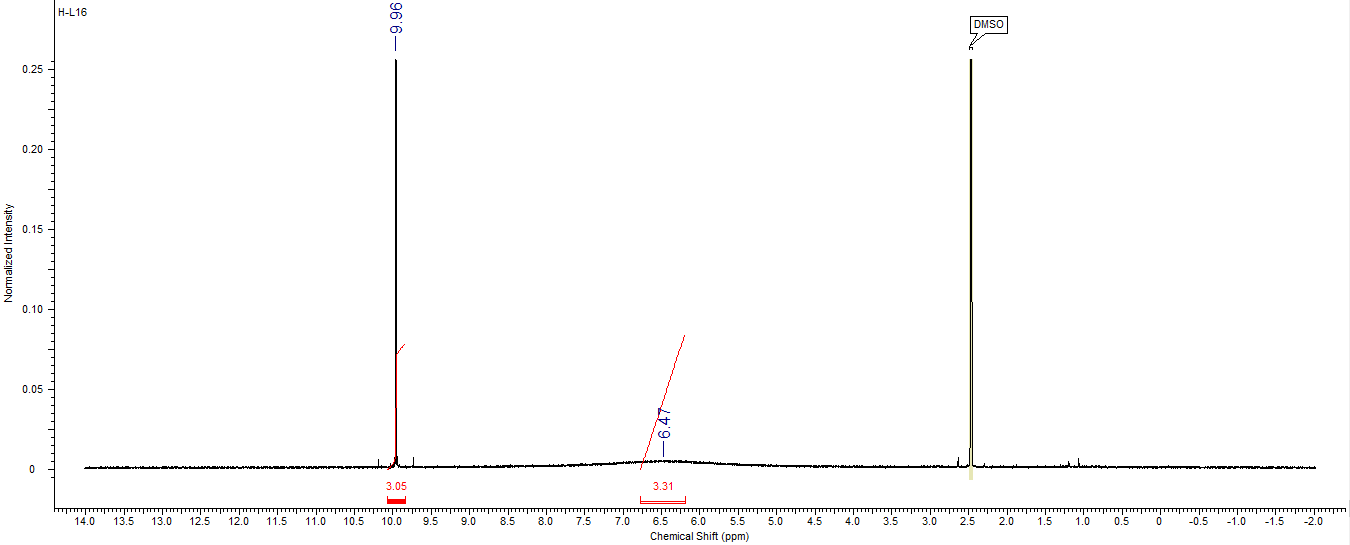


b


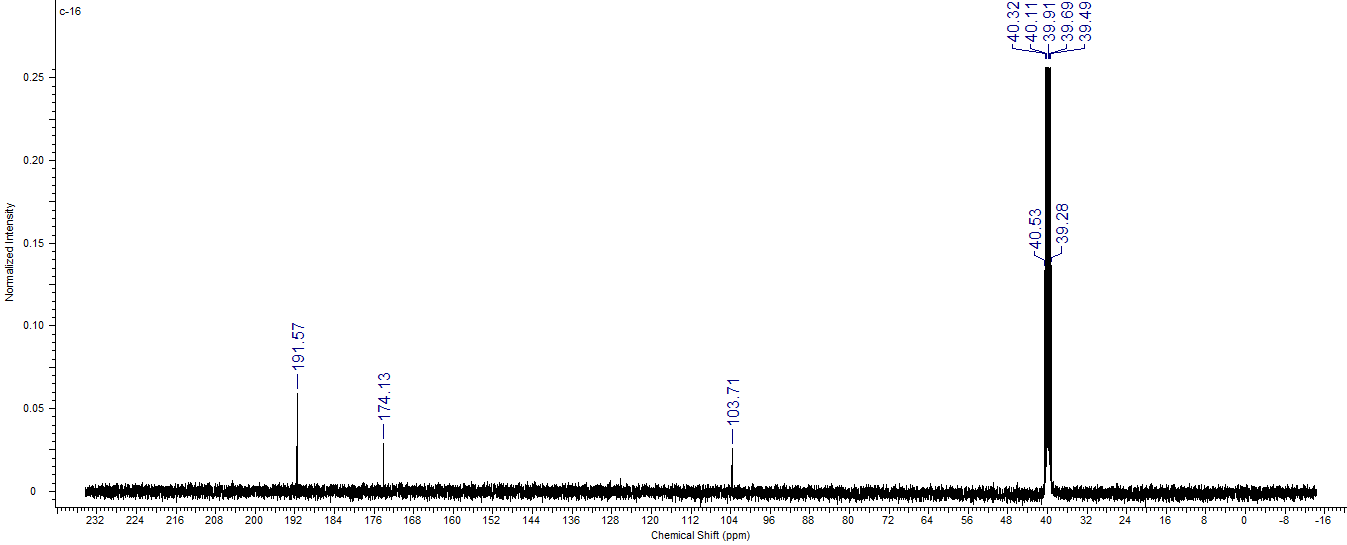


c

**Fig S4**. a) IR spectrum; (b) ^1^H-NMR spectrum; (c) ^13^C-NMR spectrum for **L4**


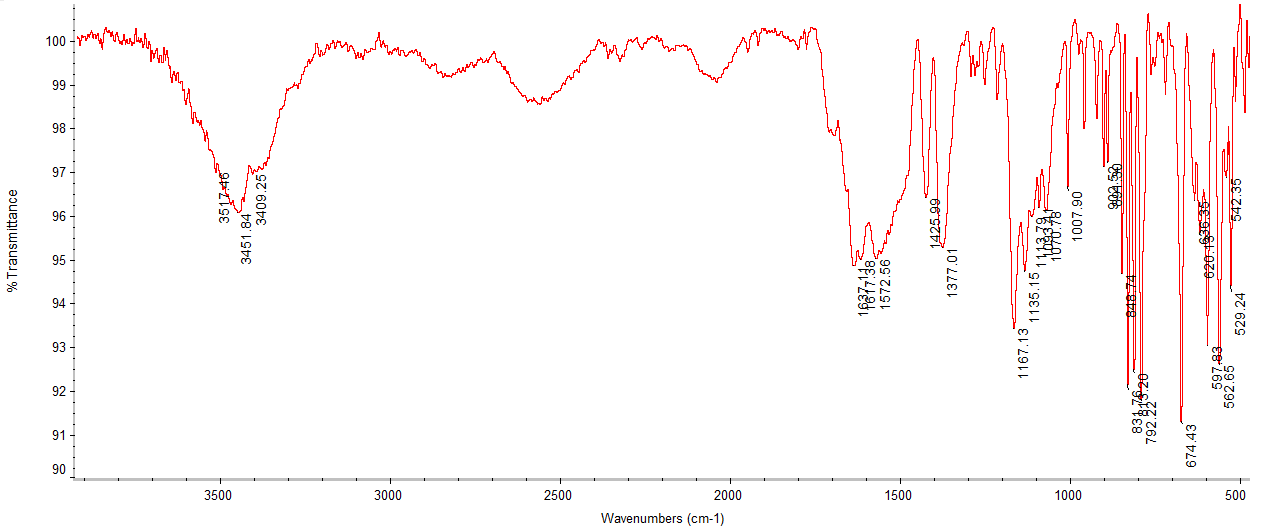


a

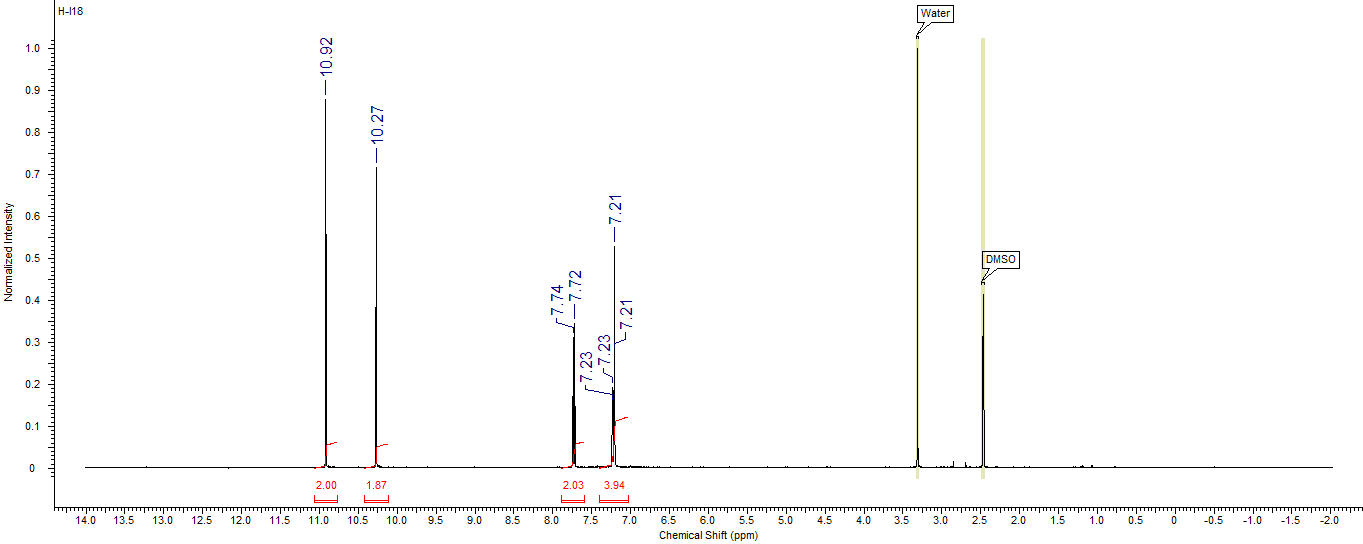


b


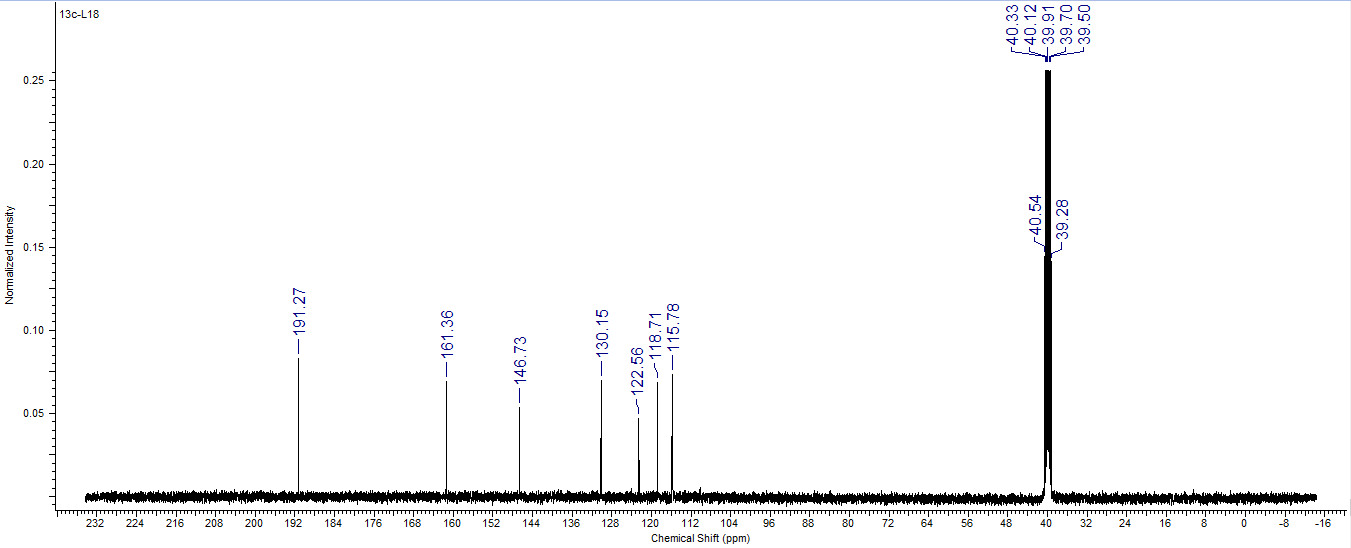


c

**Fig S5**. a) IR spectrum; (b) ^1^H-NMR spectrum; (c) ^13^C-NMR spectrum for L5

# Section S4: Synthesis of COFs

# COF-MA-N. COF synthesis involved the combination of L1 (1mmol), L3 (2 mmol), mesitylene (0.8 mL), and 1,4-dioxane (0.2 mL) in a tube, followed by ultrasonic treatment for 2 minutes. Subsequently, a 0.1 mL solution of 6 M acetic acid was introduced into the tube. Transfer reaction to autoclave and heating at 120 °C for 5 days in a static oven. Post-reaction, the mixture was cooled to room temperature and subjected to sequential washing with THF (30 mL) and acetone (30 mL). The resulting product was filtered and dried under vacuum conditions at 60 °C, yielding COF as a fluffy powder.

#

**Scheme S6**. Synthesis of MAN-COF

# COF-HMN. The synthesis of the COF involved combining L2 (1 mmol), L4 (2 mmol), mesitylene (0.8 mL), and 1,4-dioxane (0.2 mL) in a tube, followed by ultrasonic treatment for 2 minutes. Subsequently, 0.1 mL of 6 M acetic acid was added to the tube. The reaction mixture was then transferred to an autoclave and heated at 120 °C for 5 days in a static oven. After the reaction, the mixture was cooled to room temperature and washed sequentially with THF (30 mL) and acetone (30 mL). The resulting product was filtered and dried under vacuum at 60 °C, yielding the COF as a fluffy powder.

#

**Scheme S7**. Synthesis of HMN-COF

**COF-SIN.** The synthesis involved combining **L5** (1 mmol), **L3** (1.5 mmol), o-dichlorobenzene (0.8 mL), and 1,4-dioxane (0.2 mL) in a tube, followed by ultrasonic treatment for 2 minutes. Subsequently, 0.1 mL of 6 M acetic acid was introduced into the tube. The reaction mixture was then transferred to an autoclave and heated at 120 °C for 5 days in a static oven. After the reaction, the mixture was cooled to room temperature and washed sequentially with THF (30 mL) and acetone (30 mL). The resulting product was filtered and dried under vacuum at 60 °C, yielding the COF as a fluffy powder.

**Scheme S8**. Synthesis of SIN-COF

**Functionalizing COF with Fe₃O₄ Nanoparticles.** The synthesis of the COF@Fe₃O₄ composite structure was carried out at different COF-to-Fe₃O₄ ratios (1:1, 1:2, and 2:1). In a typical procedure, COF and FeCl₃·6H₂O were dissolved in 60 mL of ethylene glycol, along with 2.3 g of anhydrous sodium acetate. For the 1:1 ratio, 0.1856 g of COF and 0.6521 g of FeCl₃·6H₂O were used. For the 1:2 ratio, the FeCl₃·6H₂O amount was doubled to 1.3042 g, while the COF amount remained at 0.1856 g. For the 2:1 ratio, the COF amount was doubled to 0.3712 g, while the FeCl₃·6H₂O amount remained at 0.6521 g. The mixtures were transferred to hydrothermal reactors and heated to 200°C for 6 hours. After cooling to room temperature, the composite materials were collected using magnetic separation, washed thoroughly with methanol, and then dried under vacuum at 60°C to yield the COF@Fe₃O₄ composites for each ratio.

**
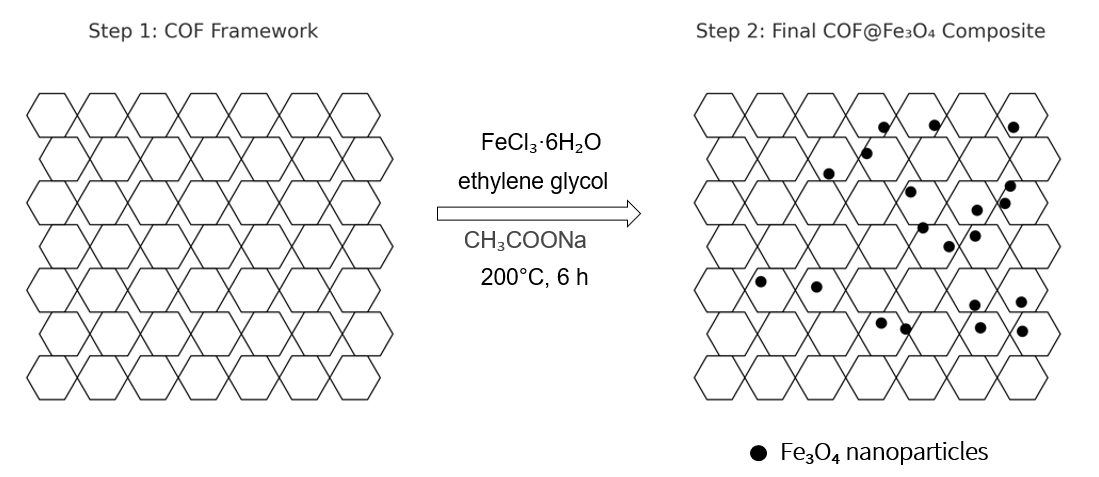
**

**Scheme S9**. Schematic of COF@Fe₃O₄ composite synthesis. **Step 1:** COF framework formation. **Step 2:** COF@Fe₃O₄ composite with Fe₃O₄ nanoparticles integrated after treatment with FeCl₃·6H₂O, ethylene glycol, and CH₃COONa at 200°C for 6 hours.

# Section S5: Characterization of COFs

#
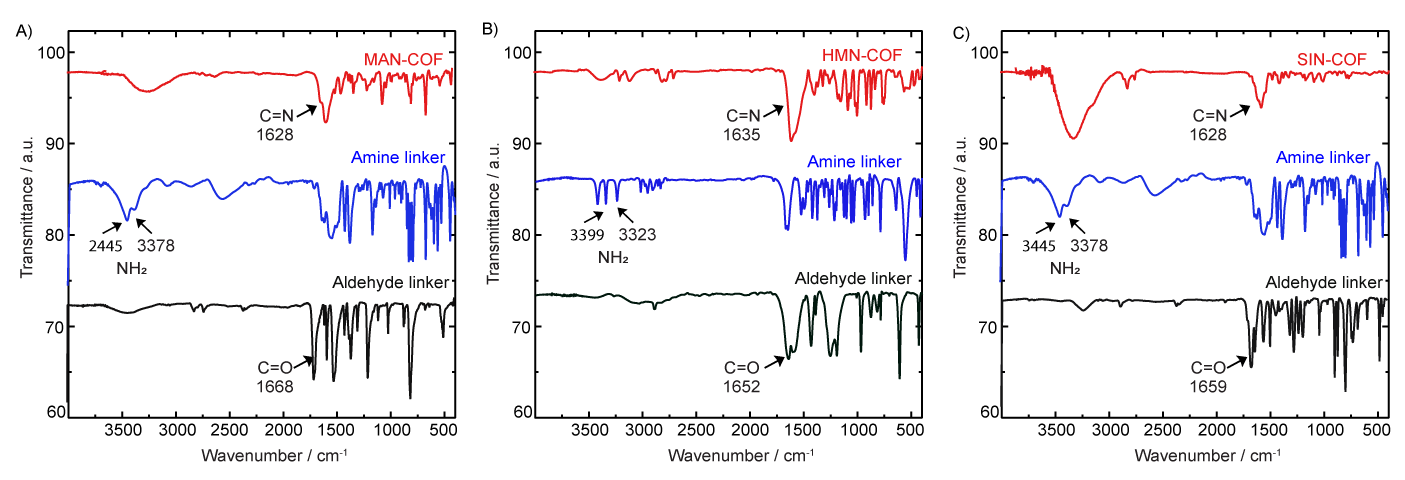


# Fig S6. FTIR spectra of (A) MAN-COF, (B) HMN-COF, and (C) SIN-COF, showing the imine (C=N) stretching peaks at 1628 cm⁻¹, 1635 cm⁻¹, and 1628 cm⁻¹, respectively. The spectra also display NH₂ peaks from the amine linkers and C=O peaks from the aldehyde linkers, confirming the successful formation of the COFs.

#
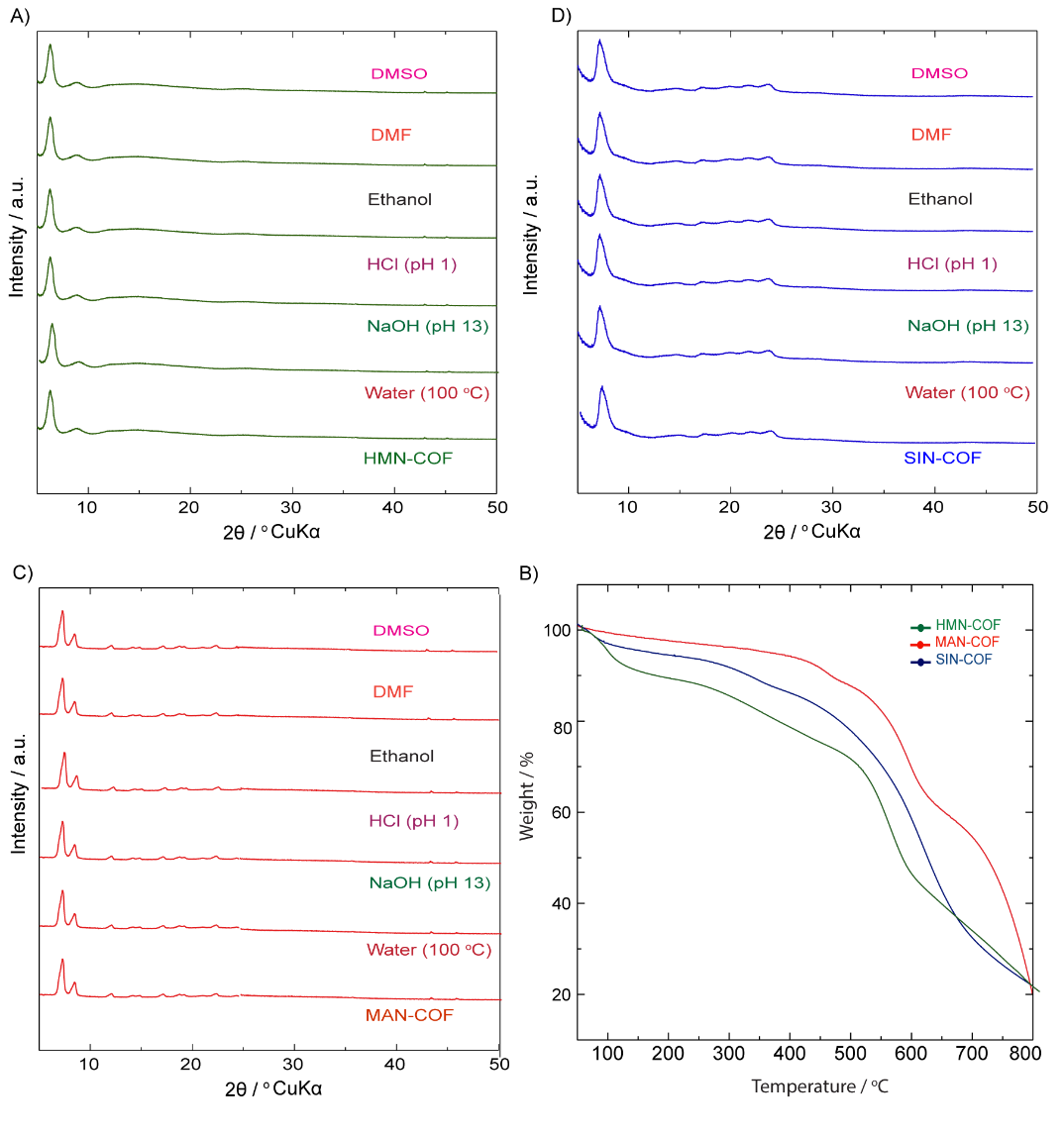


**Fig S7**. (A, C, D) XRD patterns of HMN-COF, MAN-COF, and SIN-COF after treatment with various solvents and conditions, showing retained crystallinity. (B) TGA curves of HMN-COF, MAN-COF, and SIN-COF, indicating thermal stability up to 400 °C.


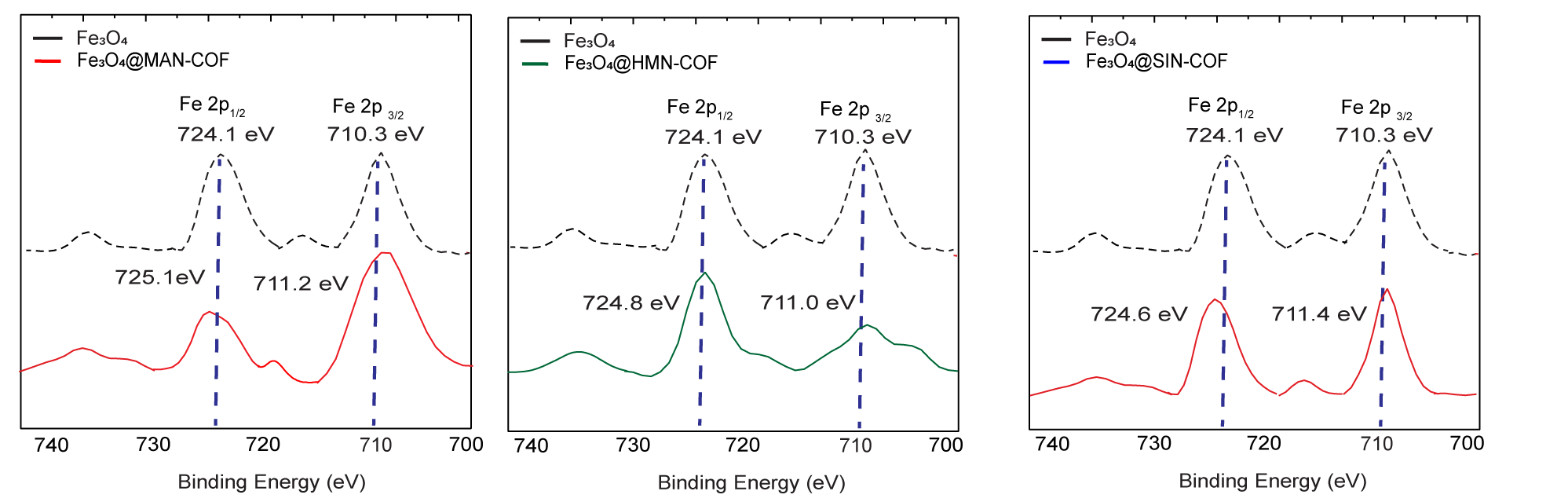


**Fig S8**. XPS spectra of Fe 2p for (left) Fe₃O₄@MAN-COF, (middle) Fe₃O₄@HMN-COF, and (right) Fe₃O₄@SIN-COF, showing the binding energy peaks at Fe 2p₁/₂ and Fe 2p₃/₂ for each sample compared to Fe₃O₄. The shifts in binding energy indicate successful incorporation of Fe₃O₄ into the COFs.

**Table S1:** Concentration of Fe³⁺ ions leached from magnetic COFs under different pH conditions, measured using ICP-AAS. The data demonstrates minimal leaching of Fe³⁺ ions across a range of pH environments, indicating strong chemical stability of the magnetic COFs, particularly under neutral and slightly acidic conditions.

| **Condition** | **Fe³⁺ Concentration (ppm)** |
| --- | --- |
| Neutral (pH 7) | 0.05 |
| Slightly Acidic (pH 5) | 0.10 |
| Acidic (pH 3) | 0.25 |
| Strongly Acidic (pH 1) | 0.50 |

**Water contact angle measurements.** Were conducted by manually dispensing a small droplet of deionized water (approximately 3 µL) onto the surface of the magnetic COFs using a precision syringe. The contact angle was visually observed and measured using a protractor method, where the angle between the water droplet and the surface was manually determined. Multiple measurements were taken at different locations on the sample to ensure accuracy and reproducibility, and the average contact angle was calculated from at least three independent measurements. This method allowed for an estimation of the hydrophilicity or hydrophobicity of the COF surfaces.


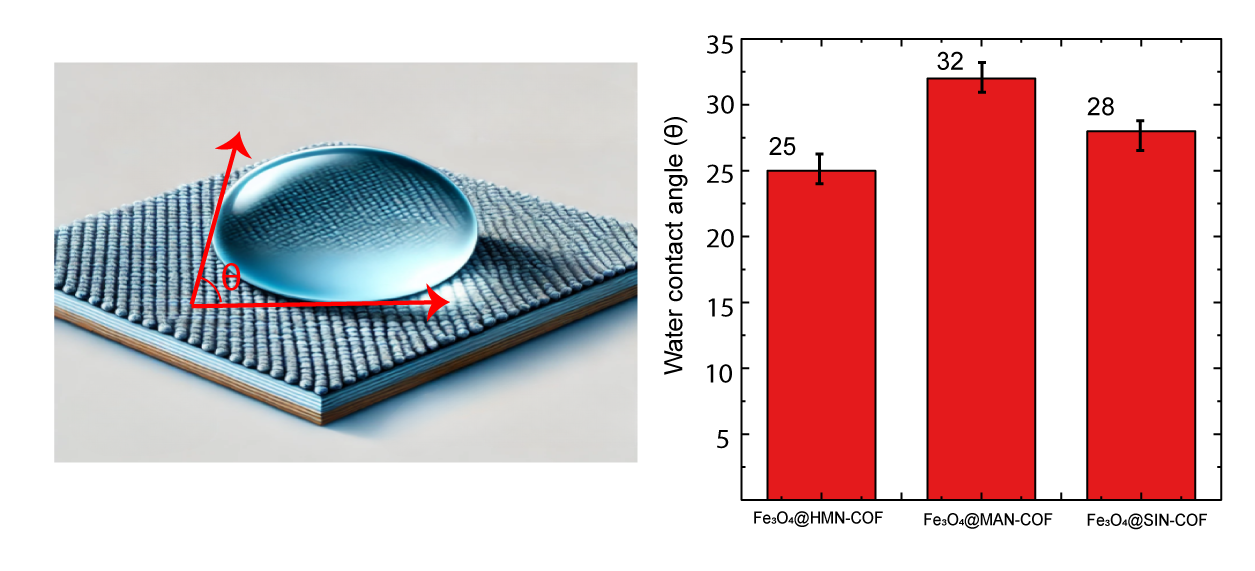


**Fig S9.** (Left) Illustration showing the water contact angle (θ) on the surface of Fe₃O₄@COF, highlighting the hydrophilic nature and the interaction between the droplet and COF surface. (Right) Bar chart of the water contact angles measured for Fe₃O₄@HMN-COF (25°), Fe₃O₄@MAN-COF (32°), and Fe₃O₄@SIN-COF (28°), indicating the varying levels of hydrophilicity across the different COF samples.

# Section S6: Adsorption experiments

**Effect of pH.** The investigation into the effect of pH on the adsorption efficiency of imidacloprid by Fe₃O₄@MAN-COF, Fe₃O₄@HMN-COF, and Fe₃O₄@SIN-COF composites involved preparing buffer solutions with pH values of 2, 3, 5, 6, 7, 8, and 9, and measuring the zeta potential of each composite to determine the point of zero charge (pH_ZPC_). Adsorption experiments were conducted by adding 50 mg of each COF composite to 50 mL of imidacloprid solution (10 mg/L) in the different buffer solutions and shaking at 200 rpm for 24 hours at room temperature to reach equilibrium. After centrifugation and filtration, the residual concentration of imidacloprid was quantified using LC-MS/MS. The adsorption capacity was calculated and plotted against pH to visualize the influence of pH on adsorption efficiency.

**Effect of adsorbent dosage.** The study investigated the effect of adsorbent dosage on the adsorption efficiency of imidacloprid by Fe₃O₄@MAN-COF, Fe₃O₄@HMN-COF, and Fe₃O₄@SIN-COF composites, using concentrations ranging from 0.05 to 0.5 g/L. Imidacloprid solutions (10 mg/L) were prepared and each COF composite was added in varying amounts to achieve the desired dosages. The mixtures were shaken at 200 rpm for 24 hours at room temperature to reach equilibrium, after which the samples were centrifuged and filtered. The residual imidacloprid concentration was quantified using LC-MS/MS. Adsorption capacity and removal efficiency were calculated and plotted against adsorbent dosage.

**Effect of ionic strength**. To evaluate the effect of ionic strength on the adsorption of imidacloprid by Fe₃O₄@HMN-COF, Fe₃O₄@MAN-COF, and Fe₃O₄@SIN-COF, prepare imidacloprid solutions (10 mg/L) and NaCl solutions with varying concentrations (10, 20, 30, and 40 mg/L). Add 50 mg of each COF composite to 50 mL of the imidacloprid solution in separate flasks, then add the NaCl solutions to achieve the desired salt concentrations. Shake the flasks at 200 rpm for 24 hours at room temperature. After equilibrium, centrifuge the samples at 4000 rpm for 10 minutes, filter the supernatant, and measure the residual imidacloprid concentration using LC-MS/MS.

**Adsorption kinetics**. To study the adsorption kinetics of imidacloprid onto Fe₃O₄@MAN-COF, Fe₃O₄@HMN-COF, and Fe₃O₄@SIN-COF, imidacloprid solutions (10 mg/L) were prepared, and 50 mg of each COF composite was added to 50 mL of the solution. The mixtures were shaken at 200 rpm at room temperature, and samples were collected at varying time intervals (1, 3, 5, 10, 20, 30, 60, and 120 minutes). After centrifugation at 4000 rpm for 10 minutes and filtration, the residual imidacloprid concentration was measured using LC-MS/MS.

**Adsorption isotherms**. To study the adsorption isotherms of imidacloprid onto Fe₃O₄@MAN-COF, Fe₃O₄@HMN-COF, and Fe₃O₄@SIN-COF, imidacloprid solutions with varying concentrations (5, 10, 20, 30, 40, 50 mg/L) were prepared. Each COF composite (50 mg) was added to 50 mL of the imidacloprid solutions in separate flasks and shaken at 200 rpm for 24 hours at room temperature to reach equilibrium. After centrifugation and filtration, the residual imidacloprid concentration was measured using LC-MS/MS, and the adsorption capacity (mg/g) was calculated.

**Adsorption thermodynamics**. To study the of imidacloprid onto Fe₃O₄@MAN-COF, Fe₃O₄@HMN-COF, and Fe₃O₄@SIN-COF, imidacloprid solutions (10 mg/L) were prepared, and 50 mg of each COF composite was added to 50 mL of the solution. The mixtures were shaken at 200 rpm in a thermostatic water bath at four temperatures: 283.0 K, 288.0 K, 298.0 K, and 308.0 K for 24 hours to reach equilibrium. After centrifugation and filtration, the residual imidacloprid concentration was measured using LC-MS/MS, and the adsorption capacity (mg/g) was calculated.

**Optimize the ratio of magnetic COF composites**. To for enhanced imidacloprid adsorption, prepare imidacloprid solutions with an initial concentration of 10 mg/L. Test pure COFs (HMN-COF, MAN-COF, and SIN-COF) and their magnetic composites with different ratios of COF to magnetic iron nanoparticles (1:1, 2:1, 1:2). Add 50 mg of each COF composite to 50 mL of the imidacloprid solution in separate flasks and shake at 200 rpm for 24 hours at room temperature. After equilibrium is reached, centrifuge the samples at 4000 rpm for 10 minutes to separate the adsorbent. Filter the supernatant through a 0.22 µm syringe filter and analyze the residual imidacloprid concentration using LC-MS/MS.

**Reusability**. To evaluate the reusability of Fe₃O₄@HMN-COF, Fe₃O₄@MAN-COF, and Fe₃O₄@SIN-COF for imidacloprid adsorption, conduct the adsorption-desorption process over five cycles using ethanol as the eluent. Initially, prepare imidacloprid solutions (10 mg/L) and add 50 mg of each COF composite to 50 mL of the solution in separate flasks. Shake the flasks at 200 rpm for 24 hours at room temperature, then centrifuge at 4000 rpm for 10 minutes and filter the supernatant to measure the residual imidacloprid concentration using LC-MS/MS. For desorption, wash the adsorbed COF composites with ethanol, shake for 24 hours, centrifuge, and filter. Reuse the regenerated COFs for the next adsorption cycle. Repeat this process for a total of five cycles. After each cycle, calculate the adsorption capacity and retention percentage of the initial capacity. Perform FT-IR analyses on the COFs before and after the cycles to confirm imidacloprid adsorption and assess stability.

**Sample preparation**. For sample preparation, environmental water samples from wells in an agricultural irrigation system, and tap water from the laboratory were collected. All water samples were filtered through a 0.45 µm membrane to eliminate particles and stored in brown glass bottles at 4°C before undergoing the extraction process. For honey samples: Dilute the samples with high-purity water at a 1:20 ratio, followed by filtration using a 0.45 μm filter membrane. For fruit samples: Weigh 1 g of homogenized sample into a 50 mL centrifuge tube. Add 10 mL of methanol and perform ultrasonic extraction for 10 minutes. Centrifuge the mixture at 8000 rpm for 5 minutes and filter the resulting supernatant.


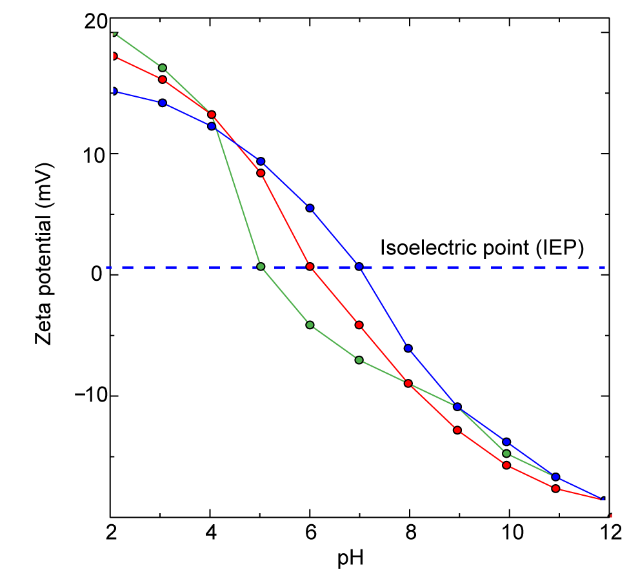


**Fig S10.** Zeta Potential vs. pH for Fe₃O₄@MAN-COF (red), Fe₃O₄@HMN-COF (green), and Fe₃O₄@SIN-COF (blue). The blue dashed line indicates the isoelectric point (IEP). The graph highlights the Zeta potential changes with pH for each COF, demonstrating their stability and charge behavior across different pH levels.


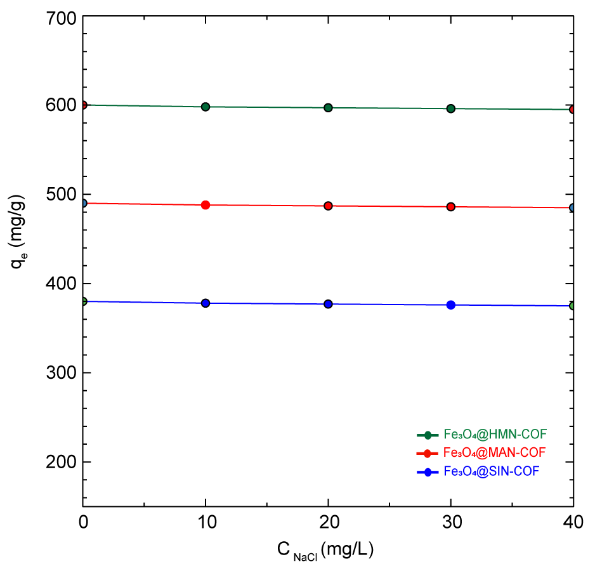


**Fig S11**: Adsorption capacity (q_e_) of Fe₃O₄@HMN-COF, Fe₃O₄@MAN-COF, and Fe₃O₄@SIN-COF as a function of NaCl concentration (C_NaCl_). The adsorption capacities were measured at various NaCl concentrations, demonstrating the stability and performance of the COFs in different ionic strength environments. The results indicate consistent adsorption capacities for all three COFs across the tested NaCl concentration range.

A

B

C

D

F

E

**Fig S12**. Kinetic models for adsorption of imidacloprid on three different composites: Fe₃O₄@MAN-COF, Fe₃O₄@HMN-COF, and Fe₃O₄@SIN-COF. (A) and (B) present the pseudo-second-order and pseudo-first-order diffusion model plots for Fe₃O₄@MAN-COF, respectively. (C) and (D) illustrate the pseudo-second-order and pseudo-first-order diffusion model plots for Fe₃O₄@HMN-COF, respectively. (E) and (F) show the pseudo-second-order and pseudo-first-order diffusion model plots for Fe₃O₄@SIN-COF, respectively.

D

C

F

E

**Fig S13**. Adsorption isotherms for imidacloprid on three different composites: Fe₃O₄@MAN-COF, Fe₃O₄@HMN-COF, and Fe₃O₄@SIN-COF. (A) Linearized Langmuir isotherms and (B) Linearized Freundlich isotherms for iodine adsorption on Fe₃O₄@MAN-COF. (C) Linearized Langmuir isotherms and (D) Linearized Freundlich isotherms for iodine adsorption on Fe₃O₄@HMN-COF. (E) Linearized Langmuir isotherms and (F) Linearized Freundlich isotherms for iodine adsorption on Fe₃O₄@SIN-COF.

**Table S2.** Adsorption capacities (q_exp_​) of various adsorbents as reported in different studies. The data highlights the performance of a wide range of materials used for adsorption purposes, including COFs, MOFs, biochars, and composites.

| **Adsorbent** | **q_exp_​ (mg/g)** | **References** |
| --- | --- | --- |
| UiO-66-NH_2_ | 83.26 | [[1](#_ENREF_1)] |
| COF-300 | 39.37 | [[2](#_ENREF_2)] |
| KOH-activated Magnetic Biochar | 313 | [[3](#_ENREF_3)] |
| Eucalyptus Woodchip Biochar | 14.75 | [[4](#_ENREF_4)] |
| Phosphoric Acid-Activated Carbon | 35.7 | [[5](#_ENREF_5)] |
| ZIF-67@MPPOP | 80.53 | [[6](#_ENREF_6)] |
| MIL-101(Cr) | 50.38 | [[7](#_ENREF_7)] |
| ZIF-67/CS@C | 189 | [[8](#_ENREF_8)] |
| HY4 zeolite | 165.8 | [[9](#_ENREF_9)] |
| U-COF | 217.2 | [[10](#_ENREF_10)] |
| **Fe₃O₄@HMN-COF** | **598.5** | **This work** |
| **Fe₃O₄@MAN-COF** | **478.0** | **This work** |
| **Fe₃O₄@SIN-COF** | **372.5** | **This work** |


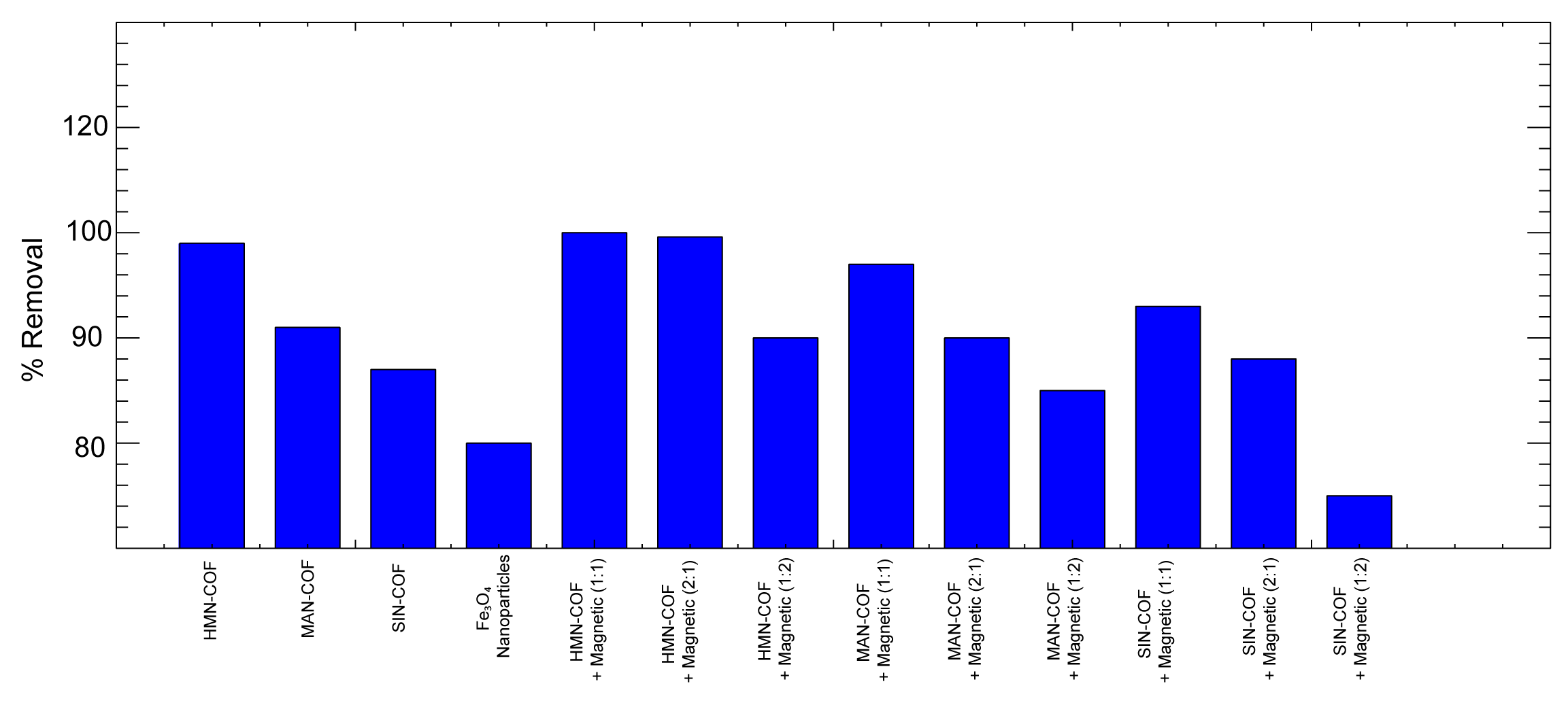


**Fig S14**. Percentage removal efficiency of different COF samples and their mixtures with iron nanoparticles. The graph compares pure HMN-COF, MAN-COF, and SIN-COF with Fe_3_​O_4_​ nanoparticles and their respective mixtures at ratios of 1:1, 2:1, and 1:2. The results show the removal efficiency for HMN-COF, MAN-COF, and SIN-COF individually and in combination with magnetic iron nanoparticles, indicating the tunability of COF and iron nanoparticle compositions for optimal removal efficiency in various applications.


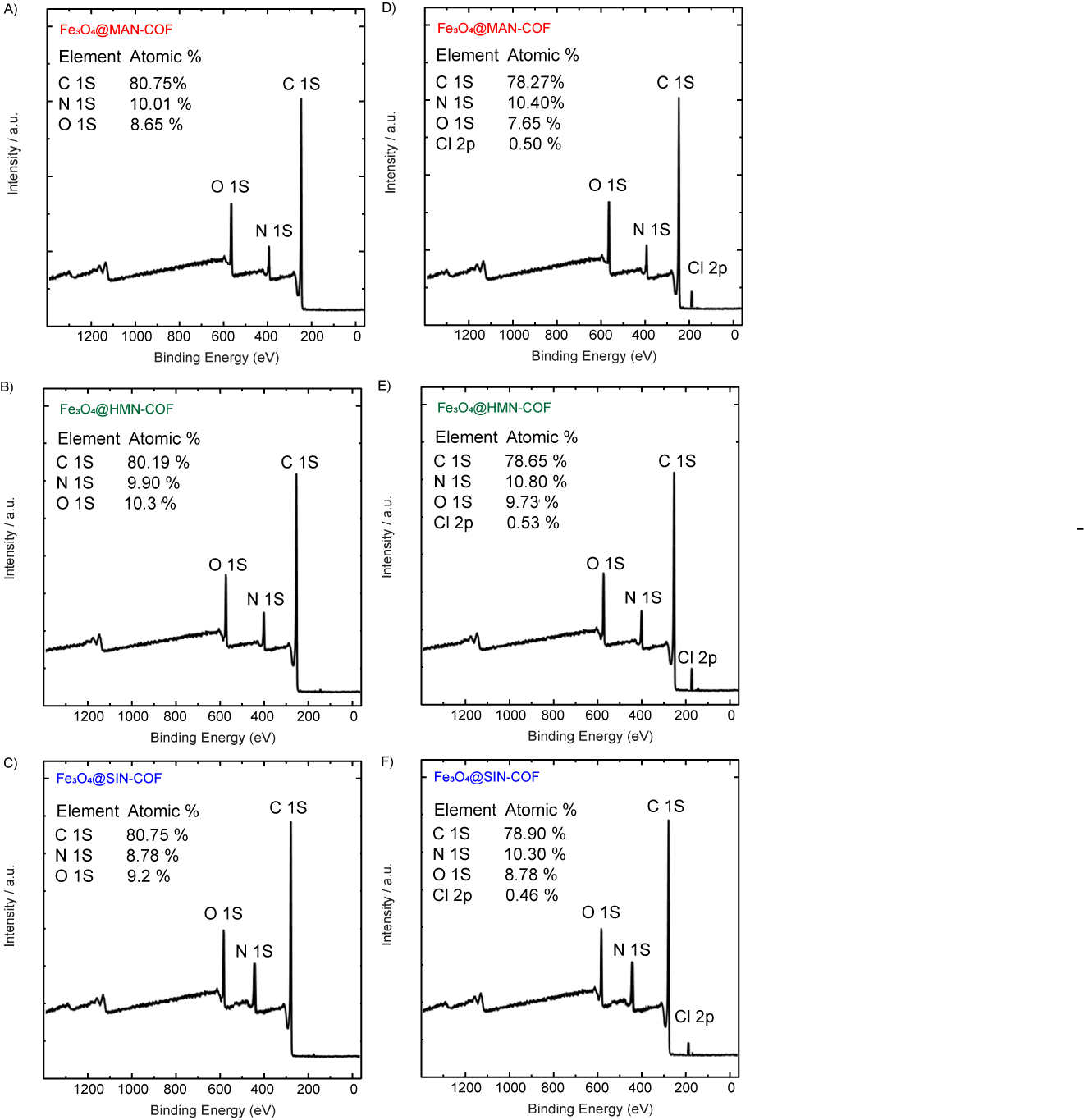


**Fig S15.** XPS spectra of Fe₃O₄@MAN-COF, Fe₃O₄@HMN-COF, and Fe₃O₄@SIN-COF composites. Panels A, B, and C show the spectra and atomic percentages of C 1S, N 1S, and O 1S for Fe₃O₄@MAN-COF, Fe₃O₄@HMN-COF, and Fe₃O₄@SIN-COF, respectively. Panels D, E, and F depict the same composites with additional peaks for Cl 2p, confirming the adsorption of imidacloprid. An increase in nitrogen content is also observed, further indicating successful adsorption of imidacloprid onto the COF materials.

**
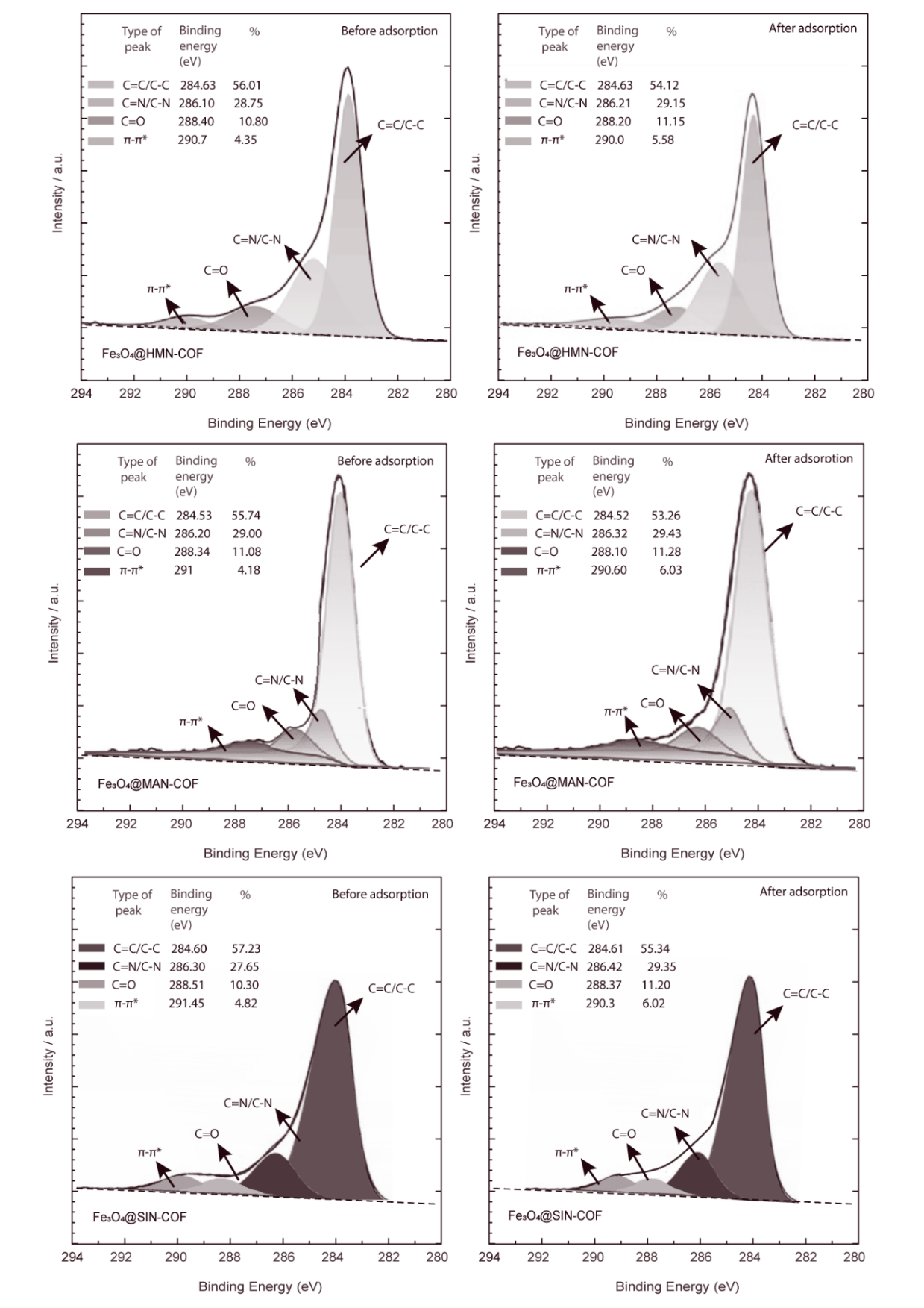
**

**Fig S16**. XPS C 1s spectra for Fe₃O₄@HMN-COF, Fe₃O₄@MAN-COF, and Fe₃O₄@SIN-COF before and after adsorption of Imidacloprid. The binding energy and relative percentage of various carbon species (C–C/C=C, C–N/C=N, C=O, π–π*) are indicated for each sample. Changes in peak intensities and binding energies highlight the interactions between Imidacloprid and the COFs.

**Section S7: Photocatalytic Degradation Studies**

The photocatalytic degradation of imidacloprid was studied using solutions of varying concentrations (10, 20, 30, 40, and 50 mg/L). These solutions were stirred under UV light with Fe₃O₄@HMN-COF, Fe₃O₄@MAN-COF, and Fe₃O₄@SIN-COF composites for specific intervals and analyzed using a UV-visible spectrophotometer at 268 nm to measure the residual imidacloprid concentration. Control experiments were conducted in the dark and without COF composites. Parameters such as irradiation time, initial concentration, catalyst dose, pH, and temperature were varied to study their effects on degradation efficiency.

**Effect of UV Irradiation Time.** The effect of UV irradiation time on the photocatalytic degradation was evaluated by stirring the imidacloprid solution at different time intervals (60, 120, 180, 240, and 300 minutes) in the presence of the catalysts under UV light.

**Effect of Initial Concentration.** The initial concentration of imidacloprid was varied (10 mg/L, 20 mg/L, 30 mg/L, 40 mg/L, and 50 mg/L) to study its effect on degradation. A 0.01 g portion of each Fe₃O₄-functionalized COF catalyst was added to 50 mL volumes of these solutions. The samples were stirred for a specific time interval under UV light and analyzed for imidacloprid concentration. Control experiments were conducted in the dark.

**Effect of Catalyst Dose.** To study the effect of catalyst dose, various amounts of Fe₃O₄@HMN-COF, Fe₃O₄@MAN-COF, and Fe₃O₄@SIN-COF composites (0.01 g, 0.02 g, 0.03 g, 0.04 g, and 0.05 g) were added to 50 mL volumes of imidacloprid solutions. The degradation was studied under UV light for a specific time interval.

**Effect of pH.** The effect of pH on the degradation process was examined by adjusting the pH of the imidacloprid solution to 3, 5, 7, 9, and 11 using HCl or NaOH solutions in the presence of 0.01 g of each catalyst. The solutions were stirred for an optimum time under both UV light and dark conditions.

**Effect of Temperature.** The effect of temperature on the photocatalytic degradation of imidacloprid was studied by varying the temperature of the pesticide solution from 20°C to 40°C in the presence of 0.01 g of each catalyst.

**Reusability and Stability.** The reusability of the catalysts was evaluated over five consecutive cycles by regenerating the catalysts after each cycle. The stability of the catalysts was also assessed through leaching experiments to ensure minimal loss of active components.


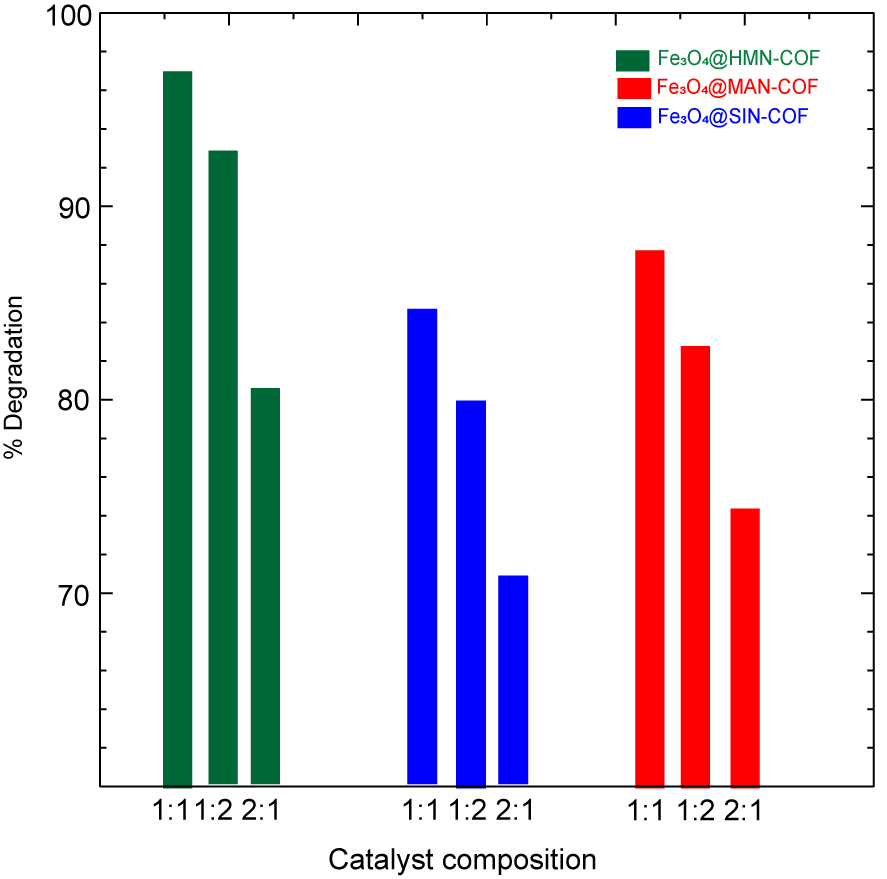


**Fig S17**. Percentage degradation of Imidacloprid using Fe₃O₄@HMN-COF, Fe₃O₄@MAN-COF, and Fe₃O₄@SIN-COF at different catalyst compositions (1:1, 1:2, and 2:1). Fe₃O₄@HMN-COF demonstrates the highest degradation efficiency across all compositions.


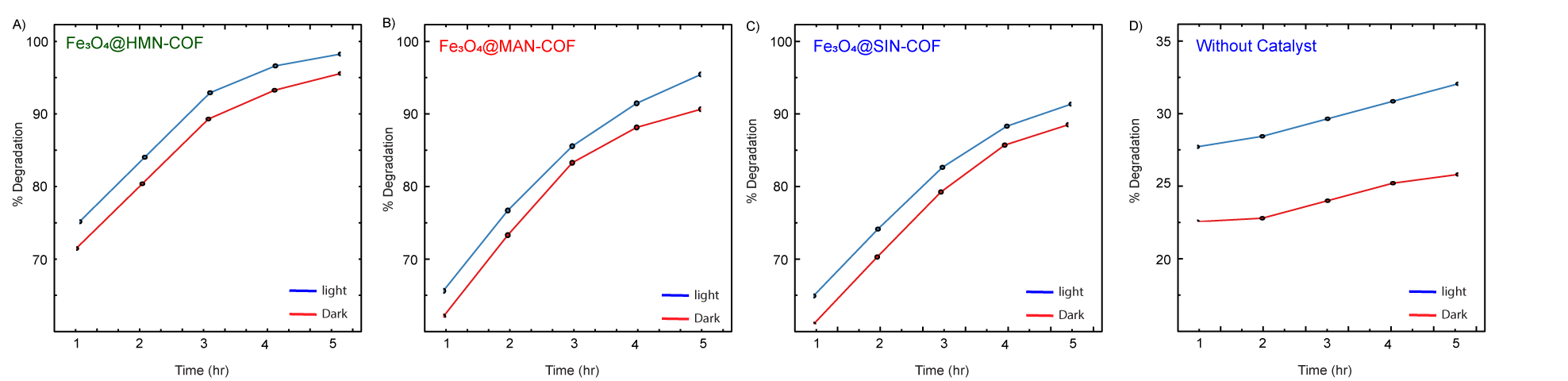


**Fig S18.** Degradation of Imidacloprid over time using (A) Fe₃O₄@HMN-COF, (B) Fe₃O₄@MAN-COF, (C) Fe₃O₄@SIN-COF, and (D) without catalyst under light and dark conditions. The presence of light significantly enhances the degradation efficiency for all COFs, with Fe₃O₄@HMN-COF showing the highest degradation rates. The control sample without catalyst exhibits minimal degradation.


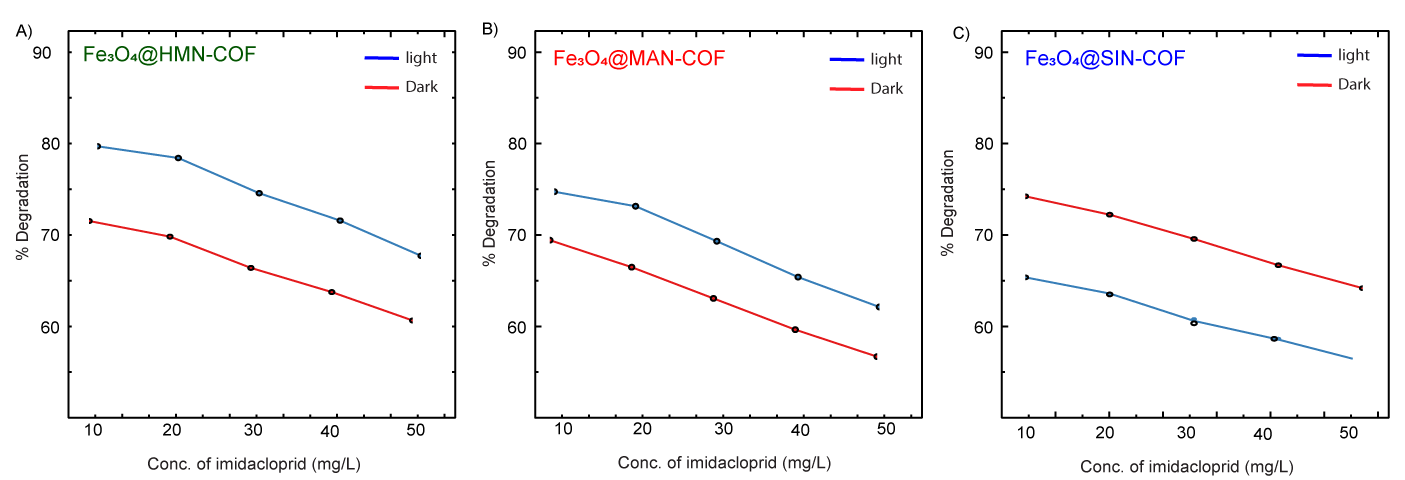


**Fig S19**. Degradation efficiency of Imidacloprid at various concentrations (10-50 mg/L) under light and dark conditions using (A) Fe₃O₄@HMN-COF, (B) Fe₃O₄@MAN-COF, and (C) Fe₃O₄@SIN-COF. All COFs show higher degradation under light conditions, with efficiency decreasing as the concentration of Imidacloprid increases. Fe₃O₄@HMN-COF demonstrates the highest overall degradation efficiency across all concentrations

**
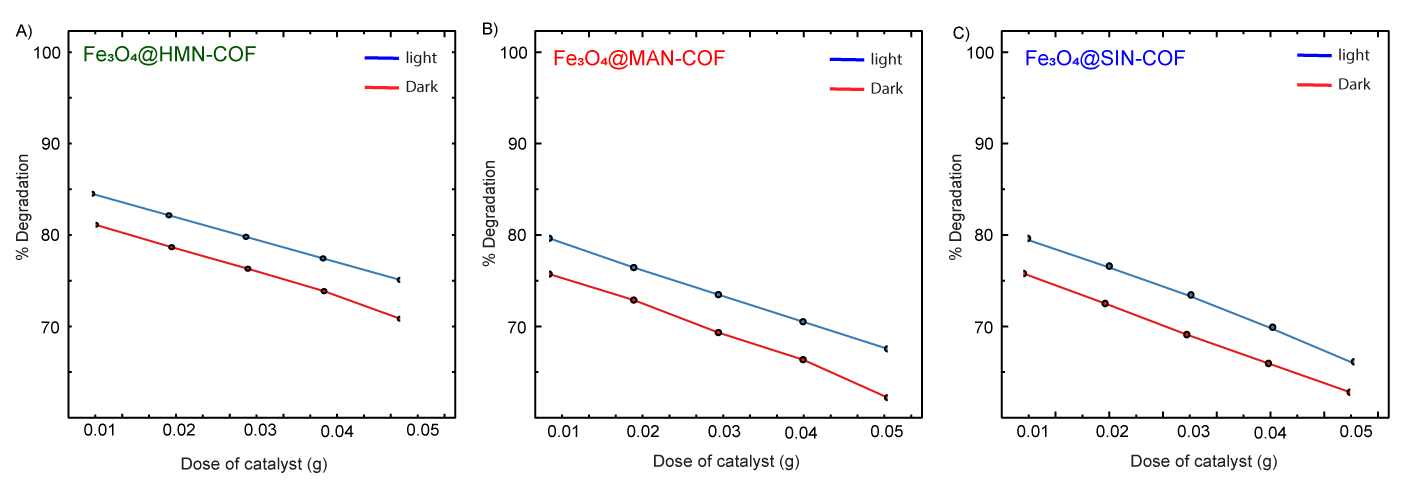
**

**Fig S20**. Degradation efficiency of Imidacloprid at various catalyst dosages (0.01-0.05 g) under light and dark conditions using (A) Fe₃O₄@HMN-COF, (B) Fe₃O₄@MAN-COF, and (C) Fe₃O₄@SIN-COF. All COFs show higher degradation under light conditions, with efficiency decreasing as the dose of the catalyst increases. Fe₃O₄@HMN-COF consistently demonstrates the highest degradation efficiency across all dosages.

**
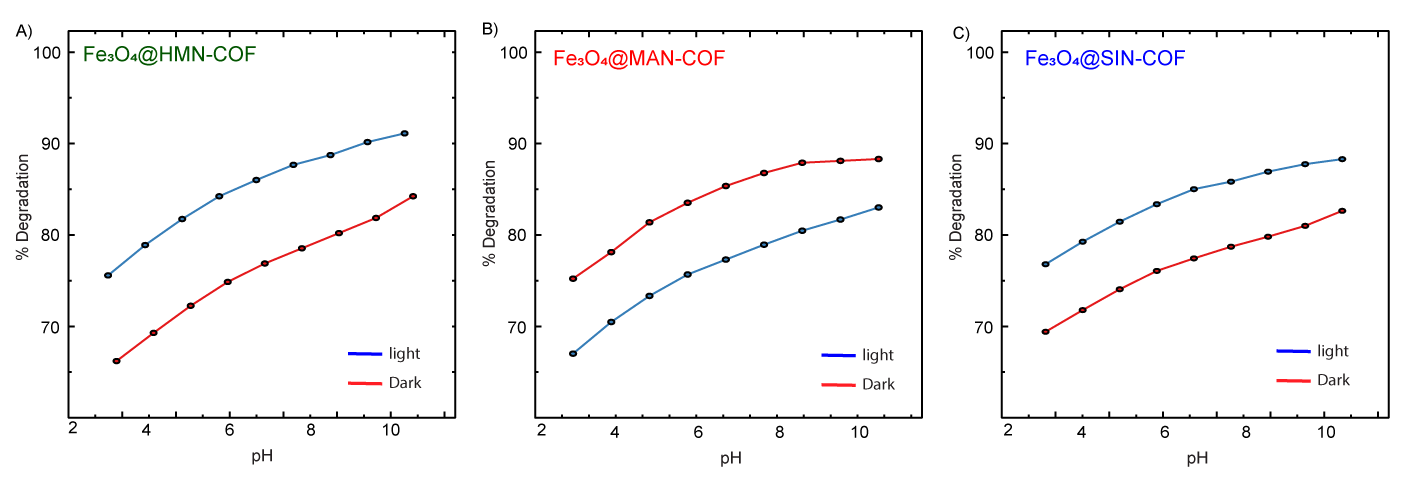
**

**Fig S21**. Degradation efficiency of Imidacloprid at various pH levels (2-10) under light and dark conditions using (A) Fe₃O₄@HMN-COF, (B) Fe₃O₄@MAN-COF, and (C) Fe₃O₄@SIN-COF. All COFs show higher degradation under light conditions, with efficiency increasing as the pH increases. Fe₃O₄@HMN-COF demonstrates the highest degradation efficiency across all pH levels.


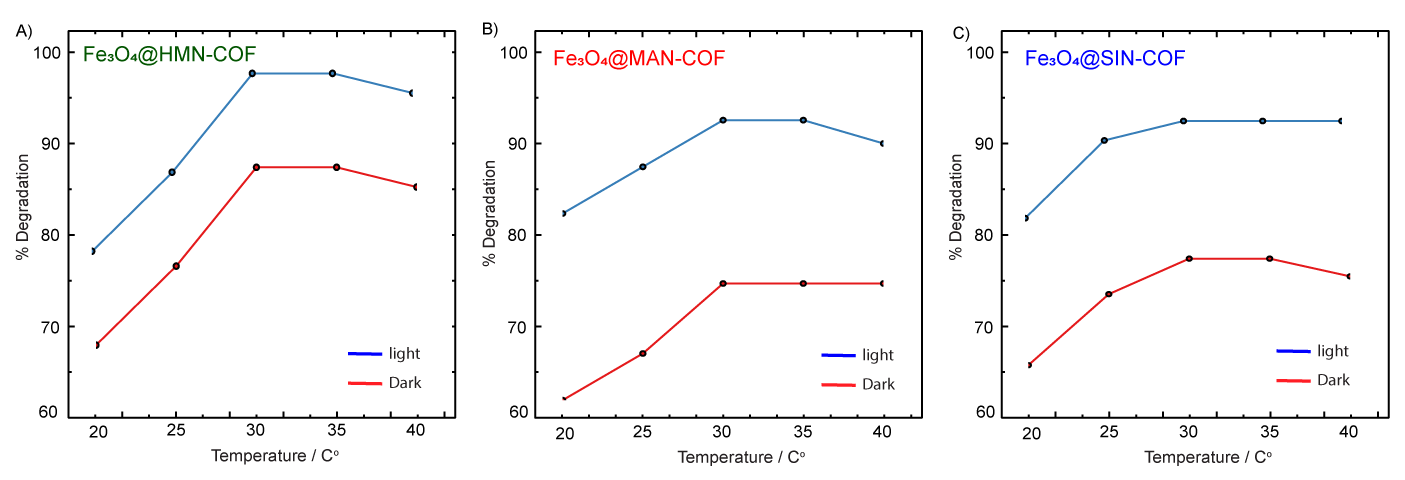


**Fig S22.** Degradation efficiency of Imidacloprid at various temperatures (20°C to 40°C) under light and dark conditions using (A) Fe₃O₄@HMN-COF, (B) Fe₃O₄@MAN-COF, and (C) Fe₃O₄@SIN-COF. All COFs show higher degradation under light conditions, with efficiency generally increasing with temperature. Fe₃O₄@HMN-COF demonstrates the highest degradation efficiency, particularly at 30°C and 35°C.

B

A

D

C

E

**Fig S23**. Kinetic plots for the degradation of imidacloprid using Fe₃O₄-functionalized COFs under light and dark conditions. (A) Kinetic plots without a catalyst in the dark, (B) Kinetic plots without a catalyst in light, (C) Second-order kinetic plots for Fe₃O₄@MAN-COF (light and dark), (D) Second-order kinetic plots for Fe₃O₄@HMN-COF (light and dark), (E) Second-order kinetic plots for Fe₃O₄@SIN-COF (light and dark). The plots show the natural logarithm of the concentration of imidacloprid (ln(C/C₀)) versus time, indicating the rate constants and correlation coefficients (R²) for each condition. The data illustrate the enhanced degradation kinetics under light conditions for all COFs.


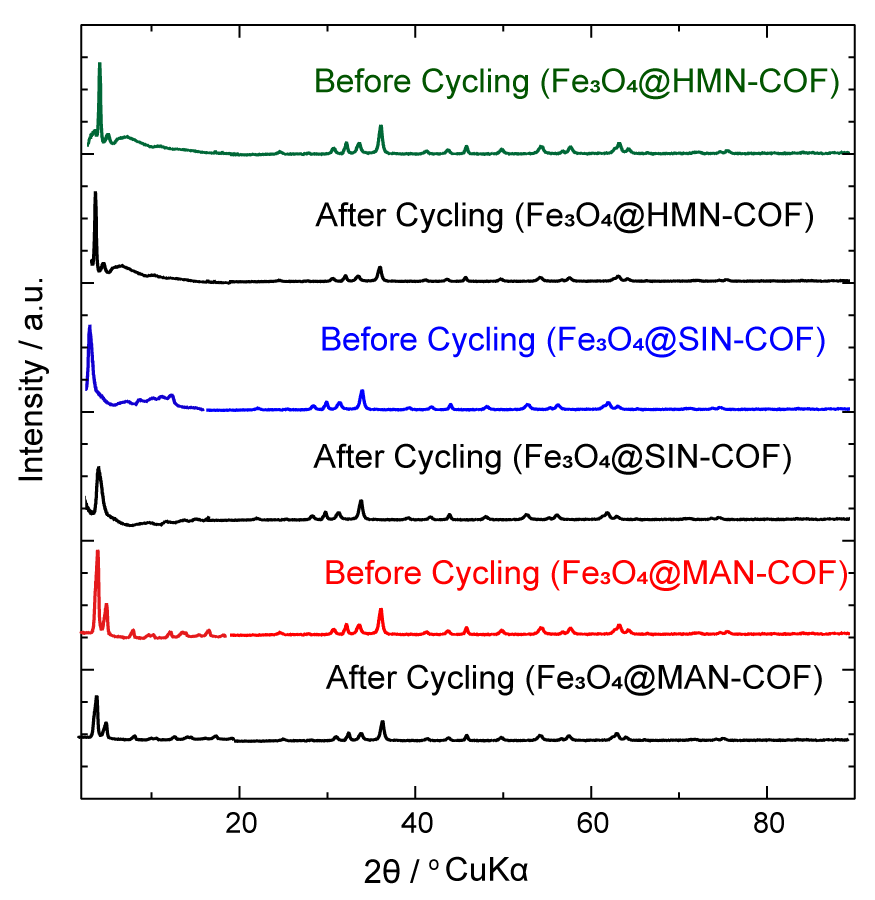


**Fig S24.** XRD patterns of Fe₃O₄@HMN-COF, Fe₃O₄@SIN-COF, and Fe₃O₄@MAN-COF before and after cycling. The comparison illustrates the structural stability of the COFs after repeated adsorption-desorption cycles. The consistent peak positions indicate that the COFs maintain their crystalline structure after cycling.

**Section S8: References**

1. Fathi, A.A., et al., Development of dispersive micro solid phase extraction method based on using Fe₃O₄@UiO-66-NH₂@MIP nanocomposite as an efficient and selective sorbent for the extraction of imidacloprid from fruit juice samples. *Microchemical Journal*, 2023. 187: p. 108427.
2. Mohammad, S.G. and M.M.H. El-Sayed, Removal of imidacloprid pesticide using nanoporous activated carbons produced via pyrolysis of peach stone agricultural wastes. *Chemical Engineering Communications*, 2021. 208(8): p. 1069-1080.
3. Ma, Y., et al., Adsorptive removal of imidacloprid by potassium hydroxide activated magnetic sugarcane bagasse biochar: Adsorption efficiency, mechanism and regeneration. *Journal of Cleaner Production*, 2021. 292: p. 126005.
4. Srikhaow, A., et al., Adsorption Kinetics of Imidacloprid, Acetamiprid and Methomyl Pesticides in Aqueous Solution onto Eucalyptus Woodchip Derived Biochar. *Minerals*, 2022. 12(5): p. 528.
5. Mohammad, S.G., et al., Porous Activated Carbon from Lignocellulosic Agricultural Waste for the Removal of Acetampirid Pesticide from Aqueous Solutions. *Molecules*, 2020. 25(10): p. 2339.
6. Selahle, S.K., A. Mpupa, and P.N. Nomngongo, Combination of zeolitic imidazolate framework-67 and magnetic porous porphyrin organic polymer for preconcentration of neonicotinoid insecticides in river water. *Journal of Chromatography A*, 2022. 1661: p. 462685.
7. Ozalp, O., Z.P. Gumus, and M. Soylak, MIL-101(Cr) metal–organic frameworks based on deep eutectic solvent (ChCl: Urea) for solid phase extraction of imidacloprid in tea infusions and water samples. *Journal of Molecular Liquids*, 2023. 378: p. 121589.
8. Yang, Y., et al., Eco-friendly and acid-resistant magnetic porous carbon derived from ZIF-67 and corn stalk waste for effective removal of imidacloprid and thiamethoxam from water. *Chemical Engineering Journal*, 2022. 430: p. 132999.
9. Jevremović, A., et al., Mitigating toxicity of acetamiprid removal techniques – Fe modified zeolites in focus. *Journal of Hazardous Materials*, 2022. 436: p. 129226.
10. Yan, W., et al., An urchin-shaped covalent organic framework with rich nitrogen for efficient removal of neonicotinoid insecticides in honey and fruits. *Food Chemistry*, 2023. 429: p. 136872
